# Outbreak of highly pathogenic avian influenza A(H5N1) clade 2.3.4.4b virus in cats, Poland, June to July 2023

**DOI:** 10.2807/1560-7917.ES.2023.28.31.2300366

**Published:** 2023-08-03

**Authors:** Katarzyna Domańska-Blicharz, Edyta Świętoń, Agnieszka Świątalska, Isabella Monne, Alice Fusaro, Karolina Tarasiuk, Krzysztof Wyrostek, Natalia Styś-Fijoł, Aleksandra Giza, Marta Pietruk, Bianca Zechchin, Ambra Pastori, Łukasz Adaszek, Małgorzata Pomorska-Mól, Grzegorz Tomczyk, Calogero Terregino, Stanisław Winiarczyk

**Affiliations:** 1 Department of Poultry Diseases, National Veterinary Research Institute, Puławy, Poland; 2 Department of Omic Analyses, National Veterinary Research Institute, Puławy, Poland; 3 Department of Veterinary Hygiene, Gdańsk, Poland; 4 Istituto Zooprofilattico Sperimentale delle Venezie (IZSVe), Legnaro, Italy; 5 Department of Epizootiology and Clinic of Infectious Diseases, Faculty of Veterinary Medicine, University of Life Sciences, Lublin, Poland; 6 Department of Preclinical Sciences and Infectious Diseases, University of Life Sciences, Poznan, Poland; 7 Director General, National Veterinary Research Institute, Puławy, Poland

**Keywords:** HPAI, H5N1, cats, Poland, avian influenza, veterinary

## Abstract

**Background:**

Over a 3-week period in late June/early July 2023, Poland experienced an outbreak caused by highly pathogenic avian influenza (HPAI) A(H5N1) virus in cats.

**Aim:**

This study aimed to characterise the identified virus and investigate possible sources of infection.

**Methods:**

We performed next generation sequencing and phylogenetic analysis of detected viruses in cats.

**Results:**

We sampled 46 cats, and 25 tested positive for avian influenza virus. The identified viruses belong to clade 2.3.4.4b, genotype CH (H5N1 A/Eurasian wigeon/Netherlands/3/2022-like). In Poland, this genotype was responsible for several poultry outbreaks between December 2022 and January 2023 and has been identified only sporadically since February 2023. Viruses from cats were very similar to each other, indicating one common source of infection. In addition, the most closely related virus was detected in a dead white stork in early June. Influenza A(H5N1) viruses from cats possessed two amino acid substitutions in the PB2 protein (526R and 627K) which are two molecular markers of virus adaptation in mammals. The virus detected in the white stork presented one of those mutations (627K), which suggests that the virus that had spilled over to cats was already partially adapted to mammalian species.

**Conclusion:**

The scale of HPAI H5N1 virus infection in cats in Poland is worrying. One of the possible sources seems to be poultry meat, but to date no such meat has been identified with certainty. Surveillance should be stepped up on poultry, but also on certain species of farmed mammals kept close to infected poultry farms.

**Key public health message**

**What did you want to address in this study and why?**

There was widespread information on social media in Poland about a violent, fatal cat disease with acute respiratory and neurological signs probably caused by avian influenza virus. The aim of the ongoing investigations was to confirm or exclude this information, characterise the identified virus and investigate possible sources of infection.

**What have we learnt from this study?**

We detected the highly pathogenic avian influenza virus of the H5N1 subtype in 25 of 46 cats. Molecular analyses showed that it belongs to the genotype of a virus that previously circulated in wild birds and poultry in Poland and also contains several mutations that may increase adaptation to mammals. The viruses were very similar to each other, indicating a common source of infection, which, however, has not yet been identified.

**What are the implications of your findings for public health?**

The presence of similar viruses with mammalian-adapted features in so many cats is highly concerning. Although there are no reports of humans infected with this virus, such a risk exists especially for cat owners. Therefore, it is recommended to observe the health of feline pets, limit their contact with the outside environment and avoid feeding them raw poultry meat.

## Introduction

Europe, and more recently the Americas, have been experiencing highly pathogenic avian influenza (HPAI) virus infections of an unprecedented scale in wild birds and poultry [1,2]. The most important variations in the course of the 2021/22 epidemic season were the lack of quiescence of the infections during the summer and their continuation into the 2022/23 season. Moreover, the currently circulating Eurasian HPAI A subtype H5N1 virus has been identified in a wide range of wild mammals as foxes, lynxes, skunks, raccoons, bears, otters, polecats, badgers, ferrets, pumas, panthers, opossums, seals, porpoises and sea lions, as well as dolphins. In the vast majority of these cases, mammals, including marine mammals such as seals and porpoises, were carnivorous predators [1].

During the 2022/23 epidemic season in Poland (from 21 September 2022 until 10 July 2023), HPAI H5N1 virus was detected in 93 outbreaks in poultry and 147 outbreaks in wild birds. The first outbreak in Poland occurred on 21 September 2022 in Łódź voivodeship. This single outbreak was followed by a 2.5-month break, as the next HPAI virus infection was detected in early December, followed by 36 further outbreaks in poultry. The infections continued in 2023, with a total of 39 outbreaks in January, 17 in February, then only two outbreaks in March, followed by one outbreak in May and the most recent one in a backyard flock on 1 July. 

The vast majority of outbreaks were caused by HPAI H5N1 virus CH genotype. The CH (H5N1 A/EurasianWigeon/Netherlands/3/2022-like) genotype in Poland was identified for the first time in mid-December 2022 and has since then been responsible for 58% of cases in domestic birds and 30% of cases in wild birds (mainly waterfowl) [1]. Between December 2022 and January 2023, it was responsible for several outbreaks in poultry mainly in the Wielkopolskie region, while since February 2023, this genotype has only been identified sporadically in the country (n = 5) when it was replaced with the gull-adapted BB (H5N1-A/gull/France/22PO15977/2022-like) genotype [3]. 

The BB genotype was identified in the two most recent poultry outbreaks: in May in a turkey flock and in June in a backyard flock. In wild birds, a total of 147 outbreaks were recorded, 12 in autumn 2022 and 135 in 2023. Outbreaks up to February 2023 were mainly recorded in wild birds of the order *Anseriformes* (n = 37) such as mute swans, graylag and bean goose, while later outbreaks included only black-headed gulls and other birds of the *Laridae* family (n = 96) and were caused by infection with the HPAI H5N1 virus BB genotype. On 4 June 2023, a dead white stork was found, infected with HPAI virus of CH (H5N1_A/Eurasian_Wigeon/Netherlands/3/2022-like) genotype, and on 20 June 2023 a mute swan where the amount of retrieved virus material was too small to perform whole genome sequencing. In addition to the CH and BB genotypes, the AB genotype (A/duck/Saratov/29–02/2021-like) was also detected in poultry and wild birds between December 2022 and March 2023, but to a lesser extent.

Here, we describe an outbreak caused by HPAI H5N1 virus detected in 25 cats from different regions of Poland over a period of 3 weeks between the end of June and the beginning of July 2023.

## Methods

### Setting

A national programme aimed at detection of HPAI virus infections in poultry and wild birds has been conducted in Poland for 20 years, since 2003. The obligation to conduct these surveys, as well as the detailed manner and mode of control of this disease, derives from the provisions of Commission Delegated Regulation (EU) 2020/689 of 17 December 2019 supplementing Regulation (EU) 2016/429 of the European Parliament and of the Council [4]. It includes both passive surveillance of HPAI virus in poultry and wild birds (testing of dead and sick birds) and active surveillance testing of live poultry from species that do not show typical symptoms following HPAI virus infection, as well as active surveillance of low pathogenic avian influenza virus (serology). It is mandatory that all detected infections are notified, sequencing is performed on representative samples, and the sequences are then forwarded to the European Union Reference Laboratory (EURL) for Avian Influenza in Italy (Istituto Zooprofilattico Sperimentale delle Venezie) [5]. Apart from the aforementioned monitoring and veterinary surveillance in slaughterhouses for the clinical health status of animals, there are no other measures in place to prevent avian influenza entering the food chain. There is no monitoring of avian influenza infections in mammals in Poland.

### Epidemiological investigation

Information about a highly fatal disease in cats with respiratory and nervous system signs began to circulate in social media and among cat lovers in mid-June 2023. At the time, all of the information came from the media; there was no complete knowledge, let alone certainty, of what had actually happened. In order to systematise the research and to obtain detailed data on cats, we developed a questionnaire for practitioners at veterinary clinics, in which we asked about various aspects of cat health, behaviour, nutrition, clinical manifestations or gross lesions. Information was also given on which samples to take from dead (whole cats/organ fragments, especially brain and respiratory tissues) or live (throat and rectal swabs) cats. Instructions on how to send samples for testing were also given (refrigerated if anticipated transport was 2–3 days, frozen if longer). A few veterinary clinics sent samples to the laboratory together with questionnaires; such samples were in addition sent directly by cat owners. The information received in the questionnaires as well as interviews with cat owners were thoroughly analysed. 

### Laboratory investigation

Swabs were immersed in phosphate buffered saline (PBS) and organs were homogenised in PBS to 20% weight/volume suspensions. The RNA was extracted using IndiMag Pathogen Kit in IndiMag 48s (Indical Bioscience). Samples were tested with real-time RT-PCR targeting the M gene of influenza A virus [6], and subtyping was performed with primers and probes specific for H5 and N1 genes [7,8]. From each cat, the samples with the highest viral load (n = 23) were subjected to whole genome sequencing as previously described [9]. Libraries were sequenced in NextSeq 550 or iSeq 100 (Illumina). Consensus sequences were generated and compared with previously obtained HPAI H5N1 virus genomes from Poland, as well as sequences from other European countries available in GISAID. Viral sequences from birds in Poland available in GISAID are listed in Supplementary Table S1. Maximum likelihood phylogenetic trees were generated for each genome segment using IQ-TREE [10,11] with 1,000 ultra-fast bootstrap replications and visualised with FigTree v1.4.4 (http://tree.bio.ed.ac.uk/software/figtree).

The phylogenetic network was generated using the median joining method implemented in NETWORK 10.2.0.0 [12] for the eight concatenated gene segments of all non-reassortant HPAI H5N1 viruses collected in Europe that belonged to the same genotype of the viruses from cats (genotype CH). This allowed us to visualise how the viral genomes are connected on the basis of their genetic similarity.

## Results

### Epidemiological investigation

**Figure 1:**
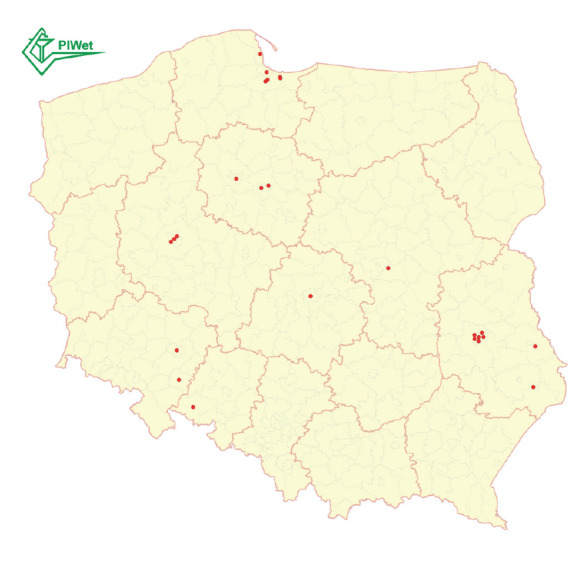
Locations of cats infected with highly pathogenic avian influenza A(H5N1) virus, Poland, June–July 2023 (n = 25)

The disease was reported from different locations across the vast area of Poland, mainly in big cities (Figure 1). The samples from cats were collected over a time period of a few days, the earliest on 14 June and the latest on 23 June 2023 (Table 1). 

Locations are marked as red dots.

**Table 1:** Information on cats positive for highly pathogenic avian influenza A(H5N1) virus, Poland, June–July 2023 (n = 25)

Cat	Collection date	Analysis date	Isolate ID	Origin	GISAID number	Description of cat keeping			Studied samples	rRT-PCR (Cq value)		
						Outdoor/non-outdoor	Feeding method	Other		M	H5	N1
1	19 June	22 June	H265-Kot1/23	Poznań	EPI_ISL_17951056	Non-outdoor	Raw meat	n/a	TS	31.90	32.40	33.39
2	19 June	22 June	H265-Kot2/23	Poznań	EPI_ISL_17985196	Non-outdoor	Raw meat	n/a	TS	30.41	31.10	32.00
3	21 June	22 June	H246/23	Gdynia	EPI_ISL_17956378	n/a	n/a	Homeless, found dead near woodlots	TS	35.46	35.67	36.39
									Brain	31.06	30.47	31.48
4	20 June	22 June	H247/23	Błotnik (Pruszcz Gdański)	EPI_ISL_17951055	n/a	n/a	n/a	TS	27.72	27.66	27.95
									Brain	15.23	16.80	16.93
									IO	29.59	31.56	30.95
5	14 June	22 June	H248/23	Błotnik (Pruszcz Gdański)	EPI_ISL_17951054	n/a	n/a	Backyard cat, bit man, then died	TS	27.99	28.27	28.03
									Brain	9.48	10.38	10.50
									IO	22.87	24.64	24.20
6	19 June	22 June	H249/23	Gdańsk	EPI_ISL_17950995	Non-outdoor; sometimes goes out on the balcony	Mainly dry food but sometimes raw poultry meat	Age 7 years, Russian blue	TS	17.53	20.19	20.48
									Brain	15.71	16.83	17.23
									IO	18.49	20.61	20.97
7	16 June	23 June	H252/23	Lublin	EPI_ISL_17951051	n/a	n/a	Age 3 years, male castrated	NS	27.49	26.91	27.40
									TS	28.16	27.62	28.21
									RS	32.68	33.13	33.21
8	18 June	23 June	H253/23	Lublin	EPI_ISL_17951052	Non-outdoor	Dry and wet cat food	Age 2.5 years, female sterilised	NS	28.89	29.92	30.91
									TS	28.43	29.2	29.92
									RS	35.56	nd	37.09
9	17 June	23 June	H254/23	Lublin	EPI_ISL_17951053	n/a	n/a	Age 6 years, male castrated	NS	23.41	22.90	23.90
									TS	23.29	23.74	23.73
									RS	35.45	nd	23.73
10	23 June	26 June	H255/23	Pruszcz Gdański	EPI_ISL_17971989	n/a	n/a	n/a	IO	17.52	19.13	18.70
									Brain	13.06	13.99	14.89
11	23 June	26 June	H256/23	Kamień	EPI_ISL_17971990	n/a	n/a	Age 1 year	NS	27.35	27.40	28.34
									TS	23.09	23.36	24.32
12	23 June	27 June	H257/23	Lublin	EPI_ISL_17971991	Non-outdoor	Raw poultry meat	Age 3 months	TS	31.30	31.89	32.12
13	n/a	28 June	H263/23	Komarów (zamojski)	EPI_ISL_17971992	n/a	Raw poultry meat	Age 1 year	TS	28.69	25.84	25.87
									NS	28.52	28.60	28.81
									RS	26.78	27.85	28.45
14	21 June	28 June	H264/23	Poznań	EPI_ISL_17971998	Outdoor	Raw poultry meat	Age 8 years, male	TS	19.78	20.58	20.75
15	n/a	28 June	H266/23	Bydgoszcz	EPI_ISL_17971993	Outdoor	n/a	Age 8 years, European	Liver	17.66	17.65	18.83
									Stomach	33.30	nd	33.41
16	n/a	29 June	H267/23	Strzelin	EPI_ISL_17971994	n/a	Raw poultry meat	Age 5 years, British shorthair	TS	28.49	28.68	29.86
17	n/a	29 June	H270/23	Lublin	EPI_ISL_17971995	n/a	Raw poultry meat	Age 10 weeks, Singapura	TS	31.86	32.80	33.52
18	n/a	29 June	H271/23	Lublin	EPI_ISL_17971996	n/a	Raw poultry meat	Age 3 years, European	TS	30.48	30.62	31.56
19	n/a	29 June	H275/23	Pruszcz Gdański	Not sequenced	n/a	n/a	n/a	IO	28.05	29.94	29.54
									Brain	12.97	14.15	14.32
20	n/a	29 June	H277/23	Wrocław	EPI_ISL_17971997	n/a	Raw poultry meat	Age 9 years, European	TS	31.74	32.09	32.57
									IO	19.94	21.96	23.01
21	n/a	30 June	H293/23	Nysa	Not sequenced	n/a	n/a	n/a	NS	30.67	31.89	33.96
22	n/a	4 July	H299/23	Wilcza Wola (piaseczynski)	Not sequenced	n/a	n/a	Age 4 years, male European	NS	24.25	24.75	24.66
									TS	29.69	30.27	30.01
23	n/a	6 July	H303/23	Łódź	Not sequenced	Non-outdoor	Raw poultry meat	Age 1 year, caracal^a^	Lung swab	21.52	23.53	23.81
24	16 June	6 July	H304–1/23	Torun	Not sequenced	Outdoor	BARF diet	Age 6–8 weeks	NS	19.42	18.90	20.61
									TS	21.53	21.91	23.92
									Lungs	16.94	16.97	18.96
									Brain	9.18	9.80	10.47
25	16 June	6 July	H304–2/23	Torun	Not sequenced	Outdoor	BARF diet	Age 12 years	NS	28.71	28.40	30.42
									TS	28.35	31.45	33.19
									Lungs	20.81	21.85	23.99
									Brain	14.87	15.23	16.76

BARF: biologically appropriate raw food; Cq: quantification cycle; IO: internal organs; n/a: not available; nd: not detected; NS: nasal swab; RS: rectal swab; TS: throat swab.

^a^ Exotic animal kept as pet.

**Figure 2:**
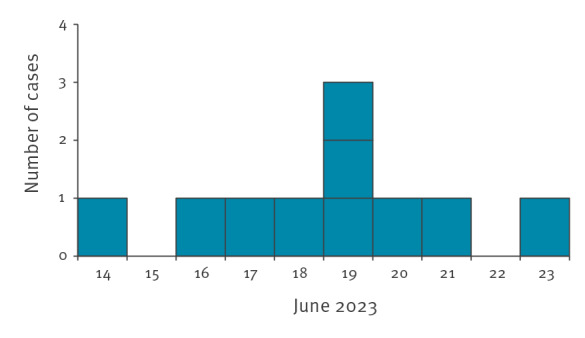
Epidemic curve of cats infected with highly pathogenic avian influenza A(H5N1) virus, Poland, June 2023 (n = 25)

Analysis of the epidemic curve over time suggests that the peak of infections was around 18–20 June (Figure 2). Of the 46 cats tested during 14–23 June, 25 were infected. The infected cats were of a wide range of ages between 6 weeks and 12 years, different sex and breeds. They were fed a variety of foods, 12 of 25 had fresh, raw poultry meat in their diet, two were fed a biologically appropriate raw food (BARF) diet, and for 11 cats no such data were obtained. Regarding contact with the environment, six cats were kept only indoors or had limited access to the outside environment, four cats went outside, two were backyard cats and for 13 there was no such information. The questionnaires received showed that for cats fed raw, fresh poultry meat, the first clinical signs (apathy, fever) usually appeared a few days (2–3 days) after meat consumption. The course of the infection ended with the death of the cat (either a natural death or euthanasia).

The course of the disease was similar in all cases: loss of appetite, apathy, hypersalivation, fever, dyspnoea (shallow and accelerated breathing), hard and painful abdomen, sometimes incontinence of urine, reddened mucous membranes, trismus, followed by nervous symptoms such as epileptic seizures, increased muscle tension and sometimes stiffness of the limbs. On clinical examination, an exacerbated vesicular murmur and constricted pupils unresponsive to light were noted; an illustrative picture is appended as Supplementary Figure S2. Attempts to treat pneumonia with various antibiotics were not successful and the conditions of the animals worsened after 1 or 2 days. In most cases, the animals were euthanised. 

Post-mortem examination in 11 cats revealed the presence of lesions in every organ, which were congested, sometimes swollen with the presence of bloody fluid (a detailed description of the lesions is included in Supplementary Material S3).

### Laboratory investigation

Due to emerging nervous signs, the cats were initially examined for rabies, and then the samples were screened for the presence of avian influenza virus. Influenza A (H5N1) virus was identified and was present in some organs and tissues with a very high viral load (based on quantification cycle values) (Table 1).

**Figure 3:**
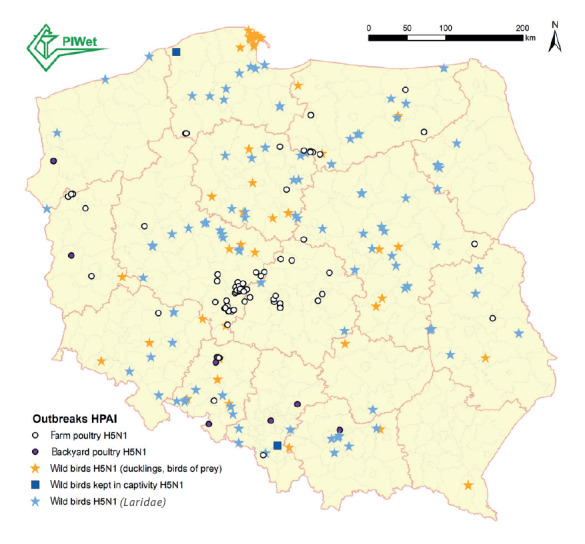
Location of outbreaks of highly pathogenic avian influenza A(H5N1) virus in poultry and wild birds, Poland, during the 2022/23 season

Since the HPAI H5N1 virus outbreak detected in cats cannot be discussed without outlining the influenza situation in poultry and wild birds in Poland, we also present a map with the locations where these infections occurred in Figure 3, in addition to the data in the Introduction section. The viral sequences obtained from representative bird samples were also submitted to GISAID and are listed in Supplementary Table S1.

HPAI: highly pathogenic avian influenza.

At the time of writing this article, we had performed whole genome sequencing on 19 of the HPAI H5N1 virus-positive samples collected from 25 cats (Table 1); sequencing of the remaining positive samples is planned/underway. Phylogenetic analysis was performed using sequences both from cats and birds in Poland as listed in Supplementary Table S1. Maximum likelihood phylogenetic trees of the eight gene segments are made available in Supplementary Figure S4. Their topology indicates that the HPAI H5N1 viruses collected from the cats belonged to the CH (H5N1_A/Eurasian_Wigeon/Netherlands/3/2022-like) genotype. The sequences of the viruses from cats are highly related to each other and clustered with a virus of the same genotype detected in a white stork in Poland on 4 June 2023 (A/white_stork/Poland/MB244/2023). 

**Figure 4:**
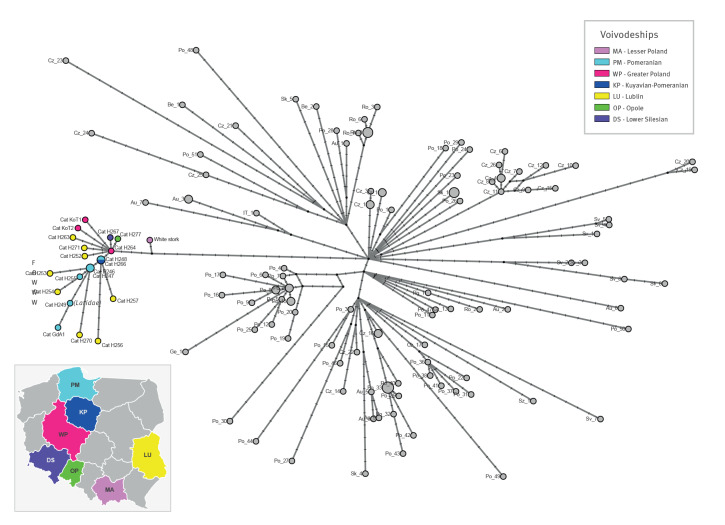
Genetic network of complete genome sequences of highly pathogenic avian influenza A(H5N1) viruses^a^ of the CH genotype collected from birds in Europe and from cats (n = 19) in Poland, June–July 2023

DS: Lower Silesian; KP: Kuyavian-Pomeranian; LU: Lublin; MA: Lesser Poland; OP: Opole; WP: Greater Poland.

^a^ H5N1 A/Eurasian wigeon/Netherlands/3/2022-like genotype from Europe.

No clustering by geographical region was observed for the HPAI H5N1 viruses collected from the 25 cats (Figure 4). The HPAI H5N1 viruses collected from these cats differed by 1–12 nucleotides and by 0–8 amino acids (Table 2); additional detail is provided in Supplementary Table S5. Compared with the most closely related viruses collected from birds (A/white_stork/Poland/MB244/2023), the viruses from cats showed at least four nucleotide and three amino acid mutations (PB2-N82T, PB2-K526R and PB1-P64S).

Each circle represents a unique genome where the diameter is proportional to the number of viruses sharing that particular genome. Circle representing the genome of the H5N1 viruses of the CH genotype from Europe are coloured in grey, while viruses collected from cats are coloured according to the voivodeship of collection.

Viral sequences from birds in Poland available in GISAID are listed in Supplementary Table S1.

**Table 2:** Amino acid differences among the proteins of influenza A(H5N1) viruses collected from cats, Poland, June–July 2023 (n = 19)

Amino acid differences	PB2			PB1					PA				HA		NA		M1		M2	NS1
	443	472	649	47	111	112	211	382	105	277	475	704	11	67	143	450	33	207	55	36
A/domestic cat/Poland/H246-M/2023 H5N1 2023–06–21 Gdynia	K	E	V	H	**I**	**D**	R	N	F	S	A	A	V	I	K	S	A	S	L	L
A/domestic cat/Poland/H255-M/2023 H5N1 2023–06–21 Pruszcz	K	E	V	H	**I**	**D**	R	N	F	S	A	A	V	I	K	S	A	S	L	L
A/domestic cat/Poland/H256-G/2023 H5N1 2023–06–24 Lublin	K	E	V	H	**I**	E	R	N	F	S	A	A	V	**V**	K	S	**V**	S	**F**	L
A/domestic cat/Poland/H257-G/2023 H5N1 2023–06–24 Lublin	K	E	V	H	**I**	E	R	N	F	S	**T**	A	V	I	K	S	A	S	L	L
A/domestic cat/Poland/H263-G/2023 H5N1 2023–06–26 Komarow	K	E	V	H	M	E	R	N	F	S	A	**T**	V	I	K	**G**	A	S	L	L
A/domestic cat/Poland/H264-G/2023 H5N1 2023–06–26 Poznan	K	E	V	H	M	E	R	N	F	S	A	A	V	I	K	S	A	S	L	L
A/domestic cat/Poland/H266-W/2023 H5N1 2023–06–19 Bydgoszcz	K	E	V	H	**I**	E	R	N	F	S	A	A	V	I	K	S	A	S	L	L
A/domestic cat/Poland/H267-W/2023 H5N1 Strzelin	K	E	V	H	M	E	R	N	F	S	A	A	V	I	K	S	A	S	L	L
A/domestic cat/Poland/H270-W/2023 H5N1 lubelskie	K	E	V	H	**I**	**D**	R	**D**	F	**Y**	A	A	V	I	**R**	S	A	S	L	L
A/domestic cat/Poland/H271-W/2023 H5N1 lubelskie	K	E	V	H	M	E	R	N	F	S	A	A	V	I	K	S	A	S	L	L
A/domestic cat/Poland/H277-W1/2023 H5N1 2023–06–26 Namyslow	K	E	V	H	M	E	R	N	F	S	A	A	V	I	K	S	A	S	L	L
A/domestic cat/Poland/H249/2023 H5N1 2023–06–22 Gdansk	**R**	**D**	V	H	**I**	**D**	R	N	F	S	A	A	V	I	K	S	A	S	L	L
A/domestic cat/Poland/H248/2023 H5N1 2023–06–15 Pruszcz Gd	K	E	V	H	M/I	E	R	N	F	S	A	A	V	I	K	S	A	S	L	L
A/domestic cat/Poland/Kot2/2023 H5N1 2023–06–19 Poznan	K	E	**M**	H	M	E	R	N	/	/	/	A	V	I	K	S	A	**N**	L	L
A/domestic cat/Poland/Kot1/2023 H5N1 2023–06–19 Poznan	K	E	V	**Y**	M	E	**K**	N	F	S	A	A	V	I	K	S	A	S	L	**I**
A/domestic cat/Poland/H254/2023 H5N1 2023–06–22 Lublin	K	E	V	H	**I**	**D**	R	N	**L**	S	A	A	V	I	K	S	A	S	L	L
A/domestic cat/Poland/H252/2023 H5N1 2023–06–22 Lublin	K	E	V	H	M	E	R	N	F	S	A	A	V	I	K	S	A	S	L	L
A/domestic cat/Poland/H247/2023 H5N1 2023–06–20 Gdansk	K	E	V	H	**I**	**D**	R	N	F	S	A	A	V	I	K	S	A	S	L	L
A/domestic cat/Poland/H253/2023 H5N1 2023–06–22 Lublin	K	E	V	H	**I**	**D**	R	N	F	S	A	A	**A**	I	K	S	A	S	L	L

Letters in bold indicate differences between Polish viruses.

### Molecular markers of virus adaptation in mammals

Amino acid differences identified in the viral proteins of the analysed viruses are detailed in Table 2. In particular, all the viruses possess mutation PB2-E627K, which is an important molecular marker of virus adaptation to mammals [13-23]. The same mutation was present in the H5N1 virus detected in the white stork at the beginning of June (A/white_stork/Poland/MB244/2023). This mutation has rarely been observed in H5N1 viruses collected from birds during the ongoing epidemic wave (0.16% of the viruses from birds) but has frequently been acquired by the virus after transmission to mammals (17% of viruses from mammals) [1]. Moreover, all the viruses from cats possessed mutation PB2-K526R, which is another marker of mammalian adaptation. Of note, the Polish H5N1 viruses from cats gained dual 526R/627K substitutions in the PB2 protein and are the only ones characterised during the 2.3.4.4b world-wide current epidemic wave showing both mutations.

## Discussion

We have recently witnessed changes in the course of the HPAI epidemic, i.e. the spread to the Americas, the persistence of the virus all year round, a greater range of avian hosts, as well as the number of mammals infected. The above facts demonstrate worrying changes in the biological properties of circulating HPAI H5N1 viruses, which confer to the viruses the ability to infect a greater number of wild bird species that are not normally susceptible, expanding the reservoir of infection for poultry [24]. Longer and more efficient seeding of the virus has also been observed in some birds, potentially affecting the virus' greater ability to survive during the summer. Consequently, the amount and pressure of the virus in the environment increases, which in turn increases the risk of its introduction into the poultry population, but also the spread to other animal species – mammals in particular. 

We describe the detection of HPAI A(H5N1) virus infections in 25 cats during the second half of June in six voivodeships in Poland. Complete genome sequences of 19 HPAI H5N1 virus-positive cats indicate that the viruses belonged to clade 2.3.4.4b, genotype CH (H5N1-A/Eurasian_Wigeon/Netherlands/3/2022-like). The cat viruses were highly related to each other and clustered with a virus of the same genotype detected at the beginning of June in a white stork in Poland. When infections appeared in the cats, it was the BB genotype that was initially suspected as the cause of infection, considering that the EURL had issued a warning stating that the infection in poultry with this genotype could go undetected [1]. In April 2023, it was reported that the infection with the BB genotype could give anomalous disease signs in some poultry species such as turkeys or commercial layers, characterised by low mortality, very low prevalence of infection and almost the total absence of the typical HPAI signs, i.e. egg drop or reduced feed consumption. When there was a definite dominance of this genotype over others detected in wild birds, the veterinary inspectorate and poultry veterinarians in Poland received this information requesting to monitor the health status of poultry flocks very closely.

One of the possible sources of infection in cats seems to have been the consumption of poultry meat. However, from the interviews with cat owners, this is not so clear, as some cats received only specific food and had no contact with the outside environment. On the other hand, we also detected the infection in a stray cat (although it cannot be ruled out that someone had fed it with leftover raw chicken meat). Furthermore, the detection of the CH genotype virus in cats, which had been present in poultry almost 5 months earlier, further obscures the situation. Of course, there are known situations of silent virus infections in commercial poultry, as recently described in broilers in Italy [25], but the BB genotype is now circulating among wild birds and it is this genotype that has been the most suspected in terms of feline infections. However, the occurrence of the CH genotype in poultry would not necessarily need to coincide with its occurrence in cats because freezing of poultry meat is common in the preparation or storage of cat and other pet food. And, as discussed earlier, the surveillance system for influenza infections in poultry may not have detected single outbreaks caused by the CH genotype. It should also be remembered that Poland also imports poultry meat from other countries where the supervision system may be imperfect.

Cats found to be infected with the HPAI H5N1 virus suffered from severe outcome of the disease and showed respiratory and nervous symptoms including death, and large amounts of virus genome were detected especially in the brain, but also in the lungs and bronchi. This is similar to recent reports on pathogenicity of A(H5N1) 2.3.4.4b virus lineage in ferrets which targeted the central nervous system causing dramatic neurologic involvement [26].

All the viruses from cats possess two mutations in the PB2 protein, E627K and K526R, which are molecular markers of virus adaptation to mammals [13]. The PB2-E627K mutation has been demonstrated to enhance polymerase activity, virus replication, transmission and, in certain cases, pathogenicity and mortality in mammals [14-23]. The PB2-K526R mutation has in some avian influenza viruses been responsible for human cases (H5N1 and H7N9) and in the majority of the seasonal influenza A(H3N2) viruses [27]. A previous study showed that influenza A(H7N9) viruses possessing both 526R and 627K replicate more efficiently in mammalian (but not avian) cells and in mouse lung tissues, and cause greater mortality in infected mice [28]. Interestingly, the PB2-E627K mutation was present in the influenza A(H5N1) virus detected in the white stork at the beginning of June. The white stork is a carnivorous bird that feeds on a great variety of food: insects, earthworms, reptiles, amphibians and small mammals. Studies carried out on the population of white storks in Poland have shown that the diet of birds coming to breed was dominated by carp (*Cyprinus carpio*), European moles (*Talpa europaea*), voles (*Microtus arvalis* and *agrestis*) and earthworms (*Lumbricidae*), together accounting for 49.9% of the biomass eaten by them. Amphibians accounted for only 4.9% of the biomass they ate [29]. On one hand, it cannot be ruled out that the white stork became infected with the HPAI H5N1 virus containing a mammalian adaptation mutation through the ingestion of an infected mammal. It is known that small mammals such as rodents can serve as mechanical vectors or active shedders of avian influenza viruses [30]. On the other hand, infection of bank voles with the H5 and H7 subtypes of HPAI virus did not cause any symptoms of disease, but reversely resulted in shedding of high amounts of the virus [31,32]. The second mammal-adapted PB2-K526R mutation may have arisen as a result of infection in the cat's body. Nevertheless, it cannot be ruled out that the viruses with both mutations have been silently circulating in the bird population in Poland. To date, the Polish influenza A(H5N1) viruses from cats are the only ones characterised during the current epidemic wave showing both mutations. Additionally, the viruses from the Polish cats have shown 0–12 nucleotide and 0–8 amino acid differences distributed along the entire genome (see Supplementary Table S5 for nucleotide differences). The number of observed mutations suggests that the cats may have been exposed to multiple sources of infection of highly related viruses. However, we cannot completely exclude that mutations may have been acquired during the intra-host evolution of the virus in each animal.

The urgent question at the moment is to identify the direct source of virus infection in cats. The simultaneous detection of highly similar viruses over a vast geographical area undermines the hypothesis of direct transmission from wild birds to cats and points in the direction of an unidentified intermediate food source, e.g. poultry meat contaminated with the virus, that had accidentally entered the cats’ food chain. This pathway of transmission requires careful investigation that is currently underway. 

In view of the high number of infections in cats in Poland and also in view of recent announcements by international institutions (World Health Organization (WHO), World Organisation for Animal Health (WOAH) and Food and Agriculture Organization (FOA)) as well as European authorities (European Centre for Disease Prevention and Control (ECDC), European Food Safety Authority (EFSA) and EURL) that outbreaks of avian influenza in animals pose a threat to humans [33,34], the veterinary inspection in Poland has issued recommendations for cat owners to restrict the outdoor access for animals, stop feeding them with raw poultry meat, and to disinfect surfaces potentially in contact with the bird environment (e.g. shoe soles, terrace surfaces). The occurrence of such outbreaks in cats in other European countries cannot be ruled out either. If the source of infection was meat from a Polish poultry farm, it could potentially be exported to other countries, but there have been no reports on similar events outside of Poland to date.

## Conclusions

Recently, there have been a number of worrying changes in the ongoing HPAI outbreak. Another such unusual situation has occurred in Poland – the unprecedented scale of HPAI H5N1 virus infections of cats. Although the most likely source appears to be poultry meat, no such meat has been identified to date. Surveillance of poultry should certainly be enhanced, but also for certain, susceptible species of farmed mammals kept close to infected poultry farms. In addition, it seems reasonable to carry out scientific research into the susceptibility to influenza of other animals, in particular small mammals such as moles or voles. Furthermore, this study highlights the need in Europe to include *Mammalia* in the group of species posing a considerable risk for the spread of HPAI, in order to provide health authorities with tools and guidelines for the proper management of such cases.

## Supplementary Data

2851000 Supplement
