# Authors’ correction for Euro Surveill. 2023;28(31)

**DOI:** 10.2807/1560-7917.ES.2024.29.10.240307c

**Published:** 2024-03-07

**Authors:** Eurosurveillance editorial team

**Affiliations:** 1 European Centre for Disease Prevention and Control (ECDC), Stockholm, Sweden

In the article entitled ‘Outbreak of highly pathogenic avian influenza A(H5N1) clade 2.3.4.4b virus in cats, Poland, June to July 2023’ by Domańska-Blicharz et al. published on 3 August 2023, the name of co-author Bianca Zecchin was misspelled as Bianca Zechchin in the originally published version. This was corrected on 6 March 2024 at the request of the authors.

