# Supplementary material for: Outbreak of highly pathogenic avian influenza A(H5N1) clade 2.3.4.4b virus in cats, Poland, June to July 2023
Source: Euro Surveill. 2023 Aug 3;28(31):2300366. doi: 10.2807/1560-7917.ES.2023.28.31.2300366 (PMC10401911; doi:10.2807/1560-7917.ES.2023.28.31.2300366)
Supplement: Supplement [file 23-00366_DOMANSKA_Supplement_2aug.pdf]

Supplementary materials

This supplementary material is hosted by Eurosurveillance as supporting information alongside the article "Outbreak of Highly Pathogenic Avian Influenza A(H5N1) Clade 2.3.4.4b Virus in Cats in Poland", on behalf of the authors, who remain responsible for the accuracy and appropriateness of the content. The same standards for ethics, copyright, attributions and permissions as for the article apply. Supplements are not edited by Eurosurveillance and the journal is not responsible for the maintenance of any links or email addresses provided therein.

CONTENTS

S1. List of strains identified in birds (wild and poultry) submitted to the GISAID database.
S2. Photo's of diseased/necropsied cats.
S3. Detailed description of post-mortem lesions observed during examination of 11.
S4. Maximum likelihood phylogenetic trees of all segments of highly pathogenic avian influenza H5N1 viruses from cats (n = 19) and avian species (n = 84) in Poland as well as H5 sequences collected from different European countries (n = 161)[a].

- polymerase basic 2 protein gene - PB2
- polymerase basic 1 protein gene - PB1
- polymerase acidic protein gene - PA
- haemagglutinin gene – HA
- nucleoprotein – NP
- neuraminidase gene - NA
- matrix protein gene - MP
- non-structural protein gene – NS

[a] list of the sequences used for above analyses

S5. Nucleotide differences among the sequences of the H5N1 viruses collected from the nineteen cats in Poland.

S1. List of strains identified in birds (wild and poultry) submitted to the GISAID database

No	Isolate	Collection date	GISAID Isolate ID	Genotype
1	A/turkey/Poland/H427/2022(H5N1)	14-12-2022	EPI_ISL_18033134	CH
2	A/domestic_duck/Poland/H429/2022(H5N1)	15-12-2022	EPI_ISL_18032446	CH
3	A/domestic_duck/Poland/H433/2022(H5N1)	16-12-2022	EPI_ISL_18033137	CH
4	A/domestic_goose/Poland/H440/2022(H5N1)	17-12-2022	EPI_ISL_18032447	CH
5	A/domestic_duck/Poland/H439/2022(H5N1)	19-12-2022	EPI_ISL_18033139	CH
6	A/laying_hen/Poland/H443/2022(H5N1)	19-12-2022	EPI_ISL_18033140	CH
7	A/domestic_duck/Poland/H452-T/22(H5N1)	22-12-2022	EPI_ISL_18033152	CH
8	A/domestic_duck/Poland/H473-T/22(H5N1)	22-12-2022	EPI_ISL_18033157	CH
9	A/domestic_duck/Poland/H447/2022(H5N1)	22-12-2022	EPI_ISL_18033142	CH
10	A/domestic_goose/Poland/H458-T/22(H5N1)	23-12-2022	EPI_ISL_18033156	CH
11	A/mute_swan/Poland/MB268-TKL/2022(H5N1)	25-12-2022	EPI_ISL_18033161	CH
12	A/turkey/Poland/H463-T/22(H5N1)	26-12-2022	EPI_ISL_18033154	CH
13	A/domestic_duck/Poland/H460-T/22(H5N1)	26-12-2022	EPI_ISL_18033149	CH
14	A/domestic_duck/Poland/H470-K2T/22(H5N1)	27-12-2022	EPI_ISL_18033153	CH
15	A/buzzard/Poland/MB275-TKL/2022(H5N1)	28-12-2022	EPI_ISL_18033160	CH
16	A/domestic_duck/Poland/H487-T/22(H5N1)	28-12-2022	EPI_ISL_18033404	CH
17	A/domestic_duck/Poland/H477-T/22(H5N1)	28-12-2022	EPI_ISL_18033405	CH
18	A/domestic_duck/Poland/H482-T/22(H5N1)	29-12-2022	EPI_ISL_18033151	CH
19	A/domestic_duck/Poland/H485-N/22(H5N1)	29-12-2022	EPI_ISL_18033402	CH
20	A/domestic_duck/Poland/H09-T/23(H5N1)	30-12-2022	EPI_ISL_18033150	CH
21	A/turkey/Poland/H03-T/2023(H5N1)	2-1-2023	EPI_ISL_18033155	CH
22	A/turkey/Poland/H23-K2T/23(H5N1)	4-1-2023	EPI_ISL_18033399	CH
23	A/mute_swan/Poland/MB007-M1/23(H5N1)	4-1-2023	EPI_ISL_18033162	CH
24	A/mute_swan/Poland/MB012-M1/23(H5N1)	5-1-2023	EPI_ISL_18033163	CH
25	A/turkey/Poland/H26-T/23(H5N1)	7-1-2023	EPI_ISL_18033403	CH
26	A/laying_hen/Poland/H30-T/23(H5N1)	8-1-2023	EPI_ISL_18033401	CH

27	A/domestic_duck/Poland/H31-T/23(H5N1)	9-1-2023	EPI_ISL_18033400	CH
28	A/mute_swan/Poland/MB015/2023(H5N1)	10-1-2023	EPI_ISL_18032171	CH
29	A/mute_swan/Poland/MB014/2023(H5N1)	10-1-2023	EPI_ISL_18032166	CH
30	A/domestic_duck/Poland/H75-K1T1/2023(H5N1)	16-1-2023	EPI_ISL_18032444	CH
31	A/domestic_duck/Poland/H73-T1/2023(H5N1)	16-1-2023	EPI_ISL_18033168	CH
32	A/domestic_duck/Poland/H74-T1/2023(H5N1)	16-1-2023	EPI_ISL_18033170	CH
33	A/domestic_duck/Poland/H70-T1/2023(H5N1)	16-1-2023	EPI_ISL_18033169	CH
34	A/domestic_duck/Poland/H72-T1/2023(H5N1)	16-1-2023	EPI_ISL_18032204	CH
35	A/mute_swan/Poland/MB029/2023(H5N1)	16-1-2023	EPI_ISL_18033167	CH
36	A/domestic_duck/Poland/H77-T1/2023(H5N1)	17-1-2023	EPI_ISL_18033171	CH
37	A/domestic_duck/Poland/H78-K2T1/2023(H5N1)	17-1-2023	EPI_ISL_18033172	CH
38	A/domestic_duck/Poland/H84-T1/2023(H5N1)	18-1-2023	EPI_ISL_18033174	CH
39	A/turkey/Poland/H82-T1/2023(H5N1)	18-1-2023	EPI_ISL_18033173	CH
40	A/domestic_duck/Poland/H86-T1/2023(H5N1)	18-1-2023	EPI_ISL_18033175	CH
41	A/domestic_duck/Poland/H93-T1/2023(H5N1)	19-1-2023	EPI_ISL_18033406	CH
42	A/domestic_duck/Poland/H89-T1/2023(H5N1)	20-1-2023	EPI_ISL_18033407	CH
43	A/turkey/Poland/H90-K4T4/2023(H5N1)	21-1-2023	EPI_ISL_18033176	CH
44	A/raven/Poland/MB035/2023(H5N1)	22-1-2023	EPI_ISL_18033177	CH
45	A/domestic_duck/Poland/H96-T6/2023(H5N1)	23-1-2023	EPI_ISL_18033178	CH
46	A/mute_swan/Poland/MB037/2023(H5N1)	23-1-2023	EPI_ISL_18033179	CH
47	A/mute_swan/Poland/MB033/2023(H5N1)	24-1-2023	EPI_ISL_18033180	CH
48	A/mute_swan/Poland/MB113/2023(H5N1)	10-2-2023	EPI_ISL_18033192	CH
49	A/mute_swan/Poland/MB095-L1M/2023(H5N1)	14-2-2023	EPI_ISL_18032445	CH
50	A/chicken/Poland/H130-T/2023(H5N1)	20-2-2023	EPI_ISL_18033197	CH
51	A/mute_swan/Poland/MB107/2023(H5N1)	23-2-2023	EPI_ISL_18033198	CH
52	A/white_stork/Poland/MB244/2023(H5N1)	4-6-2023	EPI_ISL_17978700	CH
53	A/black-headed_gull/Poland/MB081/2023(H5N1)	7-2-2023	EPI_ISL_18033185	BB
54	A/black-headed_gull/Poland/MB121-M/2023(H5N1)	13-3-2023	EPI_ISL_18033200	BB
55	A/black-headed_gull/Poland/MB129-NJ/2023(H5N1)	22-3-2023	EPI_ISL_18033203	BB

56	A/black-headed_gull/Poland/MB131-J/2023(H5N1)	23-3-2023	EPI_ISL_18033204	BB
57	A/black-headed_gull/Poland/MB141-T/2023(H5N1)	20-4-2023	EPI_ISL_18033205	BB
58	A/black-headed_gull/Poland/MB209-NJ/2023(H5N1)	1-5-2023	EPI_ISL_18033232	BB
59	A/black-headed_gull/Poland/MB166/2023(H5N1)	5-5-2023	EPI_ISL_18033219	BB
60	A/black-headed_gull/Poland/MB168-M2/2023(H5N1)	5-5-2023	EPI_ISL_18033221	BB
61	A/black-headed_gull/Poland/MB176-M6/2023(H5N1)	5-5-2023	EPI_ISL_18033222	BB
62	A/Mediterranean_gull/Poland/MB204/2023(H5N1)	8-5-2023	EPI_ISL_18033231	BB
63	A/black-headed_gull/Poland/MB180-NMJ/2023(H5N1)	9-5-2023	EPI_ISL_18033223	BB
64	A/common_tern/Poland/MB182-NJ/2023(H5N1)	10-5-2023	EPI_ISL_18033226	BB
65	A/black-headed_gull/Poland/MB185-NJ/2023(H5N1)	10-5-2023	EPI_ISL_18033227	BB
66	A/black-headed_gull/Poland/MB198-NJ/2023(H5N1)	11-5-2023	EPI_ISL_18033229	BB
67	A/mute_swan/Poland/MB203-NJ/2023(H5N1)	11-5-2023	EPI_ISL_18033230	BB
68	A/black-headed_gull/Poland/MB211/2023(H5N1)	14-5-2023	EPI_ISL_18033234	BB
69	A/black-headed_gull/Poland/MB210/2023(H5N1)	16-5-2023	EPI_ISL_18033233	BB
70	A/mute_swan/Poland/MB045/2023(H5N1)	31-1-2023	EPI_ISL_18033181	AB
71	A/domestic_duck/Poland/H101-T2/2023(H5N1)	3-2-2023	EPI_ISL_18033182	AB
72	A/turkey/Poland/H103-T4/2023(H5N1)	3-2-2023	EPI_ISL_18033183	AB
73	A/turkey/Poland/H105-T4/2023(H5N1)	6-2-2023	EPI_ISL_18033184	AB
74	A/domestic_duck/Poland/H108-K3T1/2023(H5N1)	8-2-2023	EPI_ISL_18033186	AB
75	A/turkey/Poland/H110-T/2023(H5N1)	9-2-2023	EPI_ISL_18033187	AB
76	A/turkey/Poland/H111-T3/2023(H5N1)	10-2-2023	EPI_ISL_18033188	AB
77	A/turkey/Poland/H115-T/2023(H5N1)	10-2-2023	EPI_ISL_18033189	AB
78	A/turkey/Poland/H116-T/2023(H5N1)	10-2-2023	EPI_ISL_18033190	AB
79	A/turkey/Poland/H117-T2/2023(H5N1)	12-2-2023	EPI_ISL_18033193	AB
80	A/domestic_duck/Poland/H118-T4/2023(H5N1)	12-2-2023	EPI_ISL_18033194	AB
81	A/laying_hen/Poland/H126-KL2/2023(H5N1)	15-2-2023	EPI_ISL_18033195	AB
82	A/mallard/Poland/MB105/2023(H5N1)	1-3-2023	EPI_ISL_18033199	AB
83	A/domestic_duck/Poland/H148-T1/2023(H5N1)	21-3-2023	EPI_ISL_18033201	AB
84	A/domestic_duck/Poland/H149-NM/2023(H5N1)	21-3-2023	EPI_ISL_18033202	AB

S2. Photo’s of diseased/necropsied cats.

Constricted pupils of a cat unresponsive to light *(by Ł. Adaszek)*.

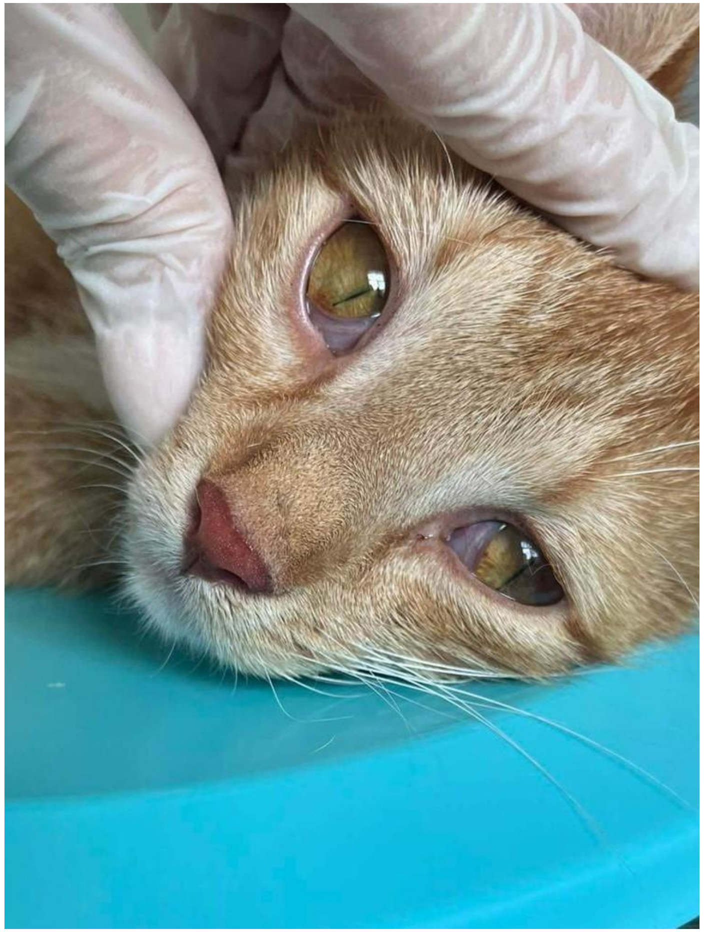

S3. Detailed description of post-mortem lesions observed during examination of 11.

Post-mortem examination in 11 cats revealed the presence of lesions in every organ showed the presence of lesions in each organ, which were congested, sometimes swollen with the presence of bloody fluid (a detailed description of the postmortem lesions is included in the supplementary material).: red nasal cavity mucosa, covered with bloody fluid; presence of serum admixed with blood, thick, turbid fluid in the respiratory part of the pharynx (an example below); laryngeal and tracheal mucosa creamy red, locally dark red, with features of congestion. In addition, the presence of bloody, foamy, sometimes turbid and thick fluid was observed in the trachea; the lungs showed features of congestion, inflammation, shock, alveolar haemorrhages, with lighter foci of emphysema and darker foci of atelectasis, and had a cushion-like consistency; bloody fluid was observed in the pericardial sac, the spleen was enlarged, the pancreas was pink and at times locally red. Further, we noted the presence of white diffuse lesions with congestive features, kidneys with features of congestion, oedema and shock, with heterogeneous, uneven surface, clearly marked superficial vessels; the liver was brown, locally light brown or dark pink, with firm consistency, locally friable, with features of congestion, clearly marked lobular structure, presence of scattered interstitial lesions of infiltrative or hypertrophic character; the wall of the small and large intestine was locally thickened, the mucosa creamy-green; bloody fluid was present in the cranial cavity and subdurally; the superficial vessels of the brain were congested; brain tissue was in a partially decomposed state.

Recurrent mucous , clumped "pus" in the trachea that significantly impeded cats breathing *(by A. Świątalska)*.

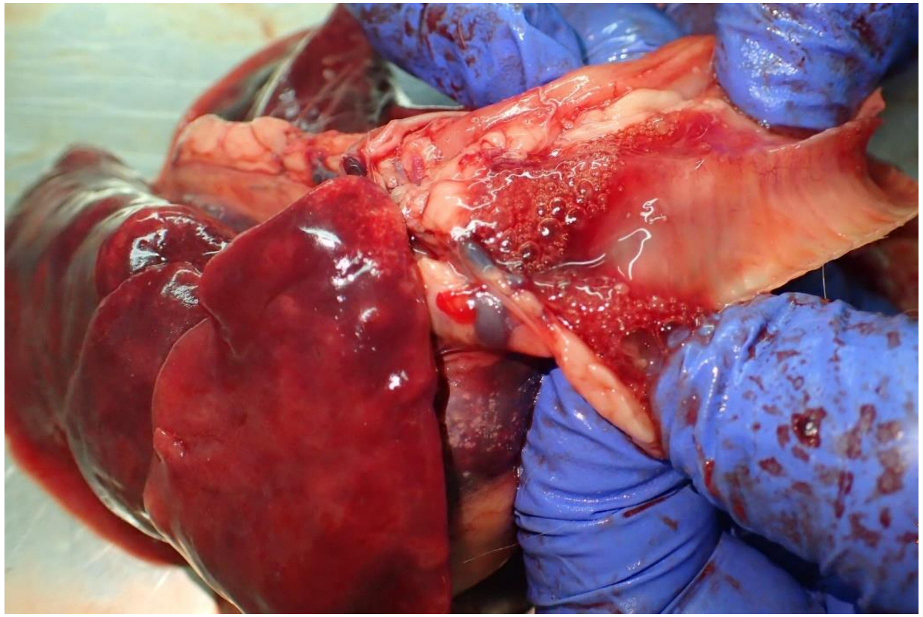

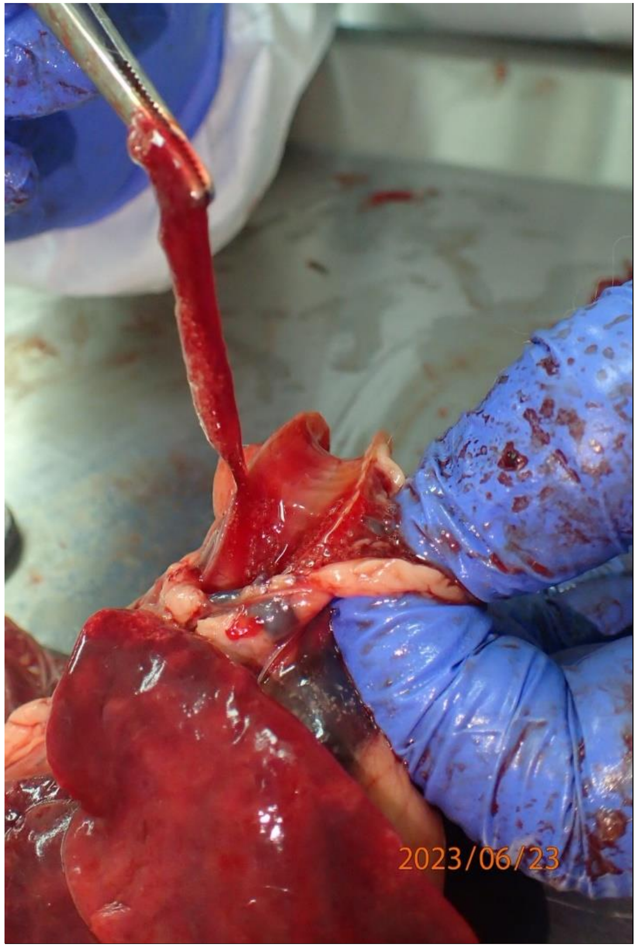

S4. Maximum likelihood phylogenetic trees of all segments of highly pathogenic avian influenza H5N1 viruses from cats (n = 19) and avian species (n = 84) in Poland as well as H5 sequences collected from different European countries (n = 161)[a]

(cat sequences in red, bird sequences identified in Poland - blue)

The subsequent pages show the phylogenetic trees for the following segments:

polymerase basic 2 protein gene - PB2
polymerase basic 1 protein gene - PB1
polymerase acidic protein gene - PA
haemagglutinin gene – HA
nucleoprotein gene – NP
neuraminidase gene - NA
matrix protein gene - MP
non-structural protein gene – NS

[a] list of the sequences used for the analyses. We gratefully acknowledge the authors, laboratories initiating and submitting sequences from the EpiFlu™ GISAID database on which this study is partly based.

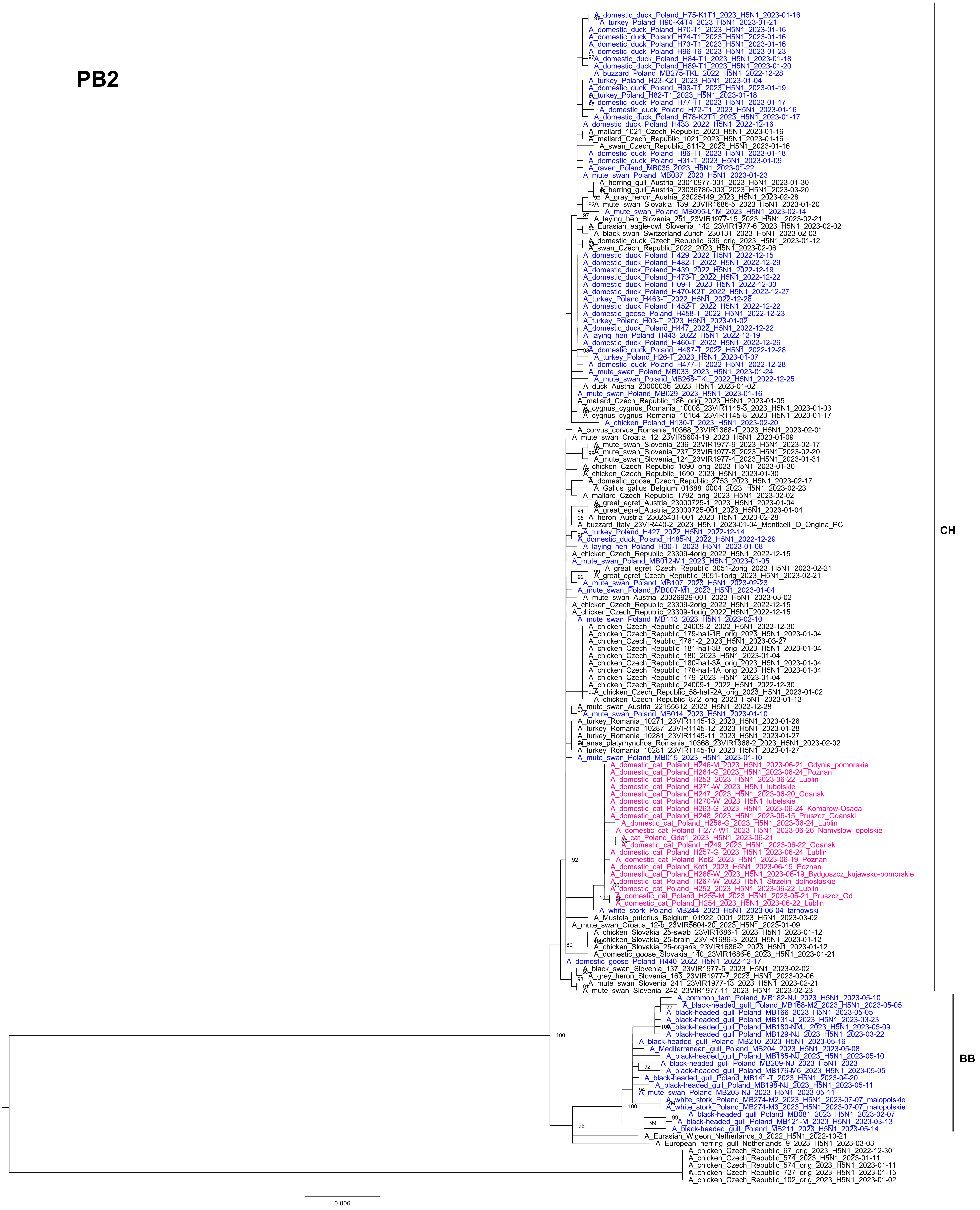
PB2
A_domestic_duck_Poland_H75-K1T1_2023_H5N1_2023-01-16
A_turkey_Poland_H90-K4T4_2023_H5N1_2023-01-21
A_domestic_duck_Poland_H70-T1_2023_H5N1_2023-01-16
A_domestic_duck_Poland_H74-T1_2023_H5N1_2023-01-16
A_domestic_duck_Poland_H73-T1_2023_H5N1_2023-01-16
A_domestic_duck_Poland_H96-T6_2023_H5N1_2023-01-23
A_domestic_duck_Poland_H84-T1_2023_H5N1_2023-01-18
A_domestic_duck_Poland_H89-T1_2023_H5N1_2023-01-20
A_buzzard_Poland_MB275-TKL_2022_H5N1_2022-12-28
A_turkey_Poland_H23-K2T_2023_H5N1_2023-01-04
A_domestic_duck_Poland_H93-T1_2023_H5N1_2023-01-19
A_turkey_Poland_H82-T1_2023_H5N1_2023-01-18
A_domestic_duck_Poland_H77-T1_2023_H5N1_2023-01-17
A_domestic_duck_Poland_H72-T1_2023_H5N1_2023-01-16
A_domestic_duck_Poland_H78-K2T1_2023_H5N1_2023-01-17
A_domestic_duck_Poland_H433_2022_H5N1_2022-12-16
A_mallard_1021_Czech_Republic_2023_H5N1_2023-01-16
A_mallard_Czech_Republic_1021_2023_H5N1_2023-01-16
A_swan_Czech_Republic_811-2_2023_H5N1_2023-01-16
A_domestic_duck_Poland_H86-T1_2023_H5N1_2023-01-18
A_domestic_duck_Poland_H31-T_2023_H5N1_2023-01-09
A_raven_Poland_MB035_2023_H5N1_2023-01-22
A_mute_swan_Poland_MB037_2023_H5N1_2023-01-23
A_herring_gull_Austria_23010977-001_2023_H5N1_2023-01-30
A_herring_gull_Austria_23036780-003_2023_H5N1_2023-03-20
A_gray_heron_Austria_23025449_2023_H5N1_2023-02-28
A_mute_swan_Slovakia_139_23VIR1686-5_2023_H5N1_2023-01-20
A_mute_swan_Poland_MB095-L1M_2023_H5N1_2023-02-14
A_laying_hen_Slovenia_251_23VIR1977-15_2023_H5N1_2023-02-21
A_Eurasian_eagle-owl_Slovenia_142_23VIR1977-6_2023_H5N1_2023-02-02
A_black-swan_Switzerland-Zurich_230131_2023_H5N1_2023-02-03
A_domestic_duck_Czech_Republic_636_orig_2023_H5N1_2023-01-12
A_swan_Czech_Republic_2022_2023_H5N1_2023-02-06
A_domestic_duck_Poland_H429_2022_H5N1_2022-12-15
A_domestic_duck_Poland_H482-T_2022_H5N1_2022-12-29
A_domestic_duck_Poland_H439_2022_H5N1_2022-12-19
A_domestic_duck_Poland_H473-T_2022_H5N1_2022-12-22
A_domestic_duck_Poland_H09-T_2023_H5N1_2022-12-30
A_domestic_duck_Poland_H470-K2T_2022_H5N1_2022-12-27
A_turkey_Poland_H463-T_2022_H5N1_2022-12-26
A_domestic_duck_Poland_H452-T_2022_H5N1_2022-12-22
A_domestic_goose_Poland_H458-T_2022_H5N1_2022-12-23
A_turkey_Poland_H03-T_2023_H5N1_2023-01-02
A_domestic_duck_Poland_H447_2022_H5N1_2022-12-22
A_laying_hen_Poland_H443_2022_H5N1_2022-12-19
A_domestic_duck_Poland_H460-T_2022_H5N1_2022-12-26
A_domestic_duck_Poland_H487-T_2022_H5N1_2022-12-28
A_turkey_Poland_H26-T_2023_H5N1_2023-01-07
A_domestic_duck_Poland_H477-T_2022_H5N1_2022-12-28
A_mute_swan_Poland_MB033_2023_H5N1_2023-01-24
A_mute_swan_Poland_MB268-TKL_2022_H5N1_2022-12-25
A_duck_Austria_23000036_2023_H5N1_2023-01-02
A_mute_swan_Poland_MB029_2023_H5N1_2023-01-16
A_mallard_Czech_Republic_186_orig_2023_H5N1_2023-01-05
A_cygnus_cygnus_Romania_10008_23VIR1145-3_2023_H5N1_2023-01-03
A_cygnus_cygnus_Romania_10164_23VIR1145-8_2023_H5N1_2023-01-17
A_chicken_Poland_H130-T_2023_H5N1_2023-02-20
A_corvus_corvus_Romania_10368_23VIR1368-1_2023_H5N1_2023-02-01
A_mute_swan_Croatia_12_23VIR5604-19_2023_H5N1_2023-01-09
A_mute_swan_Slovenia_236_23VIR1977-9_2023_H5N1_2023-02-17
A_mute_swan_Slovenia_237_23VIR1977-8_2023_H5N1_2023-02-20
A_mute_swan_Slovenia_124_23VIR1977-4_2023_H5N1_2023-01-31
A_chicken_Czech_Republic_1690_orig_2023_H5N1_2023-01-30
A_chicken_Czech_Republic_1690_2023_H5N1_2023-01-30
A_domestic_goose_Czech_Republic_2753_2023_H5N1_2023-02-17
A_Gallus_gallus_Belgium_01688_0004_2023_H5N1_2023-02-23
A_mallard_Czech_Republic_1792_orig_2023_H5N1_2023-02-02
A_great_egret_Austria_23000725-1_2023_H5N1_2023-01-04
A_great_egret_Austria_23000725-001_2023_H5N1_2023-01-04
A_heron_Austria_23025431-001_2023_H5N1_2023-02-28
A_buzzard_Italy_23VIR440-2_2023_H5N1_2023-01-04_Monticelli_D_Ongina_PC
A_turkey_Poland_H427_2022_H5N1_2022-12-14
A_domestic_duck_Poland_H485-N_2022_H5N1_2022-12-29
A_laying_hen_Poland_H30-T_2023_H5N1_2023-01-08
A_chicken_Czech_Republic_23309-4orig_2022_H5N1_2022-12-15
A_mute_swan_Poland_MB012-M1_2023_H5N1_2023-01-05
A_great_egret_Czech_Republic_3051-2orig_2023_H5N1_2023-02-21
A_great_egret_Czech_Republic_3051-1orig_2023_H5N1_2023-02-21
A_mute_swan_Poland_MB107_2023_H5N1_2023-02-23
A_mute_swan_Poland_MB007-M1_2023_H5N1_2023-01-04
A_mute_swan_Austria_23026929-001_2023_H5N1_2023-03-02
A_chicken_Czech_Republic_23309-2orig_2022_H5N1_2022-12-15
A_chicken_Czech_Republic_23309-1orig_2022_H5N1_2022-12-15
A_mute_swan_Poland_MB113_2023_H5N1_2023-02-10
A_chicken_Czech_Republic_24009-2_2022_H5N1_2022-12-30
A_chicken_Czech_Republic_179-hall-1B_orig_2023_H5N1_2023-01-04
A_chicken_Czech_Reublic_4761-2_2023_H5N1_2023-03-27
A_chicken_Czech_Republic_181-hall-3B_orig_2023_H5N1_2023-01-04
A_chicken_Czech_Republic_180_2023_H5N1_2023-01-04
A_chicken_Czech_Republic_180-hall-3A_orig_2023_H5N1_2023-01-04
A_chicken_Czech_Republic_178-hall-1A_orig_2023_H5N1_2023-01-04
A_chicken_Czech_Republic_179_2023_H5N1_2023-01-04
A_chicken_Czech_Republic_24009-1_2022_H5N1_2022-12-30
A_chicken_Czech_Republic_58-hall-2A_orig_2023_H5N1_2023-01-02
A_chicken_Czech_Republic_872_orig_2023_H5N1_2023-01-13
A_mute_swan_Austria_22155612_2022_H5N1_2022-12-28
A_mute_swan_Poland_MB014_2023_H5N1_2023-01-10
A_turkey_Romania_10271_23VIR1145-13_2023_H5N1_2023-01-26
A_turkey_Romania_10287_23VIR1145-12_2023_H5N1_2023-01-28
A_turkey_Romania_10281_23VIR1145-11_2023_H5N1_2023-01-27
A_anas_platyrhynchos_Romania_10368_23VIR1368-2_2023_H5N1_2023-02-02
A_turkey_Romania_10281_23VIR1145-10_2023_H5N1_2023-01-27
A_mute_swan_Poland_MB015_2023_H5N1_2023-01-10
A_domestic_cat_Poland_H246-M_2023_H5N1_2023-06-21_Gdynia_pomorskie
A_domestic_cat_Poland_H264-G_2023_H5N1_2023-06-24_Poznan
A_domestic_cat_Poland_H253_2023_H5N1_2023-06-22_Lublin
A_domestic_cat_Poland_H271-W_2023_H5N1_lubelskie
A_domestic_cat_Poland_H247_2023_H5N1_2023-06-20_Gdansk
A_domestic_cat_Poland_H270-W_2023_H5N1_lubelskie
A_domestic_cat_Poland_H263-G_2023_H5N1_2023-06-24_Komarow-Osada
A_domestic_cat_Poland_H248_2023_H5N1_2023-06-15_Pruszcz_Gdanski
A_domestic_cat_Poland_H256-G_2023_H5N1_2023-06-24_Lublin
A_domestic_cat_Poland_H277-W1_2023_H5N1_2023-06-26_Namyslow_opolskie
A_cat_Poland_Gda1_2023_H5N1_2023-06-21
A_domestic_cat_Poland_H249_2023_H5N1_2023-06-22_Gdansk
A_domestic_cat_Poland_H257-G_2023_H5N1_2023-06-24_Lublin
A_domestic_cat_Poland_Kot2_2023_H5N1_2023-06-19_Poznan
A_domestic_cat_Poland_Kot1_2023_H5N1_2023-06-19_Poznan
A_domestic_cat_Poland_H266-W_2023_H5N1_2023-06-19_Bydgoszcz_kujawsko-pomorskie
A_domestic_cat_Poland_H267-W_2023_H5N1_Strzelin_dolnoslaskie
A_domestic_cat_Poland_H252_2023_H5N1_2023-06-22_Lublin
A_domestic_cat_Poland_H255-M_2023_H5N1_2023-06-21_Pruszcz_Gd
A_domestic_cat_Poland_H254_2023_H5N1_2023-06-22_Lublin
A_white_stork_Poland_MB244_2023_H5N1_2023-06-04_tarnowski
A_Mustela_putorius_Belgium_01922_0001_2023_H5N1_2023-03-02
A_mute_swan_Croatia_12-b_23VIR5604-20_2023_H5N1_2023-01-09
A_chicken_Slovakia_25-swab_23VIR1686-1_2023_H5N1_2023-01-12
A_chicken_Slovakia_25-brain_23VIR1686-3_2023_H5N1_2023-01-12
A_chicken_Slovakia_25-organs_23VIR1686-2_2023_H5N1_2023-01-12
A_domestic_goose_Slovakia_140_23VIR1686-6_2023_H5N1_2023-01-21
A_domestic_goose_Poland_H440_2022_H5N1_2022-12-17
A_black_swan_Slovenia_137_23VIR1977-5_2023_H5N1_2023-02-02
A_grey_heron_Slovenia_163_23VIR1977-7_2023_H5N1_2023-02-06
A_mute_swan_Slovenia_241_23VIR1977-13_2023_H5N1_2023-02-21
A_mute_swan_Slovenia_242_23VIR1977-11_2023_H5N1_2023-02-23
CH
A_common_tern_Poland_MB182-NJ_2023_H5N1_2023-05-10
A_black-headed_gull_Poland_MB168-M2_2023_H5N1_2023-05-05
A_black-headed_gull_Poland_MB166_2023_H5N1_2023-05-05
A_black-headed_gull_Poland_MB131-J_2023_H5N1_2023-03-23
A_black-headed_gull_Poland_MB180-NMJ_2023_H5N1_2023-05-09
A_black-headed_gull_Poland_MB129-NJ_2023_H5N1_2023-03-22
A_black-headed_gull_Poland_MB210_2023_H5N1_2023-05-16
A_Mediterranean_gull_Poland_MB204_2023_H5N1_2023-05-08
A_black-headed_gull_Poland_MB185-NJ_2023_H5N1_2023-05-10
A_black-headed_gull_Poland_MB209-NJ_2023_H5N1_2023
A_black-headed_gull_Poland_MB176-M6_2023_H5N1_2023-05-05
A_black-headed_gull_Poland_MB141-T_2023_H5N1_2023-04-20
A_black-headed_gull_Poland_MB198-NJ_2023_H5N1_2023-05-11
A_mute_swan_Poland_MB203-NJ_2023_H5N1_2023-05-11
A_white_stork_Poland_MB274-M2_2023_H5N1_2023-07-07_malopolskie
A_white_stork_Poland_MB274-M3_2023_H5N1_2023-07-07_malopolskie
A_black-headed_gull_Poland_MB081_2023_H5N1_2023-02-07
A_black-headed_gull_Poland_MB121-M_2023_H5N1_2023-03-13
A_black-headed_gull_Poland_MB211_2023_H5N1_2023-05-14
BB
A_Eurasian_Wigeon_Netherlands_3_2022_H5N1_2022-10-21
A_European_herring_gull_Netherlands_9_2023_H5N1_2023-03-03
A_chicken_Czech_Republic_67_orig_2023_H5N1_2022-12-30
A_chicken_Czech_Republic_574_2023_H5N1_2023-01-11
A_chicken_Czech_Republic_574_orig_2023_H5N1_2023-01-11
A_chicken_Czech_Republic_727_orig_2023_H5N1_2023-01-15
A_chicken_Czech_Republic_102_orig_2023_H5N1_2023-01-02
0.006

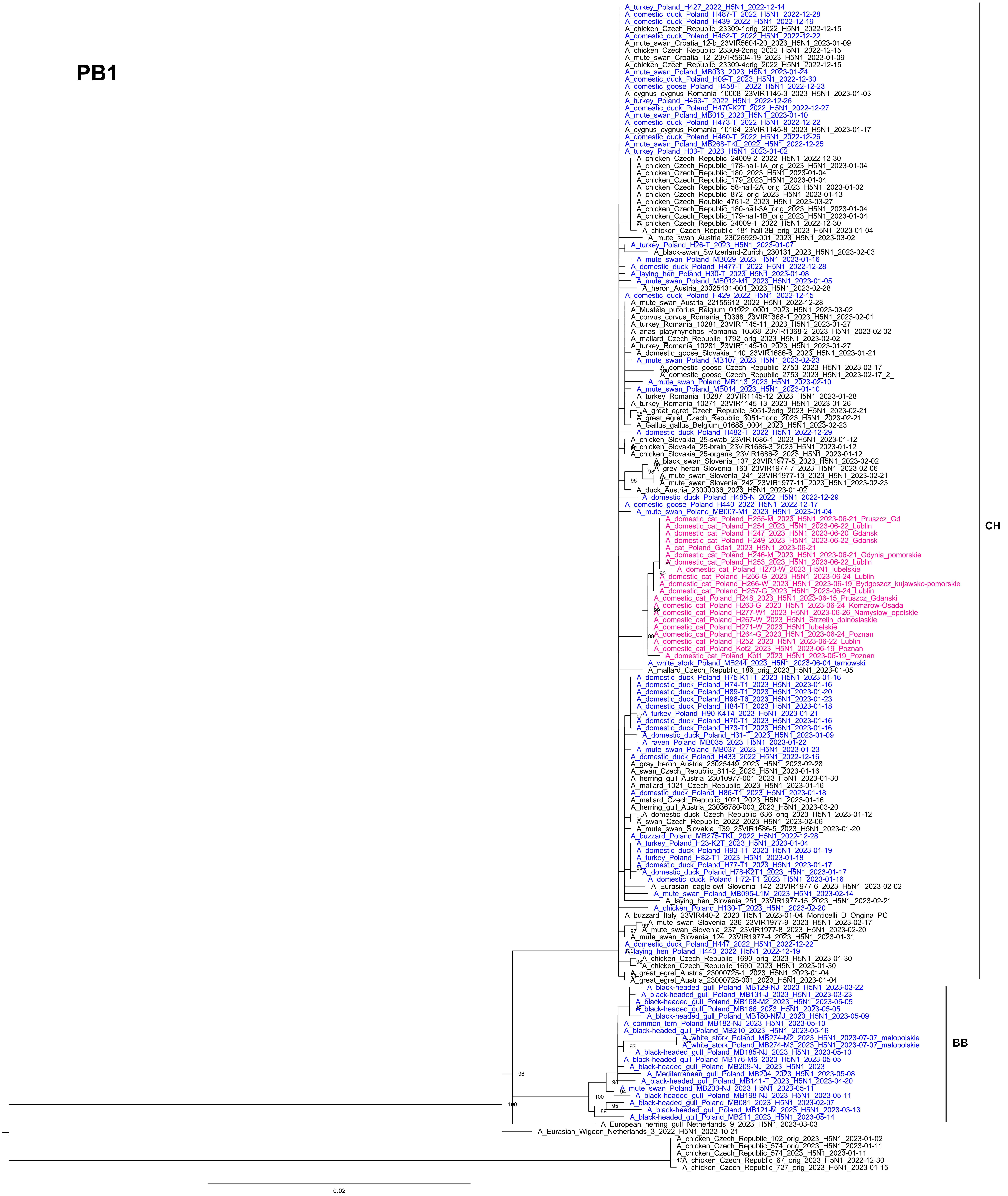
PB1
A_turkey_Poland_H427_2022_H5N1_2022-12-14
A_domestic_duck_Poland_H487-T_2022_H5N1_2022-12-28
A_domestic_duck_Poland_H439_2022_H5N1_2022-12-19
A_chicken_Czech_Republic_23309-1orig_2022_H5N1_2022-12-15
A_domestic_duck_Poland_H452-T_2022_H5N1_2022-12-22
A_mute_swan_Croatia_12-b_23VIR5604-20_2023_H5N1_2023-01-09
A_chicken_Czech_Republic_23309-2orig_2022_H5N1_2022-12-15
A_mute_swan_Croatia_12_23VIR5604-19_2023_H5N1_2023-01-09
A_chicken_Czech_Republic_23309-4orig_2022_H5N1_2022-12-15
A_mute_swan_Poland_MB033_2023_H5N1_2023-01-24
A_domestic_duck_Poland_H09-T_2023_H5N1_2022-12-30
A_domestic_goose_Poland_H458-T_2022_H5N1_2022-12-23
A_cygnus_cygnus_Romania_10008_23VIR1145-3_2023_H5N1_2023-01-03
A_turkey_Poland_H463-T_2022_H5N1_2022-12-26
A_domestic_duck_Poland_H470-K2T_2022_H5N1_2022-12-27
A_mute_swan_Poland_MB015_2023_H5N1_2023-01-10
A_domestic_duck_Poland_H473-T_2022_H5N1_2022-12-22
A_cygnus_cygnus_Romania_10164_23VIR1145-8_2023_H5N1_2023-01-17
A_domestic_duck_Poland_H460-T_2022_H5N1_2022-12-26
A_mute_swan_Poland_MB268-TKL_2022_H5N1_2022-12-25
A_turkey_Poland_H03-T_2023_H5N1_2023-01-02
A_chicken_Czech_Republic_24009-2_2022_H5N1_2022-12-30
A_chicken_Czech_Republic_178-hall-1A_orig_2023_H5N1_2023-01-04
A_chicken_Czech_Republic_180_2023_H5N1_2023-01-04
A_chicken_Czech_Republic_179_2023_H5N1_2023-01-04
A_chicken_Czech_Republic_58-hall-2A_orig_2023_H5N1_2023-01-02
A_chicken_Czech_Republic_872_orig_2023_H5N1_2023-01-13
A_chicken_Czech_Reublic_4761-2_2023_H5N1_2023-03-27
A_chicken_Czech_Republic_180-hall-3A_orig_2023_H5N1_2023-01-04
A_chicken_Czech_Republic_179-hall-1B_orig_2023_H5N1_2023-01-04
A_chicken_Czech_Republic_24009-1_2022_H5N1_2022-12-30
A_chicken_Czech_Republic_181-hall-3B_orig_2023_H5N1_2023-01-04
A_mute_swan_Austria_23026929-001_2023_H5N1_2023-03-02
A_turkey_Poland_H26-T_2023_H5N1_2023-01-07
A_black-swan_Switzerland-Zurich_230131_2023_H5N1_2023-02-03
A_mute_swan_Poland_MB029_2023_H5N1_2023-01-16
A_domestic_duck_Poland_H477-T_2022_H5N1_2022-12-28
A_laying_hen_Poland_H30-T_2023_H5N1_2023-01-08
A_mute_swan_Poland_MB012-M1_2023_H5N1_2023-01-05
A_heron_Austria_23025431-001_2023_H5N1_2023-02-28
A_domestic_duck_Poland_H429_2022_H5N1_2022-12-15
A_mute_swan_Austria_22155612_2022_H5N1_2022-12-28
A_Mustela_putorius_Belgium_01922_0001_2023_H5N1_2023-03-02
A_corvus_corvus_Romania_10368_23VIR1368-1_2023_H5N1_2023-02-01
A_turkey_Romania_10281_23VIR1145-11_2023_H5N1_2023-01-27
A_anas_platyrhynchos_Romania_10368_23VIR1368-2_2023_H5N1_2023-02-02
A_mallard_Czech_Republic_1792_orig_2023_H5N1_2023-02-02
A_turkey_Romania_10281_23VIR1145-10_2023_H5N1_2023-01-27
A_domestic_goose_Slovakia_140_23VIR1686-6_2023_H5N1_2023-01-21
A_mute_swan_Poland_MB107_2023_H5N1_2023-02-23
A_domestic_goose_Czech_Republic_2753_2023_H5N1_2023-02-17
A_domestic_goose_Czech_Republic_2753_2023_H5N1_2023-02-17_2_
A_mute_swan_Poland_MB113_2023_H5N1_2023-02-10
A_mute_swan_Poland_MB014_2023_H5N1_2023-01-10
A_turkey_Romania_10287_23VIR1145-12_2023_H5N1_2023-01-28
A_turkey_Romania_10271_23VIR1145-13_2023_H5N1_2023-01-26
A_great_egret_Czech_Republic_3051-2orig_2023_H5N1_2023-02-21
A_great_egret_Czech_Republic_3051-1orig_2023_H5N1_2023-02-21
A_Gallus_gallus_Belgium_01688_0004_2023_H5N1_2023-02-23
A_domestic_duck_Poland_H482-T_2022_H5N1_2022-12-29
A_chicken_Slovakia_25-swab_23VIR1686-1_2023_H5N1_2023-01-12
A_chicken_Slovakia_25-brain_23VIR1686-3_2023_H5N1_2023-01-12
A_chicken_Slovakia_25-organs_23VIR1686-2_2023_H5N1_2023-01-12
A_black_swan_Slovenia_137_23VIR1977-5_2023_H5N1_2023-02-02
A_grey_heron_Slovenia_163_23VIR1977-7_2023_H5N1_2023-02-06
A_mute_swan_Slovenia_241_23VIR1977-13_2023_H5N1_2023-02-21
A_mute_swan_Slovenia_242_23VIR1977-11_2023_H5N1_2023-02-23
A_duck_Austria_23000036_2023_H5N1_2023-01-02
A_domestic_duck_Poland_H485-N_2022_H5N1_2022-12-29
A_domestic_goose_Poland_H440_2022_H5N1_2022-12-17
A_mute_swan_Poland_MB007-M1_2023_H5N1_2023-01-04
A_domestic_cat_Poland_H255-M_2023_H5N1_2023-06-21_Pruszcz_Gd
A_domestic_cat_Poland_H254_2023_H5N1_2023-06-22_Lublin
A_domestic_cat_Poland_H247_2023_H5N1_2023-06-20_Gdansk
A_domestic_cat_Poland_H249_2023_H5N1_2023-06-22_Gdansk
A_cat_Poland_Gda1_2023_H5N1_2023-06-21
A_domestic_cat_Poland_H246-M_2023_H5N1_2023-06-21_Gdynia_pomorskie
A_domestic_cat_Poland_H253_2023_H5N1_2023-06-22_Lublin
A_domestic_cat_Poland_H270-W_2023_H5N1_lubelskie
A_domestic_cat_Poland_H256-G_2023_H5N1_2023-06-24_Lublin
A_domestic_cat_Poland_H266-W_2023_H5N1_2023-06-19_Bydgoszcz_kujawsko-pomorskie
A_domestic_cat_Poland_H257-G_2023_H5N1_2023-06-24_Lublin
A_domestic_cat_Poland_H248_2023_H5N1_2023-06-15_Pruszcz_Gdanski
A_domestic_cat_Poland_H263-G_2023_H5N1_2023-06-24_Komarow-Osada
A_domestic_cat_Poland_H277-W1_2023_H5N1_2023-06-26_Namyslow_opolskie
A_domestic_cat_Poland_H267-W_2023_H5N1_Strzelin_dolnoslaskie
A_domestic_cat_Poland_H271-W_2023_H5N1_lubelskie
A_domestic_cat_Poland_H264-G_2023_H5N1_2023-06-24_Poznan
A_domestic_cat_Poland_H252_2023_H5N1_2023-06-22_Lublin
A_domestic_cat_Poland_Kot2_2023_H5N1_2023-06-19_Poznan
A_domestic_cat_Poland_Kot1_2023_H5N1_2023-06-19_Poznan
A_white_stork_Poland_MB244_2023_H5N1_2023-06-04_tarnowski
A_mallard_Czech_Republic_186_orig_2023_H5N1_2023-01-05
A_domestic_duck_Poland_H75-K1T1_2023_H5N1_2023-01-16
A_domestic_duck_Poland_H74-T1_2023_H5N1_2023-01-16
A_domestic_duck_Poland_H89-T1_2023_H5N1_2023-01-20
A_domestic_duck_Poland_H96-T6_2023_H5N1_2023-01-23
A_domestic_duck_Poland_H84-T1_2023_H5N1_2023-01-18
A_turkey_Poland_H90-K4T4_2023_H5N1_2023-01-21
A_domestic_duck_Poland_H70-T1_2023_H5N1_2023-01-16
A_domestic_duck_Poland_H73-T1_2023_H5N1_2023-01-16
A_domestic_duck_Poland_H31-T_2023_H5N1_2023-01-09
A_raven_Poland_MB035_2023_H5N1_2023-01-22
A_mute_swan_Poland_MB037_2023_H5N1_2023-01-23
A_domestic_duck_Poland_H433_2022_H5N1_2022-12-16
A_gray_heron_Austria_23025449_2023_H5N1_2023-02-28
A_swan_Czech_Republic_811-2_2023_H5N1_2023-01-16
A_herring_gull_Austria_23010977-001_2023_H5N1_2023-01-30
A_mallard_1021_Czech_Republic_2023_H5N1_2023-01-16
A_domestic_duck_Poland_H86-T1_2023_H5N1_2023-01-18
A_mallard_Czech_Republic_1021_2023_H5N1_2023-01-16
A_herring_gull_Austria_23036780-003_2023_H5N1_2023-03-20
A_domestic_duck_Czech_Republic_636_orig_2023_H5N1_2023-01-12
A_swan_Czech_Republic_2022_2023_H5N1_2023-02-06
A_mute_swan_Slovakia_139_23VIR1686-5_2023_H5N1_2023-01-20
A_buzzard_Poland_MB275-TKL_2022_H5N1_2022-12-28
A_turkey_Poland_H23-K2T_2023_H5N1_2023-01-04
A_domestic_duck_Poland_H93-T1_2023_H5N1_2023-01-19
A_turkey_Poland_H82-T1_2023_H5N1_2023-01-18
A_domestic_duck_Poland_H77-T1_2023_H5N1_2023-01-17
A_domestic_duck_Poland_H78-K2T1_2023_H5N1_2023-01-17
A_domestic_duck_Poland_H72-T1_2023_H5N1_2023-01-16
A_Eurasian_eagle-owl_Slovenia_142_23VIR1977-6_2023_H5N1_2023-02-02
A_mute_swan_Poland_MB095-L1M_2023_H5N1_2023-02-14
A_laying_hen_Slovenia_251_23VIR1977-15_2023_H5N1_2023-02-21
A_chicken_Poland_H130-T_2023_H5N1_2023-02-20
A_buzzard_Italy_23VIR440-2_2023_H5N1_2023-01-04_Monticelli_D_Ongina_PC
A_mute_swan_Slovenia_236_23VIR1977-9_2023_H5N1_2023-02-17
A_mute_swan_Slovenia_237_23VIR1977-8_2023_H5N1_2023-02-20
A_mute_swan_Slovenia_124_23VIR1977-4_2023_H5N1_2023-01-31
A_domestic_duck_Poland_H447_2022_H5N1_2022-12-22
A_laying_hen_Poland_H443_2022_H5N1_2022-12-19
A_chicken_Czech_Republic_1690_orig_2023_H5N1_2023-01-30
A_chicken_Czech_Republic_1690_2023_H5N1_2023-01-30
A_great_egret_Austria_23000725-1_2023_H5N1_2023-01-04
A_great_egret_Austria_23000725-001_2023_H5N1_2023-01-04
CH
A_black-headed_gull_Poland_MB129-NJ_2023_H5N1_2023-03-22
A_black-headed_gull_Poland_MB131-J_2023_H5N1_2023-03-23
A_black-headed_gull_Poland_MB168-M2_2023_H5N1_2023-05-05
A_black-headed_gull_Poland_MB166_2023_H5N1_2023-05-05
A_black-headed_gull_Poland_MB180-NMJ_2023_H5N1_2023-05-09
A_common_tern_Poland_MB182-NJ_2023_H5N1_2023-05-10
A_black-headed_gull_Poland_MB210_2023_H5N1_2023-05-16
A_white_stork_Poland_MB274-M2_2023_H5N1_2023-07-07_malopolskie
A_white_stork_Poland_MB274-M3_2023_H5N1_2023-07-07_malopolskie
A_black-headed_gull_Poland_MB185-NJ_2023_H5N1_2023-05-10
A_black-headed_gull_Poland_MB176-M6_2023_H5N1_2023-05-05
A_black-headed_gull_Poland_MB209-NJ_2023_H5N1_2023
A_Mediterranean_gull_Poland_MB204_2023_H5N1_2023-05-08
A_black-headed_gull_Poland_MB141-T_2023_H5N1_2023-04-20
A_mute_swan_Poland_MB203-NJ_2023_H5N1_2023-05-11
A_black-headed_gull_Poland_MB198-NJ_2023_H5N1_2023-05-11
A_black-headed_gull_Poland_MB081_2023_H5N1_2023-02-07
A_black-headed_gull_Poland_MB121-M_2023_H5N1_2023-03-13
A_black-headed_gull_Poland_MB211_2023_H5N1_2023-05-14
BB
A_European_herring_gull_Netherlands_9_2023_H5N1_2023-03-03
A_Eurasian_Wigeon_Netherlands_3_2022_H5N1_2022-10-21
A_chicken_Czech_Republic_102_orig_2023_H5N1_2023-01-02
A_chicken_Czech_Republic_574_orig_2023_H5N1_2023-01-11
A_chicken_Czech_Republic_574_2023_H5N1_2023-01-11
A_chicken_Czech_Republic_67_orig_2023_H5N1_2022-12-30
A_chicken_Czech_Republic_727_orig_2023_H5N1_2023-01-15
96
100
100
98
93
95
89
95
98
99
0.02

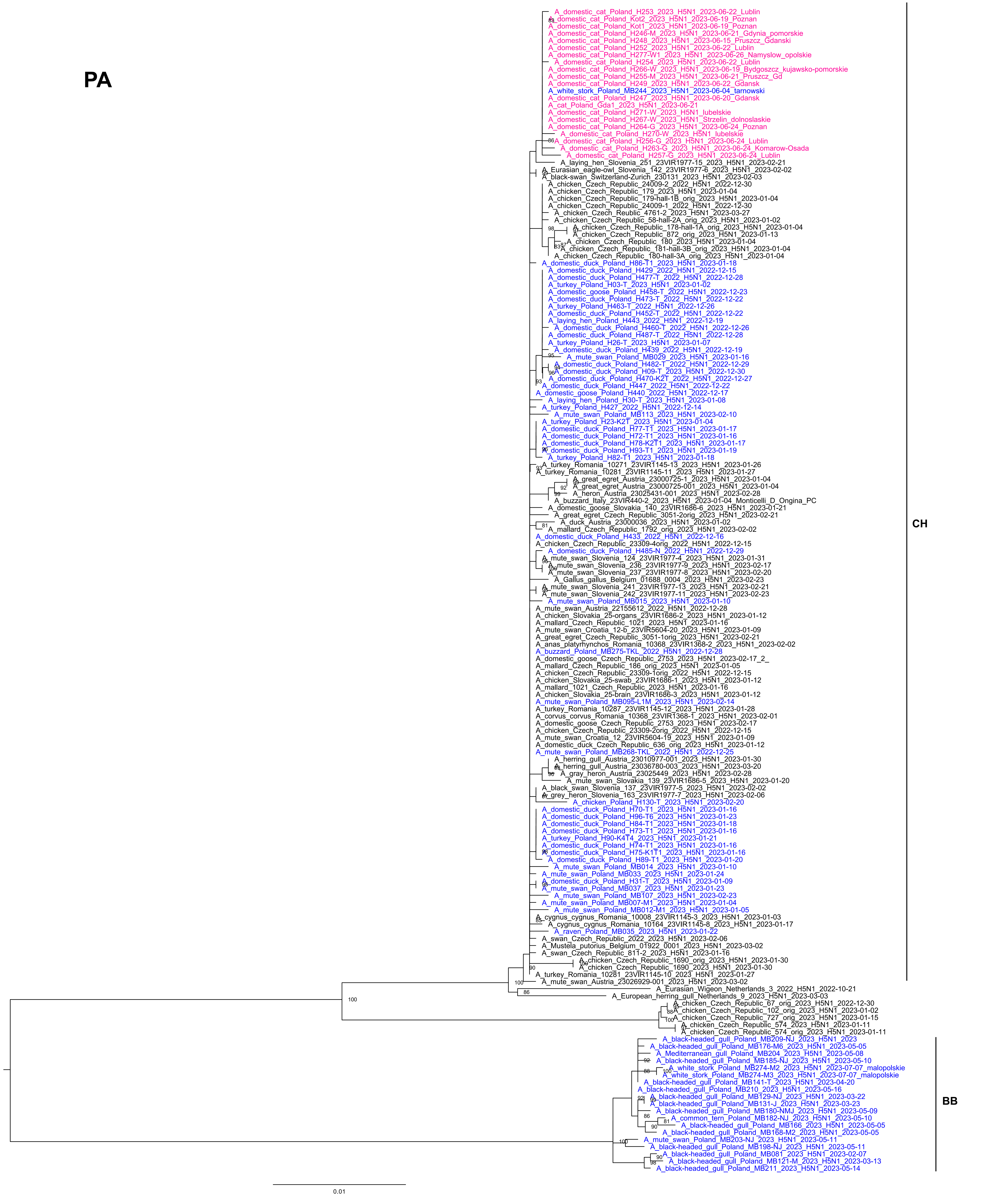
PA
A_domestic_cat_Poland_H253_2023_H5N1_2023-06-22_Lublin
A_domestic_cat_Poland_Kot2_2023_H5N1_2023-06-19_Poznan
A_domestic_cat_Poland_Kot1_2023_H5N1_2023-06-19_Poznan
A_domestic_cat_Poland_H246-M_2023_H5N1_2023-06-21_Gdynia_pomorskie
A_domestic_cat_Poland_H248_2023_H5N1_2023-06-15_Pruszcz_Gdanski
A_domestic_cat_Poland_H252_2023_H5N1_2023-06-22_Lublin
A_domestic_cat_Poland_H277-W1_2023_H5N1_2023-06-26_Namyslow_opolskie
A_domestic_cat_Poland_H254_2023_H5N1_2023-06-22_Lublin
A_domestic_cat_Poland_H266-W_2023_H5N1_2023-06-19_Bydgoszcz_kujawsko-pomorskie
A_domestic_cat_Poland_H255-M_2023_H5N1_2023-06-21_Pruszcz_Gd
A_domestic_cat_Poland_H249_2023_H5N1_2023-06-22_Gdansk
A_white_stork_Poland_MB244_2023_H5N1_2023-06-04_tarnowski
A_domestic_cat_Poland_H247_2023_H5N1_2023-06-20_Gdansk
A_cat_Poland_Gda1_2023_H5N1_2023-06-21
A_domestic_cat_Poland_H271-W_2023_H5N1_lubelskie
A_domestic_cat_Poland_H267-W_2023_H5N1_Strzelin_dolnoslaskie
A_domestic_cat_Poland_H264-G_2023_H5N1_2023-06-24_Poznan
A_domestic_cat_Poland_H270-W_2023_H5N1_lubelskie
A_domestic_cat_Poland_H256-G_2023_H5N1_2023-06-24_Lublin
A_domestic_cat_Poland_H263-G_2023_H5N1_2023-06-24_Komarow-Osada
A_domestic_cat_Poland_H257-G_2023_H5N1_2023-06-24_Lublin
A_laying_hen_Slovenia_251_23VIR1977-15_2023_H5N1_2023-02-21
A_Eurasian_eagle-owl_Slovenia_142_23VIR1977-6_2023_H5N1_2023-02-02
A_black-swan_Switzerland-Zurich_230131_2023_H5N1_2023-02-03
A_chicken_Czech_Republic_24009-2_2022_H5N1_2022-12-30
A_chicken_Czech_Republic_179_2023_H5N1_2023-01-04
A_chicken_Czech_Republic_179-hall-1B_orig_2023_H5N1_2023-01-04
A_chicken_Czech_Republic_24009-1_2022_H5N1_2022-12-30
A_chicken_Czech_Reublic_4761-2_2023_H5N1_2023-03-27
A_chicken_Czech_Republic_58-hall-2A_orig_2023_H5N1_2023-01-02
A_chicken_Czech_Republic_178-hall-1A_orig_2023_H5N1_2023-01-04
A_chicken_Czech_Republic_872_orig_2023_H5N1_2023-01-13
A_chicken_Czech_Republic_180_2023_H5N1_2023-01-04
A_chicken_Czech_Republic_181-hall-3B_orig_2023_H5N1_2023-01-04
A_chicken_Czech_Republic_180-hall-3A_orig_2023_H5N1_2023-01-04
A_domestic_duck_Poland_H86-T1_2023_H5N1_2023-01-18
A_domestic_duck_Poland_H429_2022_H5N1_2022-12-15
A_domestic_duck_Poland_H477-T_2022_H5N1_2022-12-28
A_turkey_Poland_H03-T_2023_H5N1_2023-01-02
A_domestic_goose_Poland_H458-T_2022_H5N1_2022-12-23
A_domestic_duck_Poland_H473-T_2022_H5N1_2022-12-22
A_turkey_Poland_H463-T_2022_H5N1_2022-12-26
A_domestic_duck_Poland_H452-T_2022_H5N1_2022-12-22
A_laying_hen_Poland_H443_2022_H5N1_2022-12-19
A_domestic_duck_Poland_H460-T_2022_H5N1_2022-12-26
A_domestic_duck_Poland_H487-T_2022_H5N1_2022-12-28
A_turkey_Poland_H26-T_2023_H5N1_2023-01-07
A_domestic_duck_Poland_H439_2022_H5N1_2022-12-19
A_mute_swan_Poland_MB029_2023_H5N1_2023-01-16
A_domestic_duck_Poland_H482-T_2022_H5N1_2022-12-29
A_domestic_duck_Poland_H09-T_2023_H5N1_2022-12-30
A_domestic_duck_Poland_H470-K2T_2022_H5N1_2022-12-27
A_domestic_duck_Poland_H447_2022_H5N1_2022-12-22
A_domestic_goose_Poland_H440_2022_H5N1_2022-12-17
A_laying_hen_Poland_H30-T_2023_H5N1_2023-01-08
A_turkey_Poland_H427_2022_H5N1_2022-12-14
A_mute_swan_Poland_MB113_2023_H5N1_2023-02-10
A_turkey_Poland_H23-K2T_2023_H5N1_2023-01-04
A_domestic_duck_Poland_H77-T1_2023_H5N1_2023-01-17
A_domestic_duck_Poland_H72-T1_2023_H5N1_2023-01-16
A_domestic_duck_Poland_H78-K2T1_2023_H5N1_2023-01-17
A_domestic_duck_Poland_H93-T1_2023_H5N1_2023-01-19
A_turkey_Poland_H82-T1_2023_H5N1_2023-01-18
A_turkey_Romania_10271_23VIR1145-13_2023_H5N1_2023-01-26
A_turkey_Romania_10281_23VIR1145-11_2023_H5N1_2023-01-27
A_great_egret_Austria_23000725-1_2023_H5N1_2023-01-04
A_great_egret_Austria_23000725-001_2023_H5N1_2023-01-04
A_heron_Austria_23025431-001_2023_H5N1_2023-02-28
A_buzzard_Italy_23VIR440-2_2023_H5N1_2023-01-04_Monticelli_D_Ongina_PC
A_domestic_goose_Slovakia_140_23VIR1686-6_2023_H5N1_2023-01-21
A_great_egret_Czech_Republic_3051-2orig_2023_H5N1_2023-02-21
A_duck_Austria_23000036_2023_H5N1_2023-01-02
A_mallard_Czech_Republic_1792_orig_2023_H5N1_2023-02-02
A_domestic_duck_Poland_H433_2022_H5N1_2022-12-16
A_chicken_Czech_Republic_23309-4orig_2022_H5N1_2022-12-15
A_domestic_duck_Poland_H485-N_2022_H5N1_2022-12-29
A_mute_swan_Slovenia_124_23VIR1977-4_2023_H5N1_2023-01-31
A_mute_swan_Slovenia_236_23VIR1977-9_2023_H5N1_2023-02-17
A_mute_swan_Slovenia_237_23VIR1977-8_2023_H5N1_2023-02-20
A_Gallus_gallus_Belgium_01688_0004_2023_H5N1_2023-02-23
A_mute_swan_Slovenia_241_23VIR1977-13_2023_H5N1_2023-02-21
A_mute_swan_Slovenia_242_23VIR1977-11_2023_H5N1_2023-02-23
A_mute_swan_Poland_MB015_2023_H5N1_2023-01-10
A_mute_swan_Austria_22155612_2022_H5N1_2022-12-28
A_chicken_Slovakia_25-organs_23VIR1686-2_2023_H5N1_2023-01-12
A_mallard_Czech_Republic_1021_2023_H5N1_2023-01-16
A_mute_swan_Croatia_12-b_23VIR5604-20_2023_H5N1_2023-01-09
A_great_egret_Czech_Republic_3051-1orig_2023_H5N1_2023-02-21
A_anas_platyrhynchos_Romania_10368_23VIR1368-2_2023_H5N1_2023-02-02
A_buzzard_Poland_MB275-TKL_2022_H5N1_2022-12-28
A_domestic_goose_Czech_Republic_2753_2023_H5N1_2023-02-17_2_
A_mallard_Czech_Republic_186_orig_2023_H5N1_2023-01-05
A_chicken_Czech_Republic_23309-1orig_2022_H5N1_2022-12-15
A_chicken_Slovakia_25-swab_23VIR1686-1_2023_H5N1_2023-01-12
A_mallard_1021_Czech_Republic_2023_H5N1_2023-01-16
A_chicken_Slovakia_25-brain_23VIR1686-3_2023_H5N1_2023-01-12
A_mute_swan_Poland_MB095-L1M_2023_H5N1_2023-02-14
A_turkey_Romania_10287_23VIR1145-12_2023_H5N1_2023-01-28
A_corvus_corvus_Romania_10368_23VIR1368-1_2023_H5N1_2023-02-01
A_domestic_goose_Czech_Republic_2753_2023_H5N1_2023-02-17
A_chicken_Czech_Republic_23309-2orig_2022_H5N1_2022-12-15
A_mute_swan_Croatia_12_23VIR5604-19_2023_H5N1_2023-01-09
A_domestic_duck_Czech_Republic_636_orig_2023_H5N1_2023-01-12
A_mute_swan_Poland_MB268-TKL_2022_H5N1_2022-12-25
A_herring_gull_Austria_23010977-001_2023_H5N1_2023-01-30
A_herring_gull_Austria_23036780-003_2023_H5N1_2023-03-20
A_gray_heron_Austria_23025449_2023_H5N1_2023-02-28
A_mute_swan_Slovakia_139_23VIR1686-5_2023_H5N1_2023-01-20
A_black_swan_Slovenia_137_23VIR1977-5_2023_H5N1_2023-02-02
A_grey_heron_Slovenia_163_23VIR1977-7_2023_H5N1_2023-02-06
A_chicken_Poland_H130-T_2023_H5N1_2023-02-20
A_domestic_duck_Poland_H70-T1_2023_H5N1_2023-01-16
A_domestic_duck_Poland_H96-T6_2023_H5N1_2023-01-23
A_domestic_duck_Poland_H84-T1_2023_H5N1_2023-01-18
A_domestic_duck_Poland_H73-T1_2023_H5N1_2023-01-16
A_turkey_Poland_H90-K4T4_2023_H5N1_2023-01-21
A_domestic_duck_Poland_H74-T1_2023_H5N1_2023-01-16
A_domestic_duck_Poland_H75-K1T1_2023_H5N1_2023-01-16
A_domestic_duck_Poland_H89-T1_2023_H5N1_2023-01-20
A_mute_swan_Poland_MB014_2023_H5N1_2023-01-10
A_mute_swan_Poland_MB033_2023_H5N1_2023-01-24
A_domestic_duck_Poland_H31-T_2023_H5N1_2023-01-09
A_mute_swan_Poland_MB037_2023_H5N1_2023-01-23
A_mute_swan_Poland_MB107_2023_H5N1_2023-02-23
A_mute_swan_Poland_MB007-M1_2023_H5N1_2023-01-04
A_mute_swan_Poland_MB012-M1_2023_H5N1_2023-01-05
A_cygnus_cygnus_Romania_10008_23VIR1145-3_2023_H5N1_2023-01-03
A_cygnus_cygnus_Romania_10164_23VIR1145-8_2023_H5N1_2023-01-17
A_raven_Poland_MB035_2023_H5N1_2023-01-22
A_swan_Czech_Republic_2022_2023_H5N1_2023-02-06
A_Mustela_putorius_Belgium_01922_0001_2023_H5N1_2023-03-02
A_swan_Czech_Republic_811-2_2023_H5N1_2023-01-16
A_chicken_Czech_Republic_1690_orig_2023_H5N1_2023-01-30
A_chicken_Czech_Republic_1690_2023_H5N1_2023-01-30
A_turkey_Romania_10281_23VIR1145-10_2023_H5N1_2023-01-27
A_mute_swan_Austria_23026929-001_2023_H5N1_2023-03-02
A_Eurasian_Wigeon_Netherlands_3_2022_H5N1_2022-10-21
A_European_herring_gull_Netherlands_9_2023_H5N1_2023-03-03
A_chicken_Czech_Republic_67_orig_2023_H5N1_2022-12-30
A_chicken_Czech_Republic_102_orig_2023_H5N1_2023-01-02
A_chicken_Czech_Republic_727_orig_2023_H5N1_2023-01-15
A_chicken_Czech_Republic_574_2023_H5N1_2023-01-11
A_chicken_Czech_Republic_574_orig_2023_H5N1_2023-01-11
A_black-headed_gull_Poland_MB209-NJ_2023_H5N1_2023
A_black-headed_gull_Poland_MB176-M6_2023_H5N1_2023-05-05
A_Mediterranean_gull_Poland_MB204_2023_H5N1_2023-05-08
A_black-headed_gull_Poland_MB185-NJ_2023_H5N1_2023-05-10
A_white_stork_Poland_MB274-M2_2023_H5N1_2023-07-07_malopolskie
A_white_stork_Poland_MB274-M3_2023_H5N1_2023-07-07_malopolskie
A_black-headed_gull_Poland_MB141-T_2023_H5N1_2023-04-20
A_black-headed_gull_Poland_MB210_2023_H5N1_2023-05-16
A_black-headed_gull_Poland_MB129-NJ_2023_H5N1_2023-03-22
A_black-headed_gull_Poland_MB131-J_2023_H5N1_2023-03-23
A_black-headed_gull_Poland_MB180-NMJ_2023_H5N1_2023-05-09
A_common_tern_Poland_MB182-NJ_2023_H5N1_2023-05-10
A_black-headed_gull_Poland_MB166_2023_H5N1_2023-05-05
A_black-headed_gull_Poland_MB168-M2_2023_H5N1_2023-05-05
A_mute_swan_Poland_MB203-NJ_2023_H5N1_2023-05-11
A_black-headed_gull_Poland_MB198-NJ_2023_H5N1_2023-05-11
A_black-headed_gull_Poland_MB081_2023_H5N1_2023-02-07
A_black-headed_gull_Poland_MB121-M_2023_H5N1_2023-03-13
A_black-headed_gull_Poland_MB211_2023_H5N1_2023-05-14
CH
BB
0.01

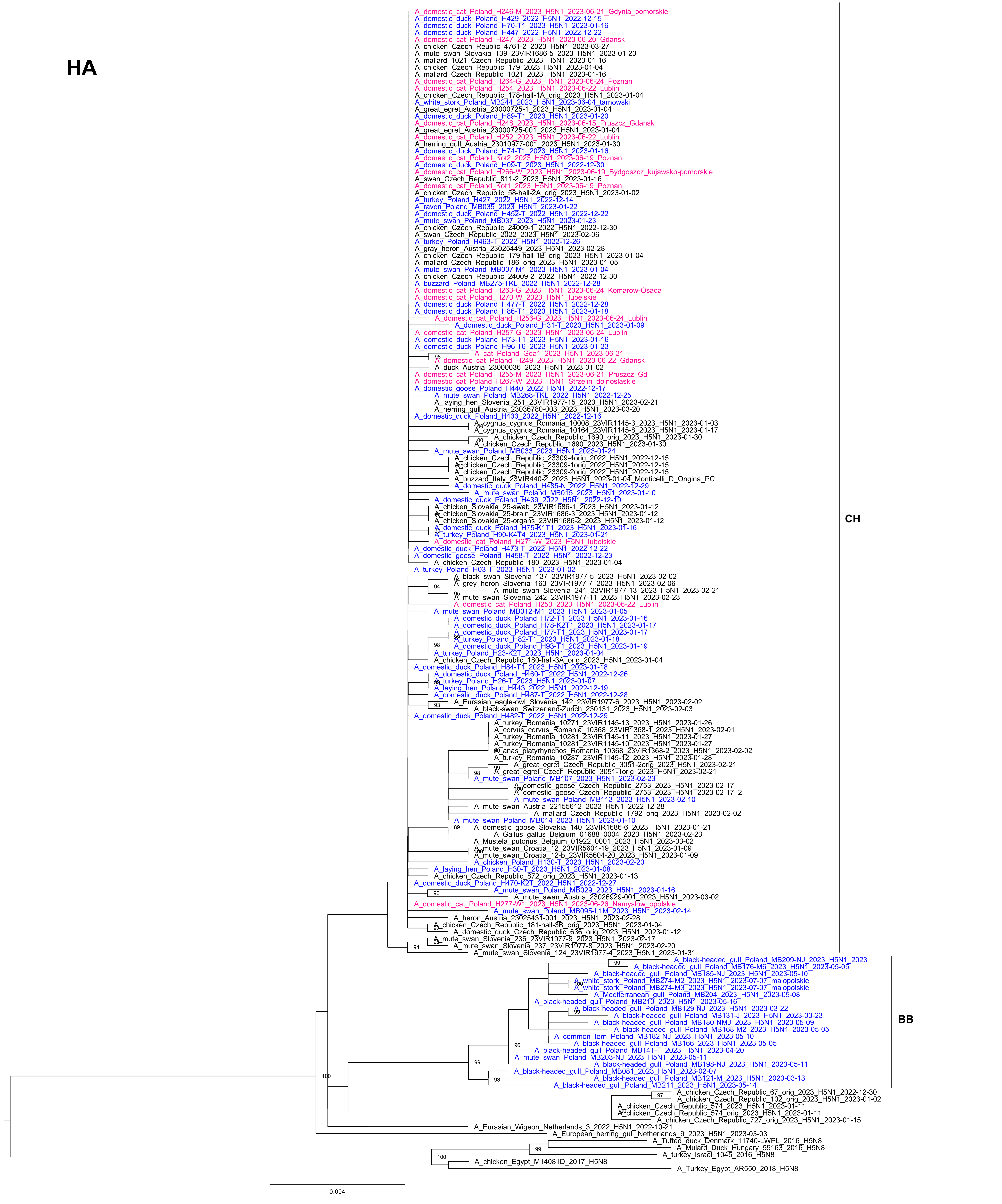
HA
A_domestic_cat_Poland_H246-M_2023_H5N1_2023-06-21_Gdynia_pomorskie
A_domestic_duck_Poland_H429_2022_H5N1_2022-12-15
A_domestic_duck_Poland_H70-T1_2023_H5N1_2023-01-16
A_domestic_duck_Poland_H447_2022_H5N1_2022-12-22
A_domestic_cat_Poland_H247_2023_H5N1_2023-06-20_Gdansk
A_chicken_Czech_Reublic_4761-2_2023_H5N1_2023-03-27
A_mute_swan_Slovakia_139_23VIR1686-5_2023_H5N1_2023-01-20
A_mallard_1021_Czech_Republic_2023_H5N1_2023-01-16
A_chicken_Czech_Republic_179_2023_H5N1_2023-01-04
A_mallard_Czech_Republic_1021_2023_H5N1_2023-01-16
A_domestic_cat_Poland_H264-G_2023_H5N1_2023-06-24_Poznan
A_domestic_cat_Poland_H254_2023_H5N1_2023-06-22_Lublin
A_chicken_Czech_Republic_178-hall-1A_orig_2023_H5N1_2023-01-04
A_white_stork_Poland_MB244_2023_H5N1_2023-06-04_tarnowski
A_great_egret_Austria_23000725-1_2023_H5N1_2023-01-04
A_domestic_duck_Poland_H89-T1_2023_H5N1_2023-01-20
A_domestic_cat_Poland_H248_2023_H5N1_2023-06-15_Pruszcz_Gdanski
A_great_egret_Austria_23000725-001_2023_H5N1_2023-01-04
A_domestic_cat_Poland_H252_2023_H5N1_2023-06-22_Lublin
A_herring_gull_Austria_23010977-001_2023_H5N1_2023-01-30
A_domestic_duck_Poland_H74-T1_2023_H5N1_2023-01-16
A_domestic_cat_Poland_Kot2_2023_H5N1_2023-06-19_Poznan
A_domestic_duck_Poland_H09-T_2023_H5N1_2022-12-30
A_domestic_cat_Poland_H266-W_2023_H5N1_2023-06-19_Bydgoszcz_kujawsko-pomorskie
A_swan_Czech_Republic_811-2_2023_H5N1_2023-01-16
A_domestic_cat_Poland_Kot1_2023_H5N1_2023-06-19_Poznan
A_chicken_Czech_Republic_58-hall-2A_orig_2023_H5N1_2023-01-02
A_turkey_Poland_H427_2022_H5N1_2022-12-14
A_raven_Poland_MB035_2023_H5N1_2023-01-22
A_domestic_duck_Poland_H452-T_2022_H5N1_2022-12-22
A_mute_swan_Poland_MB037_2023_H5N1_2023-01-23
A_chicken_Czech_Republic_24009-1_2022_H5N1_2022-12-30
A_swan_Czech_Republic_2022_2023_H5N1_2023-02-06
A_turkey_Poland_H463-T_2022_H5N1_2022-12-26
A_gray_heron_Austria_23025449_2023_H5N1_2023-02-28
A_chicken_Czech_Republic_179-hall-1B_orig_2023_H5N1_2023-01-04
A_mallard_Czech_Republic_186_orig_2023_H5N1_2023-01-05
A_mute_swan_Poland_MB007-M1_2023_H5N1_2023-01-04
A_chicken_Czech_Republic_24009-2_2022_H5N1_2022-12-30
A_buzzard_Poland_MB275-TKL_2022_H5N1_2022-12-28
A_domestic_cat_Poland_H263-G_2023_H5N1_2023-06-24_Komarow-Osada
A_domestic_cat_Poland_H270-W_2023_H5N1_lubelskie
A_domestic_duck_Poland_H477-T_2022_H5N1_2022-12-28
A_domestic_duck_Poland_H86-T1_2023_H5N1_2023-01-18
A_domestic_cat_Poland_H256-G_2023_H5N1_2023-06-24_Lublin
A_domestic_duck_Poland_H31-T_2023_H5N1_2023-01-09
A_domestic_cat_Poland_H257-G_2023_H5N1_2023-06-24_Lublin
A_domestic_duck_Poland_H73-T1_2023_H5N1_2023-01-16
A_domestic_duck_Poland_H96-T6_2023_H5N1_2023-01-23
98
A_cat_Poland_Gda1_2023_H5N1_2023-06-21
A_domestic_cat_Poland_H249_2023_H5N1_2023-06-22_Gdansk
A_duck_Austria_23000036_2023_H5N1_2023-01-02
A_domestic_cat_Poland_H255-M_2023_H5N1_2023-06-21_Pruszcz_Gd
A_domestic_cat_Poland_H267-W_2023_H5N1_Strzelin_dolnoslaskie
A_domestic_goose_Poland_H440_2022_H5N1_2022-12-17
A_mute_swan_Poland_MB268-TKL_2022_H5N1_2022-12-25
A_laying_hen_Slovenia_251_23VIR1977-15_2023_H5N1_2023-02-21
A_herring_gull_Austria_23036780-003_2023_H5N1_2023-03-20
A_domestic_duck_Poland_H433_2022_H5N1_2022-12-16
A_cygnus_cygnus_Romania_10008_23VIR1145-3_2023_H5N1_2023-01-03
100
A_cygnus_cygnus_Romania_10164_23VIR1145-8_2023_H5N1_2023-01-17
A_chicken_Czech_Republic_1690_orig_2023_H5N1_2023-01-30
100
A_chicken_Czech_Republic_1690_2023_H5N1_2023-01-30
A_mute_swan_Poland_MB033_2023_H5N1_2023-01-24
A_chicken_Czech_Republic_23309-4orig_2022_H5N1_2022-12-15
A_chicken_Czech_Republic_23309-1orig_2022_H5N1_2022-12-15
A_chicken_Czech_Republic_23309-2orig_2022_H5N1_2022-12-15
A_buzzard_Italy_23VIR440-2_2023_H5N1_2023-01-04_Monticelli_D_Ongina_PC
A_domestic_duck_Poland_H485-N_2022_H5N1_2022-12-29
A_mute_swan_Poland_MB015_2023_H5N1_2023-01-10
A_domestic_duck_Poland_H439_2022_H5N1_2022-12-19
A_chicken_Slovakia_25-swab_23VIR1686-1_2023_H5N1_2023-01-12
A_chicken_Slovakia_25-brain_23VIR1686-3_2023_H5N1_2023-01-12
A_chicken_Slovakia_25-organs_23VIR1686-2_2023_H5N1_2023-01-12
A_domestic_duck_Poland_H75-K1T1_2023_H5N1_2023-01-16
A_turkey_Poland_H90-K4T4_2023_H5N1_2023-01-21
A_domestic_cat_Poland_H271-W_2023_H5N1_lubelskie
A_domestic_duck_Poland_H473-T_2022_H5N1_2022-12-22
A_domestic_goose_Poland_H458-T_2022_H5N1_2022-12-23
A_chicken_Czech_Republic_180_2023_H5N1_2023-01-04
A_turkey_Poland_H03-T_2023_H5N1_2023-01-02
A_black_swan_Slovenia_137_23VIR1977-5_2023_H5N1_2023-02-02
94
A_grey_heron_Slovenia_163_23VIR1977-7_2023_H5N1_2023-02-06
A_mute_swan_Slovenia_241_23VIR1977-13_2023_H5N1_2023-02-21
95
A_mute_swan_Slovenia_242_23VIR1977-11_2023_H5N1_2023-02-23
A_domestic_cat_Poland_H253_2023_H5N1_2023-06-22_Lublin
A_mute_swan_Poland_MB012-M1_2023_H5N1_2023-01-05
A_domestic_duck_Poland_H72-T1_2023_H5N1_2023-01-16
A_domestic_duck_Poland_H78-K2T1_2023_H5N1_2023-01-17
A_domestic_duck_Poland_H77-T1_2023_H5N1_2023-01-17
A_turkey_Poland_H82-T1_2023_H5N1_2023-01-18
98
A_domestic_duck_Poland_H93-T1_2023_H5N1_2023-01-19
A_turkey_Poland_H23-K2T_2023_H5N1_2023-01-04
A_chicken_Czech_Republic_180-hall-3A_orig_2023_H5N1_2023-01-04
A_domestic_duck_Poland_H84-T1_2023_H5N1_2023-01-18
A_domestic_duck_Poland_H460-T_2022_H5N1_2022-12-26
A_turkey_Poland_H26-T_2023_H5N1_2023-01-07
A_laying_hen_Poland_H443_2022_H5N1_2022-12-19
A_domestic_duck_Poland_H487-T_2022_H5N1_2022-12-28
93
A_Eurasian_eagle-owl_Slovenia_142_23VIR1977-6_2023_H5N1_2023-02-02
A_black-swan_Switzerland-Zurich_230131_2023_H5N1_2023-02-03
A_domestic_duck_Poland_H482-T_2022_H5N1_2022-12-29
A_turkey_Romania_10271_23VIR1145-13_2023_H5N1_2023-01-26
A_corvus_corvus_Romania_10368_23VIR1368-1_2023_H5N1_2023-02-01
A_turkey_Romania_10281_23VIR1145-11_2023_H5N1_2023-01-27
A_turkey_Romania_10281_23VIR1145-10_2023_H5N1_2023-01-27
A_anas_platyrhynchos_Romania_10368_23VIR1368-2_2023_H5N1_2023-02-02
A_turkey_Romania_10287_23VIR1145-12_2023_H5N1_2023-01-28
99
98
A_great_egret_Czech_Republic_3051-2orig_2023_H5N1_2023-02-21
A_great_egret_Czech_Republic_3051-1orig_2023_H5N1_2023-02-21
A_mute_swan_Poland_MB107_2023_H5N1_2023-02-23
A_domestic_goose_Czech_Republic_2753_2023_H5N1_2023-02-17
100
A_domestic_goose_Czech_Republic_2753_2023_H5N1_2023-02-17_2_
A_mute_swan_Poland_MB113_2023_H5N1_2023-02-10
A_mute_swan_Austria_22155612_2022_H5N1_2022-12-28
A_mallard_Czech_Republic_1792_orig_2023_H5N1_2023-02-02
A_mute_swan_Poland_MB014_2023_H5N1_2023-01-10
89
A_domestic_goose_Slovakia_140_23VIR1686-6_2023_H5N1_2023-01-21
A_Gallus_gallus_Belgium_01688_0004_2023_H5N1_2023-02-23
A_Mustela_putorius_Belgium_01922_0001_2023_H5N1_2023-03-02
A_mute_swan_Croatia_12_23VIR5604-19_2023_H5N1_2023-01-09
100
A_mute_swan_Croatia_12-b_23VIR5604-20_2023_H5N1_2023-01-09
A_chicken_Poland_H130-T_2023_H5N1_2023-02-20
A_laying_hen_Poland_H30-T_2023_H5N1_2023-01-08
A_chicken_Czech_Republic_872_orig_2023_H5N1_2023-01-13
A_domestic_duck_Poland_H470-K2T_2022_H5N1_2022-12-27
A_mute_swan_Poland_MB029_2023_H5N1_2023-01-16
90
A_mute_swan_Austria_23026929-001_2023_H5N1_2023-03-02
A_domestic_cat_Poland_H277-W1_2023_H5N1_2023-06-26_Namyslow_opolskie
A_mute_swan_Poland_MB095-L1M_2023_H5N1_2023-02-14
A_heron_Austria_23025431-001_2023_H5N1_2023-02-28
97
A_chicken_Czech_Republic_181-hall-3B_orig_2023_H5N1_2023-01-04
A_domestic_duck_Czech_Republic_636_orig_2023_H5N1_2023-01-12
A_mute_swan_Slovenia_236_23VIR1977-9_2023_H5N1_2023-02-17
96
94
A_mute_swan_Slovenia_237_23VIR1977-8_2023_H5N1_2023-02-20
A_mute_swan_Slovenia_124_23VIR1977-4_2023_H5N1_2023-01-31
CH
99
A_black-headed_gull_Poland_MB209-NJ_2023_H5N1_2023
A_black-headed_gull_Poland_MB176-M6_2023_H5N1_2023-05-05
A_black-headed_gull_Poland_MB185-NJ_2023_H5N1_2023-05-10
A_white_stork_Poland_MB274-M2_2023_H5N1_2023-07-07_malopolskie
100
A_white_stork_Poland_MB274-M3_2023_H5N1_2023-07-07_malopolskie
A_Mediterranean_gull_Poland_MB204_2023_H5N1_2023-05-08
A_black-headed_gull_Poland_MB210_2023_H5N1_2023-05-16
A_black-headed_gull_Poland_MB129-NJ_2023_H5N1_2023-03-22
99
A_black-headed_gull_Poland_MB131-J_2023_H5N1_2023-03-23
A_black-headed_gull_Poland_MB180-NMJ_2023_H5N1_2023-05-09
A_black-headed_gull_Poland_MB168-M2_2023_H5N1_2023-05-05
A_common_tern_Poland_MB182-NJ_2023_H5N1_2023-05-10
A_black-headed_gull_Poland_MB166_2023_H5N1_2023-05-05
96
A_black-headed_gull_Poland_MB141-T_2023_H5N1_2023-04-20
99
A_mute_swan_Poland_MB203-NJ_2023_H5N1_2023-05-11
A_black-headed_gull_Poland_MB198-NJ_2023_H5N1_2023-05-11
A_black-headed_gull_Poland_MB081_2023_H5N1_2023-02-07
A_black-headed_gull_Poland_MB121-M_2023_H5N1_2023-03-13
93
A_black-headed_gull_Poland_MB211_2023_H5N1_2023-05-14
BB
100
97
A_chicken_Czech_Republic_67_orig_2023_H5N1_2022-12-30
A_chicken_Czech_Republic_102_orig_2023_H5N1_2023-01-02
A_chicken_Czech_Republic_574_2023_H5N1_2023-01-11
100
A_chicken_Czech_Republic_574_orig_2023_H5N1_2023-01-11
A_chicken_Czech_Republic_727_orig_2023_H5N1_2023-01-15
A_Eurasian_Wigeon_Netherlands_3_2022_H5N1_2022-10-21
A_European_herring_gull_Netherlands_9_2023_H5N1_2023-03-03
A_Tufted_duck_Denmark_11740-LWPL_2016_H5N8
99
A_Mulard_Duck_Hungary_59163_2016_H5N8
100
A_turkey_Israel_1045_2016_H5N8
A_chicken_Egypt_M14081D_2017_H5N8
A_Turkey_Egypt_AR550_2018_H5N8
0.004

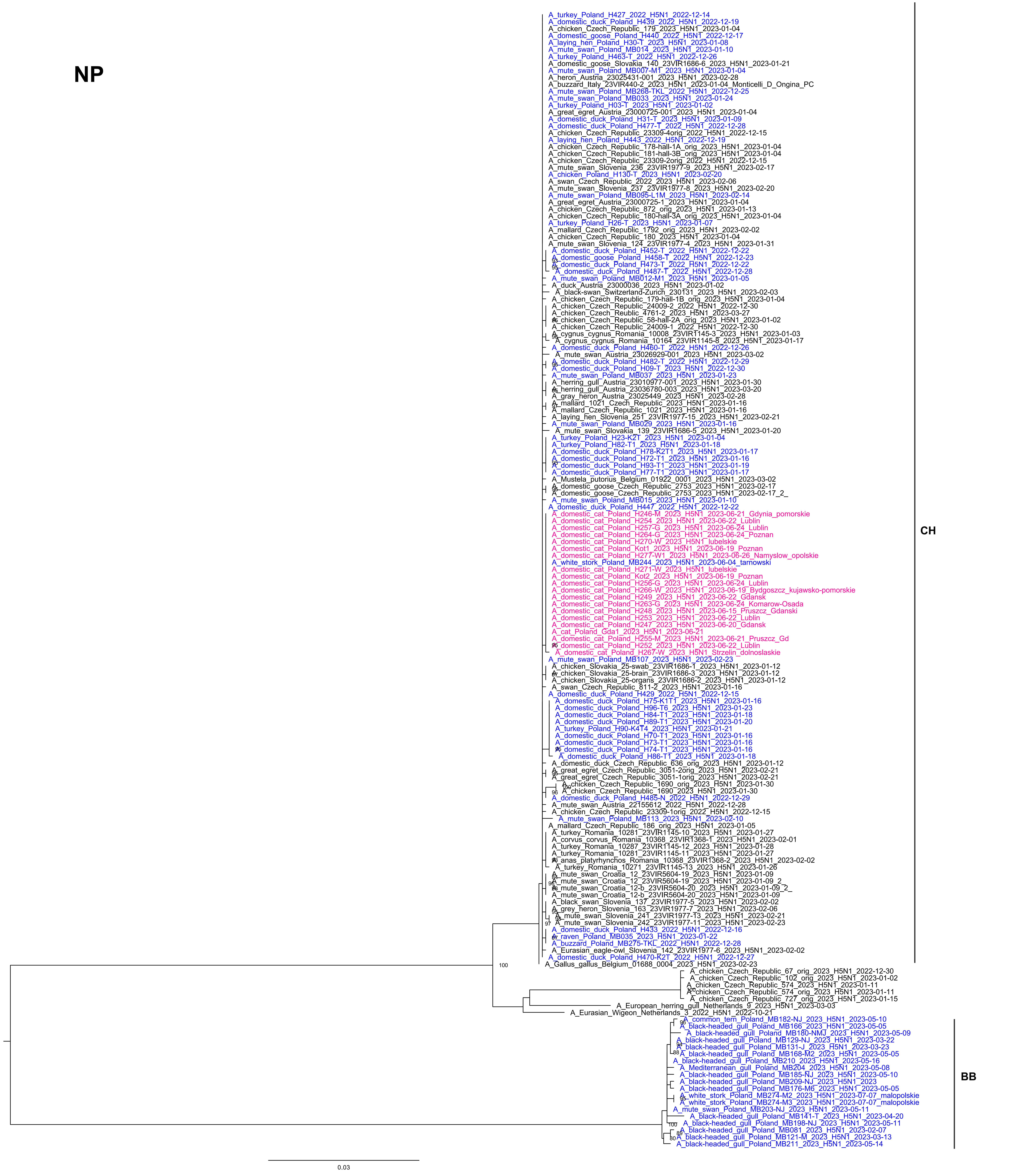

NP
A_turkey_Poland_H427_2022_H5N1_2022-12-14
A_domestic_duck_Poland_H439_2022_H5N1_2022-12-19
A_chicken_Czech_Republic_179_2023_H5N1_2023-01-04
A_domestic_goose_Poland_H440_2022_H5N1_2022-12-17
A_laying_hen_Poland_H30-T_2023_H5N1_2023-01-08
A_mute_swan_Poland_MB014_2023_H5N1_2023-01-10
A_turkey_Poland_H463-T_2022_H5N1_2022-12-26
A_domestic_goose_Slovakia_140_23VIR1686-6_2023_H5N1_2023-01-21
A_mute_swan_Poland_MB007-M1_2023_H5N1_2023-01-04
A_heron_Austria_23025431-001_2023_H5N1_2023-02-28
A_buzzard_Italy_23VIR440-2_2023_H5N1_2023-01-04_Monticelli_D_Ongina_PC
A_mute_swan_Poland_MB268-TKL_2022_H5N1_2022-12-25
A_mute_swan_Poland_MB033_2023_H5N1_2023-01-24
A_turkey_Poland_H03-T_2023_H5N1_2023-01-02
A_great_egret_Austria_23000725-001_2023_H5N1_2023-01-04
A_domestic_duck_Poland_H31-T_2023_H5N1_2023-01-09
A_domestic_duck_Poland_H477-T_2022_H5N1_2022-12-28
A_chicken_Czech_Republic_23309-4orig_2022_H5N1_2022-12-15
A_laying_hen_Poland_H443_2022_H5N1_2022-12-19
A_chicken_Czech_Republic_178-hall-1A_orig_2023_H5N1_2023-01-04
A_chicken_Czech_Republic_181-hall-3B_orig_2023_H5N1_2023-01-04
A_chicken_Czech_Republic_23309-2orig_2022_H5N1_2022-12-15
A_mute_swan_Slovenia_236_23VIR1977-9_2023_H5N1_2023-02-17
A_chicken_Poland_H130-T_2023_H5N1_2023-02-20
A_swan_Czech_Republic_2022_2023_H5N1_2023-02-06
A_mute_swan_Slovenia_237_23VIR1977-8_2023_H5N1_2023-02-20
A_mute_swan_Poland_MB095-L1M_2023_H5N1_2023-02-14
A_great_egret_Austria_23000725-1_2023_H5N1_2023-01-04
A_chicken_Czech_Republic_872_orig_2023_H5N1_2023-01-13
A_chicken_Czech_Republic_180-hall-3A_orig_2023_H5N1_2023-01-04
A_turkey_Poland_H26-T_2023_H5N1_2023-01-07
A_mallard_Czech_Republic_1792_orig_2023_H5N1_2023-02-02
A_chicken_Czech_Republic_180_2023_H5N1_2023-01-04
A_mute_swan_Slovenia_124_23VIR1977-4_2023_H5N1_2023-01-31
A_domestic_duck_Poland_H452-T_2022_H5N1_2022-12-22
A_domestic_goose_Poland_H458-T_2022_H5N1_2022-12-23
A_domestic_duck_Poland_H473-T_2022_H5N1_2022-12-22
A_domestic_duck_Poland_H487-T_2022_H5N1_2022-12-28
A_mute_swan_Poland_MB012-M1_2023_H5N1_2023-01-05
A_duck_Austria_23000036_2023_H5N1_2023-01-02
A_black-swan_Switzerland-Zurich_230131_2023_H5N1_2023-02-03
A_chicken_Czech_Republic_179-hall-1B_orig_2023_H5N1_2023-01-04
A_chicken_Czech_Republic_24009-2_2022_H5N1_2022-12-30
A_chicken_Czech_Reublic_4761-2_2023_H5N1_2023-03-27
A_chicken_Czech_Republic_58-hall-2A_orig_2023_H5N1_2023-01-02
A_chicken_Czech_Republic_24009-1_2022_H5N1_2022-12-30
A_cygnus_cygnus_Romania_10008_23VIR1145-3_2023_H5N1_2023-01-03
A_cygnus_cygnus_Romania_10164_23VIR1145-8_2023_H5N1_2023-01-17
A_domestic_duck_Poland_H460-T_2022_H5N1_2022-12-26
A_mute_swan_Austria_23026929-001_2023_H5N1_2023-03-02
A_domestic_duck_Poland_H482-T_2022_H5N1_2022-12-29
A_domestic_duck_Poland_H09-T_2023_H5N1_2022-12-30
A_mute_swan_Poland_MB037_2023_H5N1_2023-01-23
A_herring_gull_Austria_23010977-001_2023_H5N1_2023-01-30
A_herring_gull_Austria_23036780-003_2023_H5N1_2023-03-20
A_gray_heron_Austria_23025449_2023_H5N1_2023-02-28
A_mallard_1021_Czech_Republic_2023_H5N1_2023-01-16
A_mallard_Czech_Republic_1021_2023_H5N1_2023-01-16
A_laying_hen_Slovenia_251_23VIR1977-15_2023_H5N1_2023-02-21
A_mute_swan_Poland_MB029_2023_H5N1_2023-01-16
A_mute_swan_Slovakia_139_23VIR1686-5_2023_H5N1_2023-01-20
A_turkey_Poland_H23-K2T_2023_H5N1_2023-01-04
A_turkey_Poland_H82-T1_2023_H5N1_2023-01-18
A_domestic_duck_Poland_H78-K2T1_2023_H5N1_2023-01-17
A_domestic_duck_Poland_H72-T1_2023_H5N1_2023-01-16
A_domestic_duck_Poland_H93-T1_2023_H5N1_2023-01-19
A_domestic_duck_Poland_H77-T1_2023_H5N1_2023-01-17
A_Mustela_putorius_Belgium_01922_0001_2023_H5N1_2023-03-02
A_domestic_goose_Czech_Republic_2753_2023_H5N1_2023-02-17
A_domestic_goose_Czech_Republic_2753_2023_H5N1_2023-02-17_2_
A_mute_swan_Poland_MB015_2023_H5N1_2023-01-10
A_domestic_duck_Poland_H447_2022_H5N1_2022-12-22
A_domestic_cat_Poland_H246-M_2023_H5N1_2023-06-21_Gdynia_pomorskie
A_domestic_cat_Poland_H254_2023_H5N1_2023-06-22_Lublin
A_domestic_cat_Poland_H257-G_2023_H5N1_2023-06-24_Lublin
A_domestic_cat_Poland_H264-G_2023_H5N1_2023-06-24_Poznan
A_domestic_cat_Poland_H270-W_2023_H5N1_lubelskie
A_domestic_cat_Poland_Kot1_2023_H5N1_2023-06-19_Poznan
A_domestic_cat_Poland_H277-W1_2023_H5N1_2023-06-26_Namyslow_opolskie
A_white_stork_Poland_MB244_2023_H5N1_2023-06-04_tarnowski
A_domestic_cat_Poland_H271-W_2023_H5N1_lubelskie
A_domestic_cat_Poland_Kot2_2023_H5N1_2023-06-19_Poznan
A_domestic_cat_Poland_H256-G_2023_H5N1_2023-06-24_Lublin
A_domestic_cat_Poland_H266-W_2023_H5N1_2023-06-19_Bydgoszcz_kujawsko-pomorskie
A_domestic_cat_Poland_H249_2023_H5N1_2023-06-22_Gdansk
A_domestic_cat_Poland_H263-G_2023_H5N1_2023-06-24_Komarow-Osada
A_domestic_cat_Poland_H248_2023_H5N1_2023-06-15_Pruszcz_Gdanski
A_domestic_cat_Poland_H253_2023_H5N1_2023-06-22_Lublin
A_domestic_cat_Poland_H247_2023_H5N1_2023-06-20_Gdansk
A_cat_Poland_Gda1_2023_H5N1_2023-06-21
A_domestic_cat_Poland_H255-M_2023_H5N1_2023-06-21_Pruszcz_Gd
A_domestic_cat_Poland_H252_2023_H5N1_2023-06-22_Lublin
A_domestic_cat_Poland_H267-W_2023_H5N1_Strzelin_dolnoslaskie
A_mute_swan_Poland_MB107_2023_H5N1_2023-02-23
A_chicken_Slovakia_25-swab_23VIR1686-1_2023_H5N1_2023-01-12
A_chicken_Slovakia_25-brain_23VIR1686-3_2023_H5N1_2023-01-12
A_chicken_Slovakia_25-organs_23VIR1686-2_2023_H5N1_2023-01-12
A_swan_Czech_Republic_811-2_2023_H5N1_2023-01-16
A_domestic_duck_Poland_H429_2022_H5N1_2022-12-15
A_domestic_duck_Poland_H75-K1T1_2023_H5N1_2023-01-16
A_domestic_duck_Poland_H96-T6_2023_H5N1_2023-01-23
A_domestic_duck_Poland_H84-T1_2023_H5N1_2023-01-18
A_domestic_duck_Poland_H89-T1_2023_H5N1_2023-01-20
A_turkey_Poland_H90-K4T4_2023_H5N1_2023-01-21
A_domestic_duck_Poland_H70-T1_2023_H5N1_2023-01-16
A_domestic_duck_Poland_H73-T1_2023_H5N1_2023-01-16
A_domestic_duck_Poland_H74-T1_2023_H5N1_2023-01-16
A_domestic_duck_Poland_H86-T1_2023_H5N1_2023-01-18
A_domestic_duck_Czech_Republic_636_orig_2023_H5N1_2023-01-12
A_great_egret_Czech_Republic_3051-2orig_2023_H5N1_2023-02-21
A_great_egret_Czech_Republic_3051-1orig_2023_H5N1_2023-02-21
A_chicken_Czech_Republic_1690_orig_2023_H5N1_2023-01-30
A_chicken_Czech_Republic_1690_2023_H5N1_2023-01-30
A_domestic_duck_Poland_H485-N_2022_H5N1_2022-12-29
A_mute_swan_Austria_22155612_2022_H5N1_2022-12-28
A_chicken_Czech_Republic_23309-1orig_2022_H5N1_2022-12-15
A_mute_swan_Poland_MB113_2023_H5N1_2023-02-10
A_mallard_Czech_Republic_186_orig_2023_H5N1_2023-01-05
A_turkey_Romania_10281_23VIR1145-10_2023_H5N1_2023-01-27
A_corvus_corvus_Romania_10368_23VIR1368-1_2023_H5N1_2023-02-01
A_turkey_Romania_10287_23VIR1145-12_2023_H5N1_2023-01-28
A_turkey_Romania_10281_23VIR1145-11_2023_H5N1_2023-01-27
A_anas_platyrhynchos_Romania_10368_23VIR1368-2_2023_H5N1_2023-02-02
A_turkey_Romania_10271_23VIR1145-13_2023_H5N1_2023-01-26
A_mute_swan_Croatia_12_23VIR5604-19_2023_H5N1_2023-01-09
A_mute_swan_Croatia_12_23VIR5604-19_2023_H5N1_2023-01-09_2
A_mute_swan_Croatia_12-b_23VIR5604-20_2023_H5N1_2023-01-09_2_
A_mute_swan_Croatia_12-b_23VIR5604-20_2023_H5N1_2023-01-09
A_black_swan_Slovenia_137_23VIR1977-5_2023_H5N1_2023-02-02
A_grey_heron_Slovenia_163_23VIR1977-7_2023_H5N1_2023-02-06
A_mute_swan_Slovenia_241_23VIR1977-13_2023_H5N1_2023-02-21
A_mute_swan_Slovenia_242_23VIR1977-11_2023_H5N1_2023-02-23
A_domestic_duck_Poland_H433_2022_H5N1_2022-12-16
A_raven_Poland_MB035_2023_H5N1_2023-01-22
A_buzzard_Poland_MB275-TKL_2022_H5N1_2022-12-28
A_Eurasian_eagle-owl_Slovenia_142_23VIR1977-6_2023_H5N1_2023-02-02
A_domestic_duck_Poland_H470-K2T_2022_H5N1_2022-12-27
A_Gallus_gallus_Belgium_01688_0004_2023_H5N1_2023-02-23
CH
100
A_chicken_Czech_Republic_67_orig_2023_H5N1_2022-12-30
A_chicken_Czech_Republic_102_orig_2023_H5N1_2023-01-02
A_chicken_Czech_Republic_574_2023_H5N1_2023-01-11
A_chicken_Czech_Republic_574_orig_2023_H5N1_2023-01-11
A_chicken_Czech_Republic_727_orig_2023_H5N1_2023-01-15
A_European_herring_gull_Netherlands_9_2023_H5N1_2023-03-03
A_Eurasian_Wigeon_Netherlands_3_2022_H5N1_2022-10-21
A_common_tern_Poland_MB182-NJ_2023_H5N1_2023-05-10
A_black-headed_gull_Poland_MB166_2023_H5N1_2023-05-05
A_black-headed_gull_Poland_MB180-NMJ_2023_H5N1_2023-05-09
A_black-headed_gull_Poland_MB129-NJ_2023_H5N1_2023-03-22
A_black-headed_gull_Poland_MB131-J_2023_H5N1_2023-03-23
A_black-headed_gull_Poland_MB168-M2_2023_H5N1_2023-05-05
A_black-headed_gull_Poland_MB210_2023_H5N1_2023-05-16
A_Mediterranean_gull_Poland_MB204_2023_H5N1_2023-05-08
A_black-headed_gull_Poland_MB185-NJ_2023_H5N1_2023-05-10
A_black-headed_gull_Poland_MB209-NJ_2023_H5N1_2023
A_black-headed_gull_Poland_MB176-M6_2023_H5N1_2023-05-05
A_white_stork_Poland_MB274-M2_2023_H5N1_2023-07-07_malopolskie
A_white_stork_Poland_MB274-M3_2023_H5N1_2023-07-07_malopolskie
A_mute_swan_Poland_MB203-NJ_2023_H5N1_2023-05-11
A_black-headed_gull_Poland_MB141-T_2023_H5N1_2023-04-20
A_black-headed_gull_Poland_MB198-NJ_2023_H5N1_2023-05-11
A_black-headed_gull_Poland_MB081_2023_H5N1_2023-02-07
A_black-headed_gull_Poland_MB121-M_2023_H5N1_2023-03-13
A_black-headed_gull_Poland_MB211_2023_H5N1_2023-05-14
BB
0.03

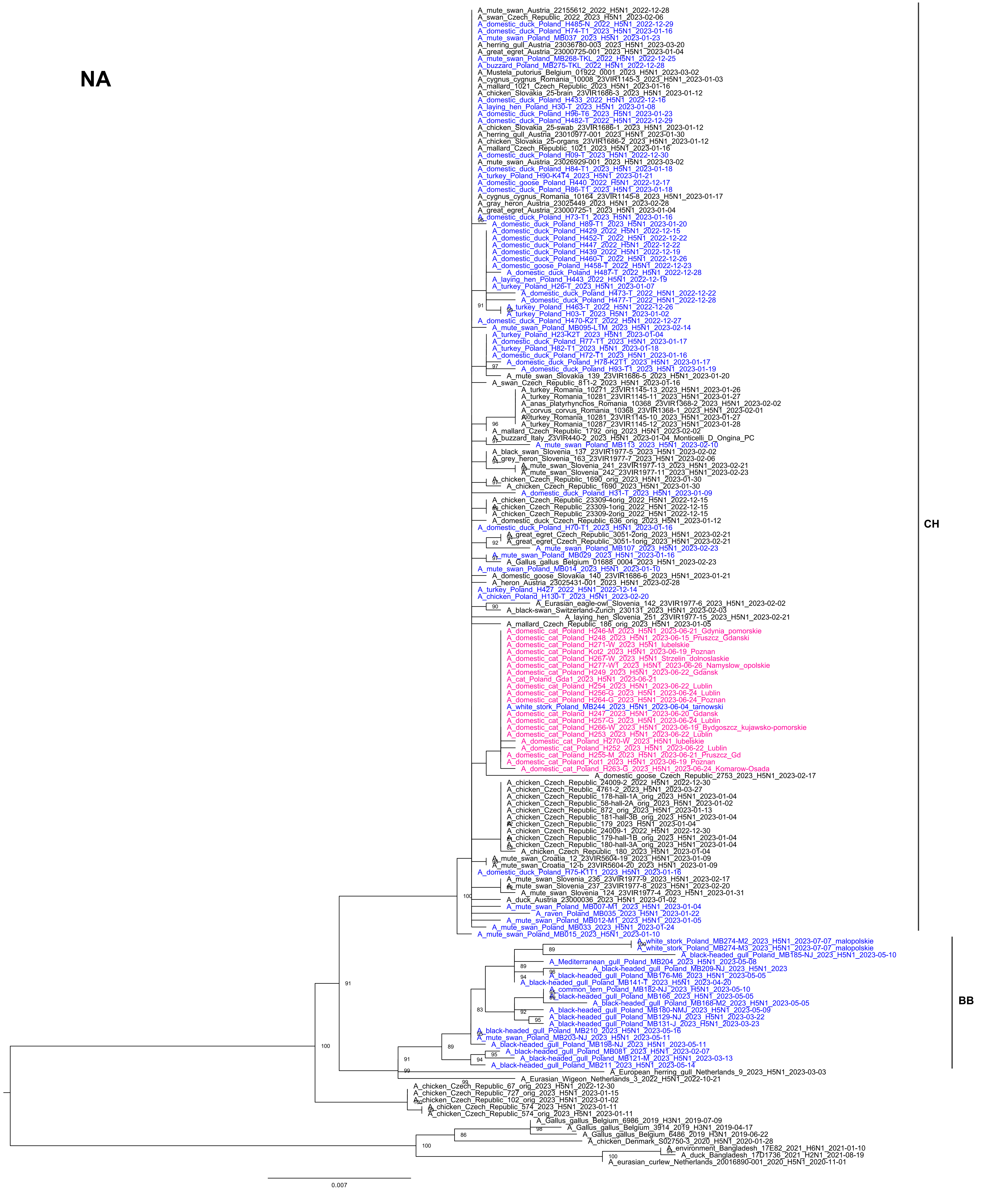
NA
A_mute_swan_Austria_22155612_2022_H5N1_2022-12-28
A_swan_Czech_Republic_2022_2023_H5N1_2023-02-06
A_domestic_duck_Poland_H485-N_2022_H5N1_2022-12-29
A_domestic_duck_Poland_H74-T1_2023_H5N1_2023-01-16
A_mute_swan_Poland_MB037_2023_H5N1_2023-01-23
A_herring_gull_Austria_23036780-003_2023_H5N1_2023-03-20
A_great_egret_Austria_23000725-001_2023_H5N1_2023-01-04
A_mute_swan_Poland_MB268-TKL_2022_H5N1_2022-12-25
A_buzzard_Poland_MB275-TKL_2022_H5N1_2022-12-28
A_Mustela_putorius_Belgium_01922_0001_2023_H5N1_2023-03-02
A_cygnus_cygnus_Romania_10008_23VIR1145-3_2023_H5N1_2023-01-03
A_mallard_1021_Czech_Republic_2023_H5N1_2023-01-16
A_chicken_Slovakia_25-brain_23VIR1686-3_2023_H5N1_2023-01-12
A_domestic_duck_Poland_H433_2022_H5N1_2022-12-16
A_laying_hen_Poland_H30-T_2023_H5N1_2023-01-08
A_domestic_duck_Poland_H96-T6_2023_H5N1_2023-01-23
A_domestic_duck_Poland_H482-T_2022_H5N1_2022-12-29
A_chicken_Slovakia_25-swab_23VIR1686-1_2023_H5N1_2023-01-12
A_herring_gull_Austria_23010977-001_2023_H5N1_2023-01-30
A_chicken_Slovakia_25-organs_23VIR1686-2_2023_H5N1_2023-01-12
A_mallard_Czech_Republic_1021_2023_H5N1_2023-01-16
A_domestic_duck_Poland_H09-T_2023_H5N1_2022-12-30
A_mute_swan_Austria_23026929-001_2023_H5N1_2023-03-02
A_domestic_duck_Poland_H84-T1_2023_H5N1_2023-01-18
A_turkey_Poland_H90-K4T4_2023_H5N1_2023-01-21
A_domestic_goose_Poland_H440_2022_H5N1_2022-12-17
A_domestic_duck_Poland_H86-T1_2023_H5N1_2023-01-18
A_cygnus_cygnus_Romania_10164_23VIR1145-8_2023_H5N1_2023-01-17
A_gray_heron_Austria_23025449_2023_H5N1_2023-02-28
A_great_egret_Austria_23000725-1_2023_H5N1_2023-01-04
A_domestic_duck_Poland_H73-T1_2023_H5N1_2023-01-16
A_domestic_duck_Poland_H89-T1_2023_H5N1_2023-01-20
A_domestic_duck_Poland_H429_2022_H5N1_2022-12-15
A_domestic_duck_Poland_H452-T_2022_H5N1_2022-12-22
A_domestic_duck_Poland_H447_2022_H5N1_2022-12-22
A_domestic_duck_Poland_H439_2022_H5N1_2022-12-19
A_domestic_duck_Poland_H460-T_2022_H5N1_2022-12-26
A_domestic_goose_Poland_H458-T_2022_H5N1_2022-12-23
A_domestic_duck_Poland_H487-T_2022_H5N1_2022-12-28
A_laying_hen_Poland_H443_2022_H5N1_2022-12-19
A_turkey_Poland_H26-T_2023_H5N1_2023-01-07
A_domestic_duck_Poland_H473-T_2022_H5N1_2022-12-22
A_domestic_duck_Poland_H477-T_2022_H5N1_2022-12-28
A_turkey_Poland_H463-T_2022_H5N1_2022-12-26
A_turkey_Poland_H03-T_2023_H5N1_2023-01-02
A_domestic_duck_Poland_H470-K2T_2022_H5N1_2022-12-27
A_mute_swan_Poland_MB095-LTM_2023_H5N1_2023-02-14
A_turkey_Poland_H23-K2T_2023_H5N1_2023-01-04
A_domestic_duck_Poland_H77-T1_2023_H5N1_2023-01-17
A_turkey_Poland_H82-T1_2023_H5N1_2023-01-18
A_domestic_duck_Poland_H72-T1_2023_H5N1_2023-01-16
A_domestic_duck_Poland_H78-K2T1_2023_H5N1_2023-01-17
A_domestic_duck_Poland_H93-T1_2023_H5N1_2023-01-19
A_mute_swan_Slovakia_139_23VIR1686-5_2023_H5N1_2023-01-20
A_swan_Czech_Republic_811-2_2023_H5N1_2023-01-16
A_turkey_Romania_10271_23VIR1145-13_2023_H5N1_2023-01-26
A_turkey_Romania_10281_23VIR1145-11_2023_H5N1_2023-01-27
A_anas_platyrhynchos_Romania_10368_23VIR1368-2_2023_H5N1_2023-02-02
A_corvus_corvus_Romania_10368_23VIR1368-1_2023_H5N1_2023-02-01
A_turkey_Romania_10281_23VIR1145-10_2023_H5N1_2023-01-27
A_turkey_Romania_10287_23VIR1145-12_2023_H5N1_2023-01-28
A_mallard_Czech_Republic_1792_orig_2023_H5N1_2023-02-02
A_buzzard_Italy_23VIR440-2_2023_H5N1_2023-01-04_Monticelli_D_Ongina_PC
A_mute_swan_Poland_MB113_2023_H5N1_2023-02-10
A_black_swan_Slovenia_137_23VIR1977-5_2023_H5N1_2023-02-02
A_grey_heron_Slovenia_163_23VIR1977-7_2023_H5N1_2023-02-06
A_mute_swan_Slovenia_241_23VIR1977-13_2023_H5N1_2023-02-21
A_mute_swan_Slovenia_242_23VIR1977-11_2023_H5N1_2023-02-23
A_chicken_Czech_Republic_1690_orig_2023_H5N1_2023-01-30
A_chicken_Czech_Republic_1690_2023_H5N1_2023-01-30
A_domestic_duck_Poland_H31-T_2023_H5N1_2023-01-09
A_chicken_Czech_Republic_23309-4orig_2022_H5N1_2022-12-15
A_chicken_Czech_Republic_23309-1orig_2022_H5N1_2022-12-15
A_chicken_Czech_Republic_23309-2orig_2022_H5N1_2022-12-15
A_domestic_duck_Czech_Republic_636_orig_2023_H5N1_2023-01-12
A_domestic_duck_Poland_H70-T1_2023_H5N1_2023-01-16
A_great_egret_Czech_Republic_3051-2orig_2023_H5N1_2023-02-21
A_great_egret_Czech_Republic_3051-1orig_2023_H5N1_2023-02-21
A_mute_swan_Poland_MB107_2023_H5N1_2023-02-23
A_mute_swan_Poland_MB029_2023_H5N1_2023-01-16
A_Gallus_gallus_Belgium_01688_0004_2023_H5N1_2023-02-23
A_mute_swan_Poland_MB014_2023_H5N1_2023-01-10
A_domestic_goose_Slovakia_140_23VIR1686-6_2023_H5N1_2023-01-21
A_heron_Austria_23025431-001_2023_H5N1_2023-02-28
A_turkey_Poland_H427_2022_H5N1_2022-12-14
A_chicken_Poland_H130-T_2023_H5N1_2023-02-20
A_Eurasian_eagle-owl_Slovenia_142_23VIR1977-6_2023_H5N1_2023-02-02
A_black-swan_Switzerland-Zurich_230131_2023_H5N1_2023-02-03
A_laying_hen_Slovenia_251_23VIR1977-15_2023_H5N1_2023-02-21
A_mallard_Czech_Republic_186_orig_2023_H5N1_2023-01-05
A_domestic_cat_Poland_H246-M_2023_H5N1_2023-06-21_Gdynia_pomorskie
A_domestic_cat_Poland_H248_2023_H5N1_2023-06-15_Pruszcz_Gdanski
A_domestic_cat_Poland_H271-W_2023_H5N1_lubelskie
A_domestic_cat_Poland_Kot2_2023_H5N1_2023-06-19_Poznan
A_domestic_cat_Poland_H267-W_2023_H5N1_Strzelin_dolnoslaskie
A_domestic_cat_Poland_H277-WT_2023_H5N1_2023-06-26_Namyslow_opolskie
A_domestic_cat_Poland_H249_2023_H5N1_2023-06-22_Gdansk
A_cat_Poland_Gda1_2023_H5N1_2023-06-21
A_domestic_cat_Poland_H254_2023_H5N1_2023-06-22_Lublin
A_domestic_cat_Poland_H256-G_2023_H5N1_2023-06-24_Lublin
A_domestic_cat_Poland_H264-G_2023_H5N1_2023-06-24_Poznan
A_white_stork_Poland_MB244_2023_H5N1_2023-06-04_tarnowski
A_domestic_cat_Poland_H247_2023_H5N1_2023-06-20_Gdansk
A_domestic_cat_Poland_H257-G_2023_H5N1_2023-06-24_Lublin
A_domestic_cat_Poland_H266-W_2023_H5N1_2023-06-19_Bydgoszcz_kujawsko-pomorskie
A_domestic_cat_Poland_H253_2023_H5N1_2023-06-22_Lublin
A_domestic_cat_Poland_H270-W_2023_H5N1_lubelskie
A_domestic_cat_Poland_H252_2023_H5N1_2023-06-22_Lublin
A_domestic_cat_Poland_H255-M_2023_H5N1_2023-06-21_Pruszcz_Gd
A_domestic_cat_Poland_Kot1_2023_H5N1_2023-06-19_Poznan
A_domestic_cat_Poland_H263-G_2023_H5N1_2023-06-24_Komarow-Osada
A_domestic_goose_Czech_Republic_2753_2023_H5N1_2023-02-17
A_chicken_Czech_Republic_24009-2_2022_H5N1_2022-12-30
A_chicken_Czech_Reublic_4761-2_2023_H5N1_2023-03-27
A_chicken_Czech_Republic_178-hall-1A_orig_2023_H5N1_2023-01-04
A_chicken_Czech_Republic_58-hall-2A_orig_2023_H5N1_2023-01-02
A_chicken_Czech_Republic_872_orig_2023_H5N1_2023-01-13
A_chicken_Czech_Republic_181-hall-3B_orig_2023_H5N1_2023-01-04
A_chicken_Czech_Republic_179_2023_H5N1_2023-01-04
A_chicken_Czech_Republic_24009-1_2022_H5N1_2022-12-30
A_chicken_Czech_Republic_179-hall-1B_orig_2023_H5N1_2023-01-04
A_chicken_Czech_Republic_180-hall-3A_orig_2023_H5N1_2023-01-04
A_chicken_Czech_Republic_180_2023_H5N1_2023-01-04
A_mute_swan_Croatia_12_23VIR5604-19_2023_H5N1_2023-01-09
A_mute_swan_Croatia_12-b_23VIR5604-20_2023_H5N1_2023-01-09
A_domestic_duck_Poland_H75-K1T1_2023_H5N1_2023-01-16
A_mute_swan_Slovenia_236_23VIR1977-9_2023_H5N1_2023-02-17
A_mute_swan_Slovenia_237_23VIR1977-8_2023_H5N1_2023-02-20
A_mute_swan_Slovenia_124_23VIR1977-4_2023_H5N1_2023-01-31
A_duck_Austria_23000036_2023_H5N1_2023-01-02
A_mute_swan_Poland_MB007-M1_2023_H5N1_2023-01-04
A_raven_Poland_MB035_2023_H5N1_2023-01-22
A_mute_swan_Poland_MB012-M1_2023_H5N1_2023-01-05
A_mute_swan_Poland_MB033_2023_H5N1_2023-01-24
A_mute_swan_Poland_MB015_2023_H5N1_2023-01-10
A_white_stork_Poland_MB274-M2_2023_H5N1_2023-07-07_malopolskie
A_white_stork_Poland_MB274-M3_2023_H5N1_2023-07-07_malopolskie
A_black-headed_gull_Poland_MB185-NJ_2023_H5N1_2023-05-10
A_Mediterranean_gull_Poland_MB204_2023_H5N1_2023-05-08
A_black-headed_gull_Poland_MB209-NJ_2023_H5N1_2023
A_black-headed_gull_Poland_MB176-M6_2023_H5N1_2023-05-05
A_black-headed_gull_Poland_MB141-T_2023_H5N1_2023-04-20
A_common_tern_Poland_MB182-NJ_2023_H5N1_2023-05-10
A_black-headed_gull_Poland_MB166_2023_H5N1_2023-05-05
A_black-headed_gull_Poland_MB168-M2_2023_H5N1_2023-05-05
A_black-headed_gull_Poland_MB180-NMJ_2023_H5N1_2023-05-09
A_black-headed_gull_Poland_MB129-NJ_2023_H5N1_2023-03-22
A_black-headed_gull_Poland_MB131-J_2023_H5N1_2023-03-23
A_black-headed_gull_Poland_MB210_2023_H5N1_2023-05-16
A_mute_swan_Poland_MB203-NJ_2023_H5N1_2023-05-11
A_black-headed_gull_Poland_MB198-NJ_2023_H5N1_2023-05-11
A_black-headed_gull_Poland_MB081_2023_H5N1_2023-02-07
A_black-headed_gull_Poland_MB121-M_2023_H5N1_2023-03-13
A_black-headed_gull_Poland_MB211_2023_H5N1_2023-05-14
A_European_herring_gull_Netherlands_9_2023_H5N1_2023-03-03
A_Eurasian_Wigeon_Netherlands_3_2022_H5N1_2022-10-21
A_chicken_Czech_Republic_67_orig_2023_H5N1_2022-12-30
A_chicken_Czech_Republic_727_orig_2023_H5N1_2023-01-15
A_chicken_Czech_Republic_102_orig_2023_H5N1_2023-01-02
A_chicken_Czech_Republic_574_2023_H5N1_2023-01-11
A_chicken_Czech_Republic_574_orig_2023_H5N1_2023-01-11
A_Gallus_gallus_Belgium_6986_2019_H3N1_2019-07-09
A_Gallus_gallus_Belgium_3914_2019_H3N1_2019-04-17
A_Gallus_gallus_Belgium_6486_2019_H3N1_2019-06-22
A_chicken_Denmark_S02750-3_2020_H5N1_2020-01-28
A_environment_Bangladesh_17E82_2021_H6N1_2021-01-10
A_duck_Bangladesh_17D1736_2021_H2N1_2021-08-19
A_eurasian_curlew_Netherlands_20016890-001_2020_H5N1_2020-11-01
CH
BB
0.007

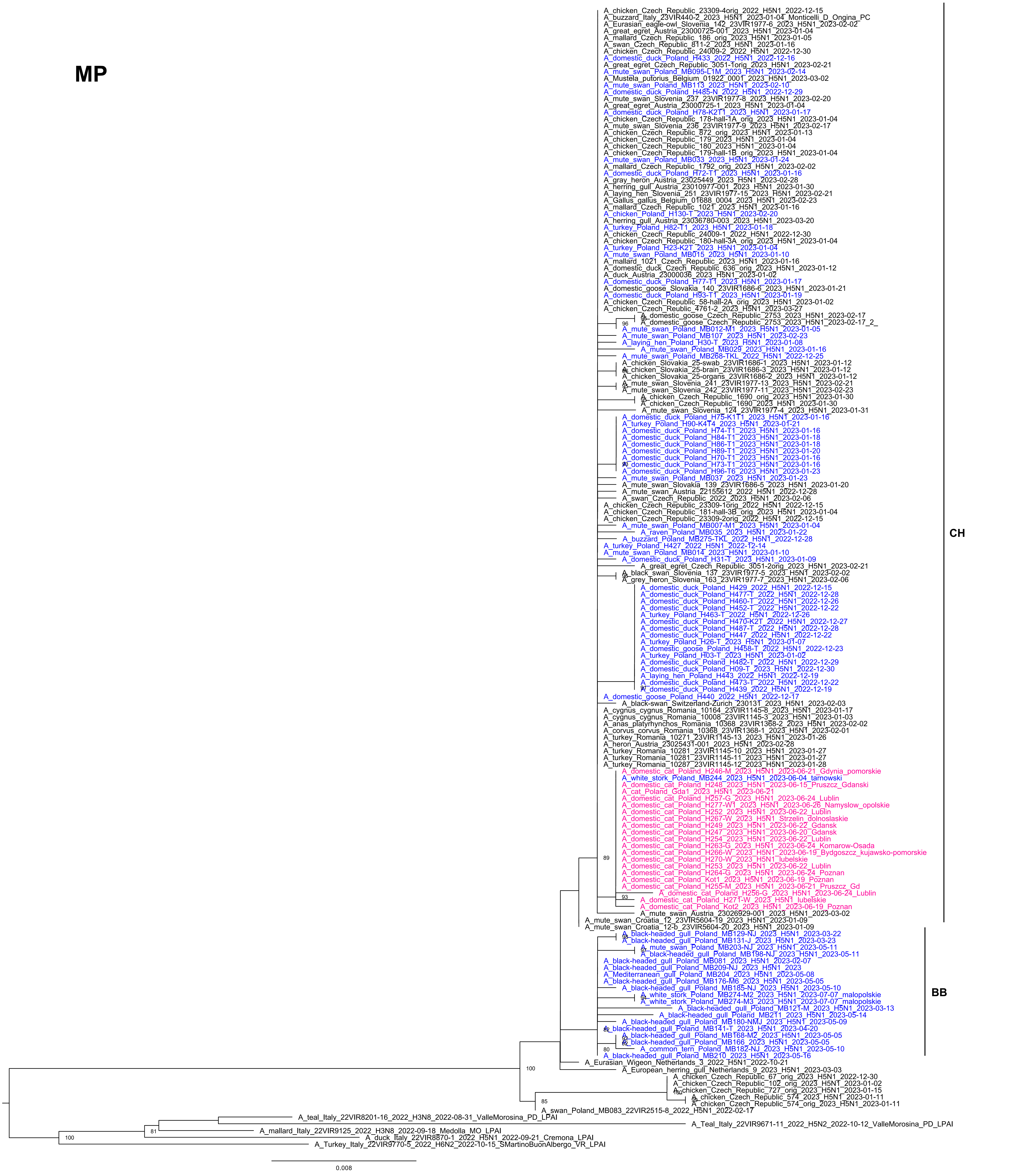

MP
A_chicken_Czech_Republic_23309-4orig_2022_H5N1_2022-12-15
A_buzzard_Italy_23VIR440-2_2023_H5N1_2023-01-04_Monticelli_D_Ongina_PC
A_Eurasian_eagle-owl_Slovenia_142_23VIR1977-6_2023_H5N1_2023-02-02
A_great_egret_Austria_23000725-001_2023_H5N1_2023-01-04
A_mallard_Czech_Republic_186_orig_2023_H5N1_2023-01-05
A_swan_Czech_Republic_811-2_2023_H5N1_2023-01-16
A_chicken_Czech_Republic_24009-2_2022_H5N1_2022-12-30
A_domestic_duck_Poland_H433_2022_H5N1_2022-12-16
A_great_egret_Czech_Republic_3051-1orig_2023_H5N1_2023-02-21
A_mute_swan_Poland_MB095-L1M_2023_H5N1_2023-02-14
A_Mustela_putorius_Belgium_01922_0001_2023_H5N1_2023-03-02
A_mute_swan_Poland_MB113_2023_H5N1_2023-02-10
A_domestic_duck_Poland_H485-N_2022_H5N1_2022-12-29
A_mute_swan_Slovenia_237_23VIR1977-8_2023_H5N1_2023-02-20
A_great_egret_Austria_23000725-1_2023_H5N1_2023-01-04
A_domestic_duck_Poland_H78-K2T1_2023_H5N1_2023-01-17
A_chicken_Czech_Republic_178-hall-1A_orig_2023_H5N1_2023-01-04
A_mute_swan_Slovenia_236_23VIR1977-9_2023_H5N1_2023-02-17
A_chicken_Czech_Republic_872_orig_2023_H5N1_2023-01-13
A_chicken_Czech_Republic_179_2023_H5N1_2023-01-04
A_chicken_Czech_Republic_180_2023_H5N1_2023-01-04
A_chicken_Czech_Republic_179-hall-1B_orig_2023_H5N1_2023-01-04
A_mute_swan_Poland_MB033_2023_H5N1_2023-01-24
A_mallard_Czech_Republic_1792_orig_2023_H5N1_2023-02-02
A_domestic_duck_Poland_H72-T1_2023_H5N1_2023-01-16
A_gray_heron_Austria_23025449_2023_H5N1_2023-02-28
A_herring_gull_Austria_23010977-001_2023_H5N1_2023-01-30
A_laying_hen_Slovenia_251_23VIR1977-15_2023_H5N1_2023-02-21
A_Gallus_gallus_Belgium_01688_0004_2023_H5N1_2023-02-23
A_mallard_Czech_Republic_1021_2023_H5N1_2023-01-16
A_chicken_Poland_H130-T_2023_H5N1_2023-02-20
A_herring_gull_Austria_23036780-003_2023_H5N1_2023-03-20
A_turkey_Poland_H82-T1_2023_H5N1_2023-01-18
A_chicken_Czech_Republic_24009-1_2022_H5N1_2022-12-30
A_chicken_Czech_Republic_180-hall-3A_orig_2023_H5N1_2023-01-04
A_turkey_Poland_H23-K2T_2023_H5N1_2023-01-04
A_mute_swan_Poland_MB015_2023_H5N1_2023-01-10
A_mallard_1021_Czech_Republic_2023_H5N1_2023-01-16
A_domestic_duck_Czech_Republic_636_orig_2023_H5N1_2023-01-12
A_duck_Austria_23000036_2023_H5N1_2023-01-02
A_domestic_duck_Poland_H77-T1_2023_H5N1_2023-01-17
A_domestic_goose_Slovakia_140_23VIR1686-6_2023_H5N1_2023-01-21
A_domestic_duck_Poland_H93-T1_2023_H5N1_2023-01-19
A_chicken_Czech_Republic_58-hall-2A_orig_2023_H5N1_2023-01-02
A_chicken_Czech_Reublic_4761-2_2023_H5N1_2023-03-27
A_domestic_goose_Czech_Republic_2753_2023_H5N1_2023-02-17
96
A_domestic_goose_Czech_Republic_2753_2023_H5N1_2023-02-17_2_
A_mute_swan_Poland_MB012-M1_2023_H5N1_2023-01-05
A_mute_swan_Poland_MB107_2023_H5N1_2023-02-23
A_laying_hen_Poland_H30-T_2023_H5N1_2023-01-08
A_mute_swan_Poland_MB029_2023_H5N1_2023-01-16
A_mute_swan_Poland_MB268-TKL_2022_H5N1_2022-12-25
A_chicken_Slovakia_25-swab_23VIR1686-1_2023_H5N1_2023-01-12
A_chicken_Slovakia_25-brain_23VIR1686-3_2023_H5N1_2023-01-12
A_chicken_Slovakia_25-organs_23VIR1686-2_2023_H5N1_2023-01-12
A_mute_swan_Slovenia_241_23VIR1977-13_2023_H5N1_2023-02-21
A_mute_swan_Slovenia_242_23VIR1977-11_2023_H5N1_2023-02-23
A_chicken_Czech_Republic_1690_orig_2023_H5N1_2023-01-30
A_chicken_Czech_Republic_1690_2023_H5N1_2023-01-30
A_mute_swan_Slovenia_124_23VIR1977-4_2023_H5N1_2023-01-31
A_domestic_duck_Poland_H75-K1T1_2023_H5N1_2023-01-16
A_turkey_Poland_H90-K4T4_2023_H5N1_2023-01-21
A_domestic_duck_Poland_H74-T1_2023_H5N1_2023-01-16
A_domestic_duck_Poland_H84-T1_2023_H5N1_2023-01-18
A_domestic_duck_Poland_H86-T1_2023_H5N1_2023-01-18
A_domestic_duck_Poland_H89-T1_2023_H5N1_2023-01-20
A_domestic_duck_Poland_H70-T1_2023_H5N1_2023-01-16
A_domestic_duck_Poland_H73-T1_2023_H5N1_2023-01-16
A_domestic_duck_Poland_H96-T6_2023_H5N1_2023-01-23
A_mute_swan_Poland_MB037_2023_H5N1_2023-01-23
A_mute_swan_Slovakia_139_23VIR1686-5_2023_H5N1_2023-01-20
A_mute_swan_Austria_22155612_2022_H5N1_2022-12-28
A_swan_Czech_Republic_2022_2023_H5N1_2023-02-06
A_chicken_Czech_Republic_23309-1orig_2022_H5N1_2022-12-15
A_chicken_Czech_Republic_181-hall-3B_orig_2023_H5N1_2023-01-04
A_chicken_Czech_Republic_23309-2orig_2022_H5N1_2022-12-15
A_mute_swan_Poland_MB007-M1_2023_H5N1_2023-01-04
A_raven_Poland_MB035_2023_H5N1_2023-01-22
A_buzzard_Poland_MB275-TKL_2022_H5N1_2022-12-28
A_turkey_Poland_H427_2022_H5N1_2022-12-14
A_mute_swan_Poland_MB014_2023_H5N1_2023-01-10
A_domestic_duck_Poland_H31-T_2023_H5N1_2023-01-09
A_great_egret_Czech_Republic_3051-2orig_2023_H5N1_2023-02-21
A_black_swan_Slovenia_137_23VIR1977-5_2023_H5N1_2023-02-02
A_grey_heron_Slovenia_163_23VIR1977-7_2023_H5N1_2023-02-06
A_domestic_duck_Poland_H429_2022_H5N1_2022-12-15
A_domestic_duck_Poland_H477-T_2022_H5N1_2022-12-28
A_domestic_duck_Poland_H460-T_2022_H5N1_2022-12-26
A_domestic_duck_Poland_H452-T_2022_H5N1_2022-12-22
A_turkey_Poland_H463-T_2022_H5N1_2022-12-26
A_domestic_duck_Poland_H470-K2T_2022_H5N1_2022-12-27
A_domestic_duck_Poland_H487-T_2022_H5N1_2022-12-28
A_domestic_duck_Poland_H447_2022_H5N1_2022-12-22
A_turkey_Poland_H26-T_2023_H5N1_2023-01-07
A_domestic_goose_Poland_H458-T_2022_H5N1_2022-12-23
A_turkey_Poland_H03-T_2023_H5N1_2023-01-02
A_domestic_duck_Poland_H482-T_2022_H5N1_2022-12-29
A_domestic_duck_Poland_H09-T_2023_H5N1_2022-12-30
A_laying_hen_Poland_H443_2022_H5N1_2022-12-19
A_domestic_duck_Poland_H473-T_2022_H5N1_2022-12-22
A_domestic_duck_Poland_H439_2022_H5N1_2022-12-19
A_domestic_goose_Poland_H440_2022_H5N1_2022-12-17
A_black-swan_Switzerland-Zurich_23013T_2023_H5N1_2023-02-03
A_cygnus_cygnus_Romania_10164_23VIR1145-8_2023_H5N1_2023-01-17
A_cygnus_cygnus_Romania_10008_23VIR1145-3_2023_H5N1_2023-01-03
A_anas_platyrhynchos_Romania_10368_23VIR1368-2_2023_H5N1_2023-02-02
A_corvus_corvus_Romania_10368_23VIR1368-1_2023_H5N1_2023-02-01
A_turkey_Romania_10271_23VIR1145-13_2023_H5N1_2023-01-26
A_heron_Austria_23025431-001_2023_H5N1_2023-02-28
A_turkey_Romania_10281_23VIR1145-10_2023_H5N1_2023-01-27
A_turkey_Romania_10281_23VIR1145-11_2023_H5N1_2023-01-27
A_turkey_Romania_10287_23VIR1145-12_2023_H5N1_2023-01-28
A_domestic_cat_Poland_H246-M_2023_H5N1_2023-06-21_Gdynia_pomorskie
A_white_stork_Poland_MB244_2023_H5N1_2023-06-04_tarnowski
A_domestic_cat_Poland_H248_2023_H5N1_2023-06-15_Pruszcz_Gdanski
A_cat_Poland_Gda1_2023_H5N1_2023-06-21
A_domestic_cat_Poland_H257-G_2023_H5N1_2023-06-24_Lublin
A_domestic_cat_Poland_H277-W1_2023_H5N1_2023-06-26_Namyslow_opolskie
A_domestic_cat_Poland_H252_2023_H5N1_2023-06-22_Lublin
A_domestic_cat_Poland_H267-W_2023_H5N1_Strzelin_dolnoslaskie
A_domestic_cat_Poland_H249_2023_H5N1_2023-06-22_Gdansk
A_domestic_cat_Poland_H247_2023_H5N1_2023-06-20_Gdansk
A_domestic_cat_Poland_H254_2023_H5N1_2023-06-22_Lublin
A_domestic_cat_Poland_H263-G_2023_H5N1_2023-06-24_Komarow-Osada
89
A_domestic_cat_Poland_H266-W_2023_H5N1_2023-06-19_Bydgoszcz_kujawsko-pomorskie
A_domestic_cat_Poland_H270-W_2023_H5N1_lubelskie
A_domestic_cat_Poland_H253_2023_H5N1_2023-06-22_Lublin
A_domestic_cat_Poland_H264-G_2023_H5N1_2023-06-24_Poznan
A_domestic_cat_Poland_Kot1_2023_H5N1_2023-06-19_Poznan
A_domestic_cat_Poland_H255-M_2023_H5N1_2023-06-21_Pruszcz_Gd
A_domestic_cat_Poland_H256-G_2023_H5N1_2023-06-24_Lublin
93
A_domestic_cat_Poland_H271-W_2023_H5N1_lubelskie
A_domestic_cat_Poland_Kot2_2023_H5N1_2023-06-19_Poznan
A_mute_swan_Austria_23026929-001_2023_H5N1_2023-03-02
A_mute_swan_Croatia_12_23VIR5604-19_2023_H5N1_2023-01-09
A_mute_swan_Croatia_12-b_23VIR5604-20_2023_H5N1_2023-01-09
CH
A_black-headed_gull_Poland_MB129-NJ_2023_H5N1_2023-03-22
A_black-headed_gull_Poland_MB131-J_2023_H5N1_2023-03-23
A_mute_swan_Poland_MB203-NJ_2023_H5N1_2023-05-11
A_black-headed_gull_Poland_MB198-NJ_2023_H5N1_2023-05-11
A_black-headed_gull_Poland_MB081_2023_H5N1_2023-02-07
A_black-headed_gull_Poland_MB209-NJ_2023_H5N1_2023
A_Mediterranean_gull_Poland_MB204_2023_H5N1_2023-05-08
A_black-headed_gull_Poland_MB176-M6_2023_H5N1_2023-05-05
A_black-headed_gull_Poland_MB185-NJ_2023_H5N1_2023-05-10
A_white_stork_Poland_MB274-M2_2023_H5N1_2023-07-07_malopolskie
A_white_stork_Poland_MB274-M3_2023_H5N1_2023-07-07_malopolskie
A_black-headed_gull_Poland_MB121-M_2023_H5N1_2023-03-13
A_black-headed_gull_Poland_MB211_2023_H5N1_2023-05-14
A_black-headed_gull_Poland_MB180-NMJ_2023_H5N1_2023-05-09
A_black-headed_gull_Poland_MB141-T_2023_H5N1_2023-04-20
A_black-headed_gull_Poland_MB168-M2_2023_H5N1_2023-05-05
A_black-headed_gull_Poland_MB166_2023_H5N1_2023-05-05
80
A_common_tern_Poland_MB182-NJ_2023_H5N1_2023-05-10
A_black-headed_gull_Poland_MB210_2023_H5N1_2023-05-16
BB
A_Eurasian_Wigeon_Netherlands_3_2022_H5N1_2022-10-21
100
A_European_herring_gull_Netherlands_9_2023_H5N1_2023-03-03
A_chicken_Czech_Republic_67_orig_2023_H5N1_2022-12-30
A_chicken_Czech_Republic_102_orig_2023_H5N1_2023-01-02
A_chicken_Czech_Republic_727_orig_2023_H5N1_2023-01-15
A_chicken_Czech_Republic_574_2023_H5N1_2023-01-11
A_chicken_Czech_Republic_574_orig_2023_H5N1_2023-01-11
85
A_swan_Poland_MB083_22VIR2515-8_2022_H5N1_2022-02-17
A_teal_Italy_22VIR8201-16_2022_H3N8_2022-08-31_ValleMorosina_PD_LPAI
A_Teal_Italy_22VIR9671-11_2022_H5N2_2022-10-12_ValleMorosina_PD_LPAI
81
A_mallard_Italy_22VIR9125_2022_H3N8_2022-09-18_Medolla_MO_LPAI
100
A_duck_Italy_22VIR8870-1_2022_H5N1_2022-09-21_Cremona_LPAI
A_Turkey_Italy_22VIR9770-5_2022_H6N2_2022-10-15_SMartinoBuonAlbergo_VR_LPAI
0.008

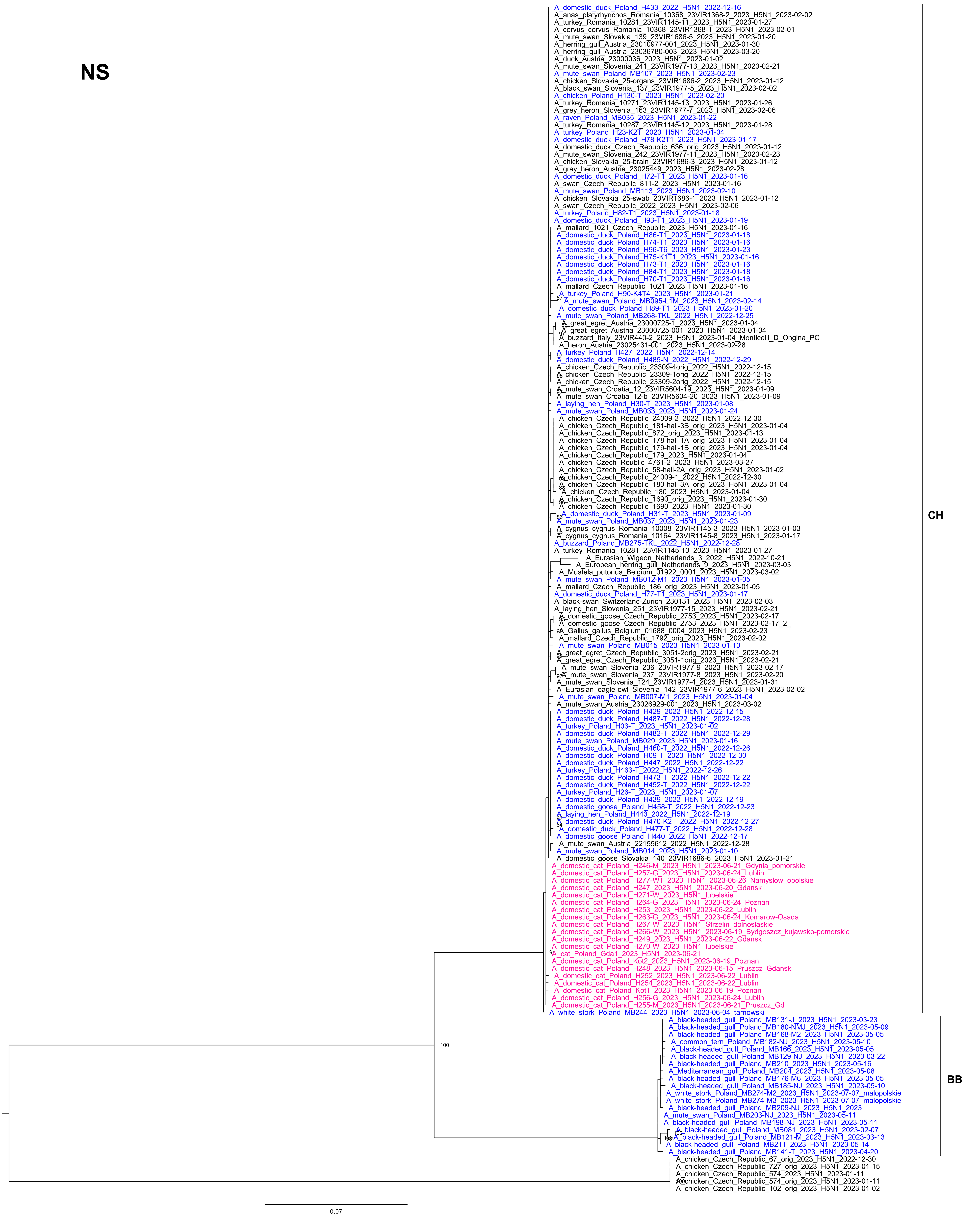
NS
A_domestic_duck_Poland_H433_2022_H5N1_2022-12-16
A_anas_platyrhynchos_Romania_10368_23VIR1368-2_2023_H5N1_2023-02-02
A_turkey_Romania_10281_23VIR1145-11_2023_H5N1_2023-01-27
A_corvus_corvus_Romania_10368_23VIR1368-1_2023_H5N1_2023-02-01
A_mute_swan_Slovakia_139_23VIR1686-5_2023_H5N1_2023-01-20
A_herring_gull_Austria_23010977-001_2023_H5N1_2023-01-30
A_herring_gull_Austria_23036780-003_2023_H5N1_2023-03-20
A_duck_Austria_23000036_2023_H5N1_2023-01-02
A_mute_swan_Slovenia_241_23VIR1977-13_2023_H5N1_2023-02-21
A_mute_swan_Poland_MB107_2023_H5N1_2023-02-23
A_chicken_Slovakia_25-organs_23VIR1686-2_2023_H5N1_2023-01-12
A_black_swan_Slovenia_137_23VIR1977-5_2023_H5N1_2023-02-02
A_chicken_Poland_H130-T_2023_H5N1_2023-02-20
A_turkey_Romania_10271_23VIR1145-13_2023_H5N1_2023-01-26
A_grey_heron_Slovenia_163_23VIR1977-7_2023_H5N1_2023-02-06
A_raven_Poland_MB035_2023_H5N1_2023-01-22
A_turkey_Romania_10287_23VIR1145-12_2023_H5N1_2023-01-28
A_turkey_Poland_H23-K2T_2023_H5N1_2023-01-04
A_domestic_duck_Poland_H78-K2T1_2023_H5N1_2023-01-17
A_domestic_duck_Czech_Republic_636_orig_2023_H5N1_2023-01-12
A_mute_swan_Slovenia_242_23VIR1977-11_2023_H5N1_2023-02-23
A_chicken_Slovakia_25-brain_23VIR1686-3_2023_H5N1_2023-01-12
A_gray_heron_Austria_23025449_2023_H5N1_2023-02-28
A_domestic_duck_Poland_H72-T1_2023_H5N1_2023-01-16
A_swan_Czech_Republic_811-2_2023_H5N1_2023-01-16
A_mute_swan_Poland_MB113_2023_H5N1_2023-02-10
A_chicken_Slovakia_25-swab_23VIR1686-1_2023_H5N1_2023-01-12
A_swan_Czech_Republic_2022_2023_H5N1_2023-02-06
A_turkey_Poland_H82-T1_2023_H5N1_2023-01-18
A_domestic_duck_Poland_H93-T1_2023_H5N1_2023-01-19
A_mallard_1021_Czech_Republic_2023_H5N1_2023-01-16
A_domestic_duck_Poland_H86-T1_2023_H5N1_2023-01-18
A_domestic_duck_Poland_H74-T1_2023_H5N1_2023-01-16
A_domestic_duck_Poland_H96-T6_2023_H5N1_2023-01-23
A_domestic_duck_Poland_H75-K1T1_2023_H5N1_2023-01-16
A_domestic_duck_Poland_H73-T1_2023_H5N1_2023-01-16
A_domestic_duck_Poland_H84-T1_2023_H5N1_2023-01-18
A_domestic_duck_Poland_H70-T1_2023_H5N1_2023-01-16
A_mallard_Czech_Republic_1021_2023_H5N1_2023-01-16
A_turkey_Poland_H90-K4T4_2023_H5N1_2023-01-21
87
A_mute_swan_Poland_MB095-L1M_2023_H5N1_2023-02-14
A_domestic_duck_Poland_H89-T1_2023_H5N1_2023-01-20
A_mute_swan_Poland_MB268-TKL_2022_H5N1_2022-12-25
A_great_egret_Austria_23000725-1_2023_H5N1_2023-01-04
A_great_egret_Austria_23000725-001_2023_H5N1_2023-01-04
A_buzzard_Italy_23VIR440-2_2023_H5N1_2023-01-04_Monticelli_D_Ongina_PC
A_heron_Austria_23025431-001_2023_H5N1_2023-02-28
A_turkey_Poland_H427_2022_H5N1_2022-12-14
A_domestic_duck_Poland_H485-N_2022_H5N1_2022-12-29
A_chicken_Czech_Republic_23309-4orig_2022_H5N1_2022-12-15
A_chicken_Czech_Republic_23309-1orig_2022_H5N1_2022-12-15
A_chicken_Czech_Republic_23309-2orig_2022_H5N1_2022-12-15
A_mute_swan_Croatia_12_23VIR5604-19_2023_H5N1_2023-01-09
A_mute_swan_Croatia_12-b_23VIR5604-20_2023_H5N1_2023-01-09
A_laying_hen_Poland_H30-T_2023_H5N1_2023-01-08
A_mute_swan_Poland_MB033_2023_H5N1_2023-01-24
A_chicken_Czech_Republic_24009-2_2022_H5N1_2022-12-30
A_chicken_Czech_Republic_181-hall-3B_orig_2023_H5N1_2023-01-04
A_chicken_Czech_Republic_872_orig_2023_H5N1_2023-01-13
A_chicken_Czech_Republic_178-hall-1A_orig_2023_H5N1_2023-01-04
A_chicken_Czech_Republic_179-hall-1B_orig_2023_H5N1_2023-01-04
A_chicken_Czech_Republic_179_2023_H5N1_2023-01-04
A_chicken_Czech_Reublic_4761-2_2023_H5N1_2023-03-27
A_chicken_Czech_Republic_58-hall-2A_orig_2023_H5N1_2023-01-02
A_chicken_Czech_Republic_24009-1_2022_H5N1_2022-12-30
A_chicken_Czech_Republic_180-hall-3A_orig_2023_H5N1_2023-01-04
A_chicken_Czech_Republic_180_2023_H5N1_2023-01-04
A_chicken_Czech_Republic_1690_orig_2023_H5N1_2023-01-30
A_chicken_Czech_Republic_1690_2023_H5N1_2023-01-30
A_domestic_duck_Poland_H31-T_2023_H5N1_2023-01-09
86
A_mute_swan_Poland_MB037_2023_H5N1_2023-01-23
A_cygnus_cygnus_Romania_10008_23VIR1145-3_2023_H5N1_2023-01-03
A_cygnus_cygnus_Romania_10164_23VIR1145-8_2023_H5N1_2023-01-17
A_buzzard_Poland_MB275-TKL_2022_H5N1_2022-12-28
A_turkey_Romania_10281_23VIR1145-10_2023_H5N1_2023-01-27
A_Eurasian_Wigeon_Netherlands_3_2022_H5N1_2022-10-21
A_European_herring_gull_Netherlands_9_2023_H5N1_2023-03-03
A_Mustela_putorius_Belgium_01922_0001_2023_H5N1_2023-03-02
A_mute_swan_Poland_MB012-M1_2023_H5N1_2023-01-05
A_mallard_Czech_Republic_186_orig_2023_H5N1_2023-01-05
A_domestic_duck_Poland_H77-T1_2023_H5N1_2023-01-17
A_black-swan_Switzerland-Zurich_230131_2023_H5N1_2023-02-03
A_laying_hen_Slovenia_251_23VIR1977-15_2023_H5N1_2023-02-21
A_domestic_goose_Czech_Republic_2753_2023_H5N1_2023-02-17
A_domestic_goose_Czech_Republic_2753_2023_H5N1_2023-02-17_2_
A_Gallus_gallus_Belgium_01688_0004_2023_H5N1_2023-02-23
A_mallard_Czech_Republic_1792_orig_2023_H5N1_2023-02-02
A_mute_swan_Poland_MB015_2023_H5N1_2023-01-10
A_great_egret_Czech_Republic_3051-2orig_2023_H5N1_2023-02-21
A_great_egret_Czech_Republic_3051-1orig_2023_H5N1_2023-02-21
A_mute_swan_Slovenia_236_23VIR1977-9_2023_H5N1_2023-02-17
97
A_mute_swan_Slovenia_237_23VIR1977-8_2023_H5N1_2023-02-20
A_mute_swan_Slovenia_124_23VIR1977-4_2023_H5N1_2023-01-31
A_Eurasian_eagle-owl_Slovenia_142_23VIR1977-6_2023_H5N1_2023-02-02
A_mute_swan_Poland_MB007-M1_2023_H5N1_2023-01-04
A_mute_swan_Austria_23026929-001_2023_H5N1_2023-03-02
A_domestic_duck_Poland_H429_2022_H5N1_2022-12-15
A_domestic_duck_Poland_H487-T_2022_H5N1_2022-12-28
A_turkey_Poland_H03-T_2023_H5N1_2023-01-02
A_domestic_duck_Poland_H482-T_2022_H5N1_2022-12-29
A_mute_swan_Poland_MB029_2023_H5N1_2023-01-16
A_domestic_duck_Poland_H460-T_2022_H5N1_2022-12-26
A_domestic_duck_Poland_H09-T_2023_H5N1_2022-12-30
A_domestic_duck_Poland_H447_2022_H5N1_2022-12-22
A_turkey_Poland_H463-T_2022_H5N1_2022-12-26
A_domestic_duck_Poland_H473-T_2022_H5N1_2022-12-22
A_domestic_duck_Poland_H452-T_2022_H5N1_2022-12-22
A_turkey_Poland_H26-T_2023_H5N1_2023-01-07
A_domestic_duck_Poland_H439_2022_H5N1_2022-12-19
A_domestic_goose_Poland_H458-T_2022_H5N1_2022-12-23
A_laying_hen_Poland_H443_2022_H5N1_2022-12-19
A_domestic_duck_Poland_H470-K2T_2022_H5N1_2022-12-27
A_domestic_duck_Poland_H477-T_2022_H5N1_2022-12-28
A_domestic_goose_Poland_H440_2022_H5N1_2022-12-17
A_mute_swan_Austria_22155612_2022_H5N1_2022-12-28
A_mute_swan_Poland_MB014_2023_H5N1_2023-01-10
A_domestic_goose_Slovakia_140_23VIR1686-6_2023_H5N1_2023-01-21
A_domestic_cat_Poland_H246-M_2023_H5N1_2023-06-21_Gdynia_pomorskie
A_domestic_cat_Poland_H257-G_2023_H5N1_2023-06-24_Lublin
A_domestic_cat_Poland_H277-W1_2023_H5N1_2023-06-26_Namyslow_opolskie
A_domestic_cat_Poland_H247_2023_H5N1_2023-06-20_Gdansk
A_domestic_cat_Poland_H271-W_2023_H5N1_lubelskie
A_domestic_cat_Poland_H264-G_2023_H5N1_2023-06-24_Poznan
A_domestic_cat_Poland_H253_2023_H5N1_2023-06-22_Lublin
A_domestic_cat_Poland_H263-G_2023_H5N1_2023-06-24_Komarow-Osada
A_domestic_cat_Poland_H267-W_2023_H5N1_Strzelin_dolnoslaskie
A_domestic_cat_Poland_H266-W_2023_H5N1_2023-06-19_Bydgoszcz_kujawsko-pomorskie
A_domestic_cat_Poland_H249_2023_H5N1_2023-06-22_Gdansk
A_domestic_cat_Poland_H270-W_2023_H5N1_lubelskie
A_cat_Poland_Gda1_2023_H5N1_2023-06-21
A_domestic_cat_Poland_Kot2_2023_H5N1_2023-06-19_Poznan
A_domestic_cat_Poland_H248_2023_H5N1_2023-06-15_Pruszcz_Gdanski
A_domestic_cat_Poland_H252_2023_H5N1_2023-06-22_Lublin
A_domestic_cat_Poland_H254_2023_H5N1_2023-06-22_Lublin
A_domestic_cat_Poland_Kot1_2023_H5N1_2023-06-19_Poznan
A_domestic_cat_Poland_H256-G_2023_H5N1_2023-06-24_Lublin
A_domestic_cat_Poland_H255-M_2023_H5N1_2023-06-21_Pruszcz_Gd
A_white_stork_Poland_MB244_2023_H5N1_2023-06-04_tarnowski
CH
100
A_black-headed_gull_Poland_MB131-J_2023_H5N1_2023-03-23
A_black-headed_gull_Poland_MB180-NMJ_2023_H5N1_2023-05-09
A_black-headed_gull_Poland_MB168-M2_2023_H5N1_2023-05-05
A_common_tern_Poland_MB182-NJ_2023_H5N1_2023-05-10
A_black-headed_gull_Poland_MB166_2023_H5N1_2023-05-05
A_black-headed_gull_Poland_MB129-NJ_2023_H5N1_2023-03-22
A_black-headed_gull_Poland_MB210_2023_H5N1_2023-05-16
A_Mediterranean_gull_Poland_MB204_2023_H5N1_2023-05-08
A_black-headed_gull_Poland_MB176-M6_2023_H5N1_2023-05-05
A_black-headed_gull_Poland_MB185-NJ_2023_H5N1_2023-05-10
A_white_stork_Poland_MB274-M2_2023_H5N1_2023-07-07_malopolskie
A_white_stork_Poland_MB274-M3_2023_H5N1_2023-07-07_malopolskie
A_black-headed_gull_Poland_MB209-NJ_2023_H5N1_2023
A_mute_swan_Poland_MB203-NJ_2023_H5N1_2023-05-11
A_black-headed_gull_Poland_MB198-NJ_2023_H5N1_2023-05-11
A_black-headed_gull_Poland_MB081_2023_H5N1_2023-02-07
A_black-headed_gull_Poland_MB121-M_2023_H5N1_2023-03-13
A_black-headed_gull_Poland_MB211_2023_H5N1_2023-05-14
A_black-headed_gull_Poland_MB141-T_2023_H5N1_2023-04-20
BB
A_chicken_Czech_Republic_67_orig_2023_H5N1_2022-12-30
A_chicken_Czech_Republic_727_orig_2023_H5N1_2023-01-15
A_chicken_Czech_Republic_574_2023_H5N1_2023-01-11
A_chicken_Czech_Republic_574_orig_2023_H5N1_2023-01-11
A_chicken_Czech_Republic_102_orig_2023_H5N1_2023-01-02
0.07

[a]list of the sequences used for above analyses.

We gratefully acknowledge the authors, originating and submitting laboratories of the sequences from GISAID's EpiFlu™ Database on which this research is based in part. The list is detailed below.

All submitters of data may be contacted directly via www.gisaid.org

Isolate-ID	Country	Collection date	Isolatename	Originating Lab	Submitting Lab	Authors
EPI_ISL_17514173	Austria	2023-Jan-30	A/black-headedgull/Austria/23010977-001/2023	Institute for Veterinary Disease Control Moedling, Austrian Agency for Health and Food Safety	Austrian Agency for Health and Food Safety (AGES)	n.a.
EPI_ISL_17514178	Austria	2023-Mar-20	A/black-headed_gull/Austria/23036780-003/2023	Institute for Veterinary Disease Control Moedling, Austrian Agency for Health and Food Safety	Austrian Agency for Health and Food Safety (AGES)	n.a.
EPI_ISL_17512128	Austria	2022-Dec-28	A/mute_swan/Austria/22155612/2022	Institute for Veterinary Disease Control Moedling, Austrian Agency for Health and Food Safety	Austrian Agency for Health and Food Safety (AGES)	n.a.
EPI_ISL_17716087	Austria	2023-Mar-02	A/mute_swan/Austria/23026929-001/2023	Austrian Agency for Health and Food Safety (AGES)	Austrian Agency for Health and Food Safety (AGES)	n.a.
EPI_ISL_17514169	Austria	2023-Feb-28	A/gray_heron/Austria/23025449/2023	Institute for Veterinary Disease Control Moedling, Austrian Agency for Health and Food Safety	Austrian Agency for Health and Food Safety (AGES)	n.a.
EPI_ISL_17514127	Austria	2023-Jan-04	A/great_egret/Austria/23000725-001/2023	Institute for Veterinary Disease Control Moedling, Austrian Agency for Health and Food Safety	Austrian Agency for Health and Food Safety (AGES)	n.a.
EPI_ISL_17716086	Austria	2023-Feb-28	A/heron/Austria/23025431-001/2023	Austrian Agency for Health and Food Safety (AGES)	Austrian Agency for Health and Food Safety (AGES)	n.a.
EPI_ISL_17511728	Austria	2023-Jan-02	A/duck/Austria/23000036/2023	NRL for avian influenza Moedling	Austrian Agency for Health and Food Safety (AGES)	'Institute_for_Veterinary_Disease_Control_Moedling, Institute_for_Medical_Microbiology_and_Hygiene_Vienna'
EPI_ISL_17514127	Austria	2023-Jan-04	A/great_egret/Austria/23000725-001/2023	Institute for Veterinary Disease Control Moedling, Austrian Agency for Health and Food Safety	Austrian Agency for Health and Food Safety (AGES)	n.a.
EPI_ISL_17514173	Austria	2023-Jan-30	A/black-headed gull/Austria/23010977-001/2023	Institute for Veterinary Disease Control Moedling, Austrian Agency for Health and Food Safety	Austrian Agency for Health and Food Safety (AGES)	n.a.
EPI_ISL_17246921	Belgium	2023-Mar-02	A/Mustela_putorius/Belgium/01922_0001/2023	Sciensano - Animal Infectious Diseases	Sciensano, Department of Animal Infectious Diseases	Van Borm, Steven; Roupie, Virginie; Hostyn, Pierre; Mathijs, Elisabeth; Lambrecht, Benedicte; Steensels, Mieke
EPI_ISL_17245142	Belgium	2023-Feb-23	A/Gallus_gallus/Belgium/01688_0004/2023	Sciensano - Animal Infectious Diseases	Sciensano, Department of Animal Infectious Diseases	Van Borm, Steven; Roupie, Virginie; Hostyn, Pierre; Mathijs, Elisabeth; Lambrecht, Benedicte; Steensels, Mieke
EPI_ISL_17015365	Czech Republic	2023-Feb-17	A/domestic_goose/Czech_Republic/2753/2023	State Veterinary Institute Prague	State Veterinary Institute Prague	Alexander,Nagy;Lenka,Cernikova;Martina,Stara

EPI_ISL_16997759	Czech Republic	2023-Feb-06	A/mute_swan/Czech_Republic/2022/2023	State Veterinary Institute Prague	State Veterinary Institute Prague	Alexander,Nagy;Lenka,Cernikova;Martina,Stara
EPI_ISL_16937306	Czech Republic	2023-Jan-16	A/swan/Czech_Republic/811-2/2023	State Veterinary Institute Prague	State Veterinary Institute Prague	Alexander,Nagy;Lenka,Cernikova;Martina,Stara
EPI_ISL_17584808	Czech Republic	2023-Mar-27	A/chicken/Czech_Reublic/4761-2/2023	State Veterinary Institute Prague	State Veterinary Institute Prague	Alexander,Nagy;Lenka,Cernikova;Martina,Stara
EPI_ISL_17164988	Czech Republic	2022-Dec-15	A/chicken/Czech_Republic/23309-4orig/2022	State Veterinary Institute Prague	State Veterinary Institute Prague	Alexander,Nagy;Lenka,Cernikova;Martina,Stara
EPI_ISL_17164987	Czech Republic	2022-Dec-15	A/chicken/Czech_Republic/23309-2orig/2022	State Veterinary Institute Prague	State Veterinary Institute Prague	Alexander,Nagy;Lenka,Cernikova;Martina,Stara
EPI_ISL_17164986	Czech Republic	2022-Dec-15	A/chicken/Czech_Republic/23309-1orig/2022	State Veterinary Institute Prague	State Veterinary Institute Prague	Alexander,Nagy;Lenka,Cernikova;Martina,Stara
EPI_ISL_16997753	Czech Republic	2023-Jan-30	A/chicken/Czech_Republic/1690_orig/2023	State Veterinary Institute Prague	State Veterinary Institute Prague	Alexander,Nagy;Lenka,Cernikova;Martina,Stara
EPI_ISL_16997752	Czech Republic	2023-Jan-30	A/chicken/Czech_Republic/1690/2023	State Veterinary Institute Prague	State Veterinary Institute Prague	Alexander,Nagy;Lenka,Cernikova;Martina,Stara
EPI_ISL_16939160	Czech Republic	2023-Jan-13	A/chicken/Czech_Republic/872_orig/2023	State Veterinary Institute Prague	State Veterinary Institute Prague	Alexander,Nagy;Lenka,Cern?kova;Martina,Stara
EPI_ISL_16937305	Czech Republic	2023-Jan-15	A/chicken/Czech_Republic/727_orig/2023	State Veterinary Institute Prague	State Veterinary Institute Prague	Alexander,Nagy;Lenka,Cernikova;Martina,Stara
EPI_ISL_16937297	Czech Republic	2022-Dec-30	A/chicken/Czech_Republic/24009-2/2022	State Veterinary Institute Prague	State Veterinary Institute Prague	Alexander,Nagy;Lenka,Cernikova;Martina,Stara
EPI_ISL_16937296	Czech Republic	2022-Dec-30	A/chicken/Czech_Republic/24009-1/2022	State Veterinary Institute Prague	State Veterinary Institute Prague	Alexander,Nagy;Lenka,Cernikova;Martina,Stara
EPI_ISL_16937294	Czech Republic	2023-Jan-11	A/chicken/Czech_Republic/574_orig/2023	State Veterinary Institute Prague	State Veterinary Institute Prague	Alexander,Nagy;Lenka,Cernikova;Martina,Stara
EPI_ISL_16937293	Czech Republic	2023-Jan-04	A/chicken/Czech_Republic/180/2023	State Veterinary Institute Prague	State Veterinary Institute Prague	Alexander,Nagy;Lenka,Cernikova;Martina,Stara
EPI_ISL_16937292	Czech Republic	2023-Jan-04	A/chicken/Czech_Republic/179/2023	State Veterinary Institute Prague	State Veterinary Institute Prague	Alexander,Nagy;Lenka,Cernikova;Martina,Stara
EPI_ISL_16638696	Czech Republic	2023-Jan-11	A/chicken/Czech_Republic/574/2023	State Veterinary Institute Prague	State Veterinary Institute Prague	Alexander,Nagy;Lenka,Cernikova;Martina,Stara
EPI_ISL_16613712	Czech Republic	2023-Jan-02	A/chicken/Czech_Republic/102_orig/2023	State Veterinary Institute Prague	State Veterinary Institute Prague	Alexander,Nagy;Lenka,Cernikova;Martina,Stara
EPI_ISL_16613711	Czech Republic	2022-Dec-30	A/chicken/Czech_Republic/67_orig/2023	State Veterinary Institute Prague	State Veterinary Institute Prague	Alexander,Nagy;Lenka,Cernikova;Martina,Stara
EPI_ISL_16937295	Czech Republic	2023-Jan-12	A/domestic_duck/Czech_Republic/636_orig/2023	State Veterinary Institute Prague	State Veterinary Institute Prague	Alexander,Nagy;Lenka,Cernikova;Martina,Stara
EPI_ISL_17164994	Czech Republic	2023-Feb-21	A/great_egret/Czech_Republic/3051-2orig/2023	State Veterinary Institute Prague	State Veterinary Institute Prague	Alexander,Nagy;Lenka,Cernikova;Martina,Stara
EPI_ISL_17164993	Czech Republic	2023-Feb-21	A/great_egret/Czech_Republic/3051-1orig/2023	State Veterinary Institute Prague	State Veterinary Institute Prague	Alexander,Nagy;Lenka,Cernikova;Martina,Stara
EPI_ISL_16997758	Czech Republic	2023-Feb-02	A/mallard/Czech_Republic/1792_orig/2023	State Veterinary Institute Prague	State Veterinary Institute Prague	Alexander,Nagy;Lenka,Cernikova;Martina,Stara
EPI_ISL_16937308	Czech Republic	2023-Jan-16	A/mallard/Czech_Republic/1021/2023	State Veterinary Institute Prague	State Veterinary Institute Prague	Alexander,Nagy;Lenka,Cernikova;Martina,Stara
EPI_ISL_16613717	Czech Republic	2023-Jan-05	A/mallard/Czech_Republic/186_orig/2023	State Veterinary Institute Prague	State Veterinary Institute Prague	Alexander,Nagy;Lenka,Cernikova;Martina,Stara

EPI_ISL_16613713	Czech Republic	2023-Jan-04	A/chicken/Czech_Republic/178_shed-1A_orig/2023	State Veterinary Institute Prague	State Veterinary Institute Prague	Alexander,Nagy;Lenka,Cernikova;Martina,Stara
EPI_ISL_16613714	Czech Republic	2023-Jan-04	A/chicken/Czech_Republic/179_shed-1B_orig/2023	State Veterinary Institute Prague	State Veterinary Institute Prague	Alexander,Nagy;Lenka,Cernikova;Martina,Stara
EPI_ISL_16937293	Czech Republic	2023-Jan-04	A/chicken/Czech_Republic/180/2023	State Veterinary Institute Prague	State Veterinary Institute Prague	Alexander,Nagy;Lenka,Cernikova;Martina,Stara
EPI_ISL_17164983	Czech Republic	2023-Jan-04	A/chicken/Czech_Republic/181_shed-3B_orig/2023	State Veterinary Institute Prague	State Veterinary Institute Prague	Alexander,Nagy;Lenka,Cernikova;Martina,Stara
EPI_ISL_17164991	Czech Republic	2023-Jan-02	A/chicken/Czech_Republic/58_shed-2A_orig/2023	State Veterinary Institute Prague	State Veterinary Institute Prague	Alexander,Nagy;Lenka,Cernikova;Martina,Stara
EPI_ISL_16937308	Czech Republic	2023-Jan-16	A/mallard/Czech_Republic/1021/2023	State Veterinary Institute Prague	State Veterinary Institute Prague	Alexander,Nagy;Lenka,Cernikova;Martina,Stara
EPI_ISL_16997759	Czech Republic	2023-Feb-06	A/mute_swan/Czech_Republic/2022/2023	State Veterinary Institute Prague	State Veterinary Institute Prague	Alexander,Nagy;Lenka,Cernikova;Martina,Stara
EPI_ISL_17767223	Denmark	2016-Nov-07	A/Tuftedduck/Denmark/11740-LWPL/2016	Southeast Poultry Research Laboratory (USDA-ARS)	Import from public-domain	Leyson,C., Youk,S.S., Smith,D., Dimitrov,K., Lee,D.H., Larsen,L.E., Swayne,D.E.; Pantin-Jackwood,M.J.
EPI_ISL_399767	Denmark	2016-Nov-07	A/tuftedduck/Denmark/11740-LWPL/2016	n.a.	Import from public-domain	Leyson,C.; Youk,S.S.; Smith,D.; Dimitrov,K.; Lee,D.H.; Larsen,L.E.; Swayne,D.E.; Pantin-Jackwood,M.J.; Lee,D.-h.
EPI_ISL_399767	Denmark	2016-Nov-07	A/tufted duck/Denmark/11740-LWPL/2016	n.a.	Import from public-domain	Leyson,C.; Youk,S.S.; Smith,D.; Dimitrov,K.; Lee,D.H.; Larsen,L.E.; Swayne,D.E.; Pantin-Jackwood,M.J.; Lee,D.-h.
EPI_ISL_344539	Egypt	2018-Mar-28	A/Turkey/Egypt/AR550/2018	Poultry Diseases Department ,Faculty of Veterinary Medicine, Beni-Suef University	Friedrich-Loeffler-Institut	Hassan ,KE; King,J ; El-Kady, MF; Abohamra ,S; Pohlmann, A ; Harder, TC
EPI_ISL_389751	Egypt	2017-May-10	A/chicken/Egypt/M14081D/2017	n.a.	Import from public-domain	Kandeil,A.; Webby,R.; McKenzie,P.; Kayali,G.; Ali,M.A.
EPI_ISL_271714	Hungary	2016-Nov-24	A/Mulard_duck/Hungary/59163/2016	National Food Chain Safety Office Veterinary Diagnostic Directorate Laboratory for Molecular Biology	Danam.Vet.Molbiol	Adam, Dan
EPI_ISL_378256	Hungary	2016-Nov-24	A/MulardDuck/Hungary/59163/2016	n.a.	Import from public-domain	Tatar-Kis,T.; Dan,A.; Felfoldi,B.; Balint,A.; Ronai,Z.; Dauphin,G.; Penzes,Z.; El-Attrache,J.; Gardin,Y.; Palya,V.
EPI_ISL_298640	Israel	2016-Dec-20	A/turkey/Israel/1045/2016	n.a.	Import from public-domain	Shkoda,I.; Lapin,K.; Simanov,L.; Lublin,A.
EPI_ISL_16979816	Italy	2023-Jan-04	A/buzzard/Italy/23VIR440-2/2023	Istituto Zooprofilattico Sperimentale delle Venezie, EU/OIE/Reference Laboratory and FAO Reference Centre for AI and ND	Istituto Zooprofilattico Sperimentale Delle Venezie	n.a.
EPI_ISL_18010932	Netherlands	2023-Jun-24	A/SandwichTern/Netherlands/9/2023	Erasmus Medical Center	Erasmus Medical Center	n.a.
EPI_ISL_17821075	Netherlands	2023-Jun-02	A/CommonTern/Netherlands/9/2023	Erasmus Medical Center	Erasmus Medical Center	n.a.
EPI_ISL_17626174	Netherlands	2023-Apr-04	A/Peregrinefalcon/Netherlands/9/2023	Erasmus Medical Center	Erasmus Medical Center	n.a.
EPI_ISL_17267157	Netherlands	2023-Mar-03	A/Europeanherringgull/Netherlands/9/2023	Erasmus Medical Center	Erasmus Medical Center	n.a.

EPI_ISL_17208165	Netherlands	2023-Feb-19	A/Black-headedgull/Netherlands/9/2023	Erasmus Medical Center	Erasmus Medical Center	n.a.
EPI_ISL_16056569	Netherlands	2022-Nov-15	A/Gadwall/Netherlands/3/2022	Erasmus Medical Center	Erasmus Medical Center	n.a.
EPI_ISL_15925882	Netherlands	2022-Oct-21	A/EurasianWigeon/Netherlands/3/2022	Erasmus Medical Center	Erasmus Medical Center	n.a.
EPI_ISL_15759949	Netherlands	2022-Oct-22	A/CommonTeal/Netherlands/3/2022	Erasmus Medical Center	Erasmus Medical Center	n.a.
EPI_ISL_15267024	Netherlands	2022-Jul-10	A/NorthernGannet/Netherlands/3/2022	Erasmus Medical Center	Erasmus Medical Center	n.a.
EPI_ISL_15088306	Netherlands	2022-Aug-18	A/Mallard/Netherlands/3/2022	Erasmus Medical Center	Erasmus Medical Center	n.a.
EPI_ISL_15088303	Netherlands	2022-Aug-29	A/LesserBlack-backedGull/Netherlands/3/2022	Erasmus Medical Center	Erasmus Medical Center	n.a.
EPI_ISL_14233919	Netherlands	2022-Jul-24	A/EurasianSpoonbill/Netherlands/3/2022	Erasmus Medical Center	Erasmus Medical Center	n.a.
EPI_ISL_13613230	Netherlands	2022-Jun-13	A/CommonTern/Netherlands/3/2022	Erasmus Medical Center	Erasmus Medical Center	n.a.
EPI_ISL_13429297	Netherlands	2022-Jun-03	A/SandwichTern/Netherlands/3/2022	Erasmus Medical Center	Erasmus Medical Center	n.a.
EPI_ISL_12514681	Netherlands	2022-Apr-14	A/EuropeanHerringGull/Netherlands/3/2022	Erasmus Medical Center	Erasmus Medical Center	n.a.
EPI_ISL_12514483	Netherlands	2022-Apr-14	A/CaspianGull/Netherlands/3/2022	Erasmus Medical Center	Erasmus Medical Center	n.a.
EPI_ISL_12514425	Netherlands	2022-Apr-14	A/Black-headedgull/Netherlands/3/2022	Erasmus Medical Center	Erasmus Medical Center	n.a.
EPI_ISL_11560325	Netherlands	2022-Mar-15	A/Greatblack-backedGull/Netherlands/3/2022	Erasmus Medical Center	Erasmus Medical Center	n.a.
EPI_ISL_9261745	Netherlands	2022-Jan-11	A/Barnaclegoose/Netherlands/3/2022	Erasmus Medical Center	Erasmus Medical Center	n.a.
EPI_ISL_17267157	Netherlands	2023-Mar-03	A/European_herring_gull/Netherlands/9/2023	Erasmus Medical Center	Erasmus Medical Center	n.a.
EPI_ISL_15925882	Netherlands	2022-Oct-21	A/Eurasian Wigeon/Netherlands/3/2022	Erasmus Medical Center	Erasmus Medical Center	n.a.
EPI_ISL_18032447	Poland	2022-Dec-17	A/domestic_goose/Poland/H440/2022	National Veterinary Research Institut Poland, PIWet-PIB	National Veterinary Research Institut Poland, PIWet-PIB	Swieton,Edyta;Tarasiuk,Karolina;Wyrostek,Krzysztof;Kozak,Edyta;Stys-Fijol,Natalia;Smietanka,Krzysztof
EPI_ISL_18032445	Poland	2023-Feb-14	A/mute_swan/Poland/MB095-L1M/2023	National Veterinary Research Institut Poland, PIWet-PIB	National Veterinary Research Institut Poland, PIWet-PIB	Swieton,Edyta;Tarasiuk,Karolina;Wyrostek,Krzysztof;Kozak,Edyta;Stys-Fijol,Natalia;Smietanka,Krzysztof
EPI_ISL_18032171	Poland	2023-Jan-10	A/mute_swan/Poland/MB015/2023	National Veterinary Research Institut Poland, PIWet-PIB	National Veterinary Research Institut Poland, PIWet-PIB	Swieton, Edyta

EPI_ISL_18032166	Poland	2023-Jan-10	A/mute_swan/Poland/MB014/2023	National Veterinary Research Institut Poland, PIWet-PIB	National Veterinary Research Institut Poland, PIWet-PIB	Swieton, Edyta
EPI_ISL_18033134	Poland	2022-Dec-14	A/turkey/Poland/H427/2022	National Veterinary Research Institut Poland, PIWet-PIB	National Veterinary Research Institut Poland, PIWet-PIB	Swieton,Edyta;Tarasiuk,Karolina;Wyrostek,Krzysztof;Kozak,Edyta;Stys-Fijol,Natalia;Smietanka,Krzysztof
EPI_ISL_17978700	Poland	2023-Jun-04	A/white_stork/Poland/MB244/2023	National Veterinary Research Institut Poland, PIWet-PIB	National Veterinary Research Institut Poland, PIWet-PIB	Swieton, E.; Domanska-Blicharz, K.; Tarasiuk, K.; Wyrostek, K.; Stys-Fijol, N.
EPI_ISL_18040893	Poland	2023-Jan-20	A/domestic_duck/Poland/H89-T1/2023	National Veterinary Research Institut Poland, PIWet-PIB	National Veterinary Research Institut Poland, PIWet-PIB	Swieton,Edyta;Tarasiuk,Karolina;Wyrostek,Krzysztof;Kozak,Edyta;Stys-Fijol,Natalia;Smietanka,Krzysztof
EPI_ISL_18040892	Poland	2023-Jan-19	A/domestic_duck/Poland/H93-T1/2023	National Veterinary Research Institut Poland, PIWet-PIB	National Veterinary Research Institut Poland, PIWet-PIB	Swieton,Edyta;Tarasiuk,Karolina;Wyrostek,Krzysztof;Kozak,Edyta;Stys-Fijol,Natalia;Smietanka,Krzysztof
EPI_ISL_18033137	Poland	2022-Dec-16	A/domestic_duck/Poland/H433/2022	National Veterinary Research Institut Poland, PIWet-PIB	National Veterinary Research Institut Poland, PIWet-PIB	Swieton,Edyta;Tarasiuk,Karolina;Wyrostek,Krzysztof;Kozak,Edyta;Stys-Fijol,Natalia;Smietanka,Krzysztof
EPI_ISL_18032446	Poland	2022-Dec-15	A/domestic_duck/Poland/H429/2022	National Veterinary Research Institut Poland, PIWet-PIB	National Veterinary Research Institut Poland, PIWet-PIB	Swieton,Edyta;Tarasiuk,Karolina;Wyrostek,Krzysztof;Kozak,Edyta;Stys-Fijol,Natalia;Smietanka,Krzysztof
EPI_ISL_18032444	Poland	2023-Jan-16	A/domestic_duck/Poland/H75-K1T1/2023	National Veterinary Research Institut Poland, PIWet-PIB	National Veterinary Research Institut Poland, PIWet-PIB	Swieton,Edyta;Tarasiuk,Karolina;Wyrostek,Krzysztof;Kozak,Edyta;Stys-Fijol,Natalia;Smietanka,Krzysztof
EPI_ISL_18032204	Poland	2023-Jan-16	A/domestic_duck/Poland/H72-T1/2023	National Veterinary Research Institut Poland, PIWet-PIB	National Veterinary Research Institut Poland, PIWet-PIB	Swieton, Edyta
EPI_ISL_17985196	Poland	2023-Jun-19	A/domestic_cat/Poland/Kot2/2023	National Veterinary Research Institut Poland, PIWet-PIB	National Veterinary Research Institut Poland, PIWet-PIB	Edyta,Świętoń; Katarzyna,Domańska-Blicharz; Aleksandra,Giza; Marta,Pietruk; Karolina,Tarasiuk; Krzysztof,Wyrostek; Natalia, Styś-Fijoł
EPI_ISL_17951056	Poland	2023-Jun-19	A/domestic_cat/Poland/Kot1/2023	National Veterinary Research Institut Poland, PIWet-PIB	National Veterinary Research Institut Poland, PIWet-PIB	Edyta,Świętoń; Katarzyna,Domańska-Blicharz; Aleksandra,Giza; Marta,Pietruk; Karolina,Tarasiuk; Krzysztof,Wyrostek; Natalia, Styś-Fijoł
EPI_ISL_17951055	Poland	2023-Jun-20	A/domestic_cat/Poland/H247/2023	National Veterinary Research Institut Poland, PIWet-PIB	National Veterinary Research Institut Poland, PIWet-PIB	Edyta,Świętoń; Katarzyna,Domańska-Blicharz; Aleksandra,Giza; Marta,Pietruk; Karolina,Tarasiuk; Krzysztof,Wyrostek; Natalia, Styś-Fijoł
EPI_ISL_17951054	Poland	2023-Jun-15	A/domestic_cat/Poland/H248/2023	National Veterinary Research Institut Poland, PIWet-PIB	National Veterinary Research Institut Poland, PIWet-PIB	Edyta,Świętoń; Katarzyna,Domańska-Blicharz; Aleksandra,Giza; Marta,Pietruk; Karolina,Tarasiuk; Krzysztof,Wyrostek; Natalia, Styś-Fijoł
EPI_ISL_17951053	Poland	2023-Jun-22	A/domestic_cat/Poland/H254/2023	National Veterinary Research Institut Poland, PIWet-PIB	National Veterinary Research Institut Poland, PIWet-PIB	Edyta,Świętoń; Katarzyna,Domańska-Blicharz; Aleksandra,Giza; Marta,Pietruk; Karolina,Tarasiuk; Krzysztof,Wyrostek; Natalia, Styś-Fijoł
EPI_ISL_17951052	Poland	2023-Jun-22	A/domestic_cat/Poland/H253/2023	National Veterinary Research Institut Poland, PIWet-PIB	National Veterinary Research Institut Poland, PIWet-PIB	Edyta,Świętoń; Katarzyna,Domańska-Blicharz; Aleksandra,Giza; Marta,Pietruk; Karolina,Tarasiuk; Krzysztof,Wyrostek; Natalia, Styś-Fijoł
EPI_ISL_17951051	Poland	2023-Jun-22	A/domestic_cat/Poland/H252/2023	National Veterinary Research Institut Poland, PIWet-PIB	National Veterinary Research Institut Poland, PIWet-PIB	Edyta,Świętoń; Katarzyna,Domańska-Blicharz; Aleksandra,Giza; Marta,Pietruk; Karolina,Tarasiuk; Krzysztof,Wyrostek; Natalia, Styś-Fijoł

EPI_ISL_17950995	Poland	2023-Jun-22	A/domestic_cat/Poland/H249/2023	National Veterinary Research Institut Poland, PIWet-PIB	National Veterinary Research Institut Poland, PIWet-PIB	Edyta,Świętoń; Katarzyna,Domańska-Blicharz; Aleksandra,Giza; Marta,Pietruk; Karolina,Tarasiuk; Krzysztof,Wyrostek; Natalia, Styś-Fijoł
EPI_ISL_11922811	Poland	2022-Feb-05	A/goose/Poland/H124_22VIR2515-5/2022	National Veterinary Research Institute	Istituto Zooprofilattico Sperimentale delle Venezie	Swieton, E.; Smietanka, K.; Barbierato, G.; Zecchin, B.; Fusaro, A.; Schivo, A.; Salviato, A.; Palumbo, E.; Giussani, E.; Monne, I.; Terregino, C.
EPI_ISL_11922814	Poland	2022-Feb-17	A/swan/Poland/MB083_22VIR2515-8/2022	National Veterinary Research Institute	Istituto Zooprofilattico Sperimentale delle Venezie	Swieton, E.; Smietanka, K.; Barbierato, G.; Zecchin, B.; Fusaro, A.; Schivo, A.; Salviato, A.; Palumbo, E.; Giussani, E.; Monne, I.; Terregino, C.
EPI_ISL_11922813	Poland	2022-Feb-10	A/swan/Poland/MB078_22VIR2515-7/2022	National Veterinary Research Institute	Istituto Zooprofilattico Sperimentale delle Venezie	Swieton, E.; Smietanka, K.; Barbierato, G.; Zecchin, B.; Fusaro, A.; Schivo, A.; Salviato, A.; Palumbo, E.; Giussani, E.; Monne, I.; Terregino, C.
EPI_ISL_6937114	Poland	2021-Nov-08	A/mute_swan/Poland/MB490-L1/2021	National Veterinary Research Institut Poland, PIWet-PIB	National Veterinary Research Institut Poland, PIWet-PIB	E. Swieton, K. Smietanka
EPI_ISL_17959737	Poland	2023-Jun-09	A/environment/Poland/Kra1/2023	Malopolska Centre of Biotechnology of the Jagiellonian University	University of Gdansk	Maciej, Grzybek; Tomasz, Lepionka; Krzysztof, Pyrc; Lukasz, Rabalski
EPI_ISL_2681045	Poland	2021-Apr-20	A/white_stork/Poland/MB391/2021	National Veterinary Research Institut Poland, PIWet-PIB	National Veterinary Research Institut Poland, PIWet-PIB	Edyta, Swieton; Kamila, Dziadek; Krzysztof, Smietanka
EPI_ISL_11922812	Poland	2022-Jan-23	A/chicken/Poland/H071_22VIR2515-6/2022	National Veterinary Research Institute	Istituto Zooprofilattico Sperimentale delle Venezie	Swieton, E.; Smietanka, K.; Barbierato, G.; Zecchin, B.; Fusaro, A.; Schivo, A.; Salviato, A.; Palumbo, E.; Giussani, E.; Monne, I.; Terregino, C.
EPI_ISL_11922809	Poland	2022-Feb-18	A/chicken/Poland/H157_22VIR2515-3/2022	National Veterinary Research Institute	Istituto Zooprofilattico Sperimentale delle Venezie	Swieton, E.; Smietanka, K.; Barbierato, G.; Zecchin, B.; Fusaro, A.; Schivo, A.; Salviato, A.; Palumbo, E.; Giussani, E.; Monne, I.; Terregino, C.
EPI_ISL_11922810	Poland	2022-Feb-08	A/duck/Poland/H126_22VIR2515-4/2022	National Veterinary Research Institute	Istituto Zooprofilattico Sperimentale delle Venezie	Swieton, E.; Smietanka, K.; Barbierato, G.; Zecchin, B.; Fusaro, A.; Schivo, A.; Salviato, A.; Palumbo, E.; Giussani, E.; Monne, I.; Terregino, C.
EPI_ISL_11922808	Poland	2022-Mar-02	A/duck/Poland/H188_22VIR2515-2/2022	National Veterinary Research Institute	Istituto Zooprofilattico Sperimentale delle Venezie	Swieton, E.; Smietanka, K.; Barbierato, G.; Zecchin, B.; Fusaro, A.; Schivo, A.; Salviato, A.; Palumbo, E.; Giussani, E.; Monne, I.; Terregino, C.
EPI_ISL_6934175	Poland	2021-Nov-07	A/domestic_duck/Poland/H1942-N/2021	National Veterinary Research Institut Poland, PIWet-PIB	National Veterinary Research Institut Poland, PIWet-PIB	E. Swieton, K. Smietanka
EPI_ISL_14917968	Poland	2022-Jul-13	A/common_murre/Poland/MB151/2022	National Veterinary Research Institut Poland, PIWet-PIB	National Veterinary Research Institut Poland, PIWet-PIB	Swieton E., Smietanka K.
EPI_ISL_14917942	Poland	2022-Jul-21	A/breeding_hen/Poland/H364/2022	National Veterinary Research Institut Poland, PIWet-PIB	National Veterinary Research Institut Poland, PIWet-PIB	Swieton E., Smietanka K.
EPI_ISL_14917915	Poland	2022-Jul-18	A/breeding_hen/Poland/H361/2022	National Veterinary Research Institut Poland, PIWet-PIB	National Veterinary Research Institut Poland, PIWet-PIB	Swieton E., Smietanka K.
EPI_ISL_6931288	Poland	2021-Nov-05	A/chicken/Poland/H1940-N/2021	National Veterinary Research Institut Poland, PIWet-PIB	National Veterinary Research Institut Poland, PIWet-PIB	E. Swieton, K. Smietanka

EPI_ISL_6931008	Poland	2021-Nov-03	A/domestic_goose/Poland/H1931-T1/2021	National Veterinary Research Institut Poland, PIWet-PIB	National Veterinary Research Institut Poland, PIWet-PIB	E. Swieton, K. Smietanka
EPI_ISL_14917999	Poland	2022-May-30	A/black-headed_gull/Poland/MB139/2022	National Veterinary Research Institut Poland, PIWet-PIB	National Veterinary Research Institut Poland, PIWet-PIB	Swieton E., Smietanka K.
EPI_ISL_15535272	Poland	2022-Sep-20	A/domestic_goose/Poland/H397-N/2022	National Veterinary Research Institut Poland, PIWet-PIB	National Veterinary Research Institut Poland, PIWet-PIB	Świętoń E., Śmietanka K.
EPI_ISL_14917951	Poland	2022-Jul-21	A/domestic_goose/Poland/H371/2022	National Veterinary Research Institut Poland, PIWet-PIB	National Veterinary Research Institut Poland, PIWet-PIB	Swieton E., Smietanka K.
EPI_ISL_14917979	Poland	2022-May-30	A/herring_gull/Poland/MB138/2022	National Veterinary Research Institut Poland, PIWet-PIB	National Veterinary Research Institut Poland, PIWet-PIB	Swieton E., Smietanka K.
EPI_ISL_6935584	Poland	2021-Nov-08	A/turkey/Poland/H1944-N/2021	National Veterinary Research Institut Poland, PIWet-PIB	National Veterinary Research Institut Poland, PIWet-PIB	E. Swieton, K. Smietanka
EPI_ISL_6930564	Poland	2021-Nov-03	A/turkey/Poland/H1924-T1/2021	National Veterinary Research Institut Poland, PIWet-PIB	National Veterinary Research Institut Poland, PIWet-PIB	E. Swieton, K. Smietanka
EPI_ISL_6930238	Poland	2021-Nov-02	A/turkey/Poland/H1913-T1/2021	National Veterinary Research Institut Poland, PIWet-PIB	National Veterinary Research Institut Poland, PIWet-PIB	E. Swieton, K. Smietanka
EPI_ISL_6929970	Poland	2021-Nov-01	A/turkey/Poland/H1911-N/2021	National Veterinary Research Institut Poland, PIWet-PIB	National Veterinary Research Institut Poland, PIWet-PIB	E. Swieton, K. Smietanka
EPI_ISL_6929958	Poland	2021-Nov-01	A/turkey/Poland/H1910-T3/2021	National Veterinary Research Institut Poland, PIWet-PIB	National Veterinary Research Institut Poland, PIWet-PIB	E. Swieton, K. Smietanka
EPI_ISL_17949824	Poland	2023-Jun-21	A/cat/Poland/Gda1/2023	University of Gdansk	University of Gdansk	Maciej,Grzybek; Tomasz,Lepionka; Krzysztof,Pyrc; Lukasz,Rabalski
EPI_ISL_17989196	Poland	2023-Jun-22	A/cat/Poland/Kra1/2023	Malopolska Centre of Biotechnology of the Jagiellonian University	University of Gdansk	Maciej, Grzybek; Tomasz, Lepionka; Krzysztof, Pyrc; Lukasz, Rabalski
EPI_ISL_17971998	Poland	2023-Jun-26	A/domestic_cat/Poland/H264-G/2023	National Veterinary Research Institut Poland, PIWet-PIB	National Veterinary Research Institut Poland, PIWet-PIB	Swieton, E.;Domanska-Blicharz, K.;Tarasiuk, K.; Wyrostek, K.;Stys-Fijol, N.
EPI_ISL_17971997	Poland	2023-Jun-26	A/domestic_cat/Poland/H277-W1/2023	National Veterinary Research Institut Poland, PIWet-PIB	National Veterinary Research Institut Poland, PIWet-PIB	Swieton, E.;Domanska-Blicharz, K.;Tarasiuk, K.; Wyrostek, K.;Stys-Fijol, N.
EPI_ISL_17971996	Poland	2023-Jun-25	A/domestic_cat/Poland/H271-W/2023	National Veterinary Research Institut Poland, PIWet-PIB	National Veterinary Research Institut Poland, PIWet-PIB	Swieton, E.;Domanska-Blicharz, K.;Tarasiuk, K.; Wyrostek, K.;Stys-Fijol, N.
EPI_ISL_17971995	Poland	2023-Jun-25	A/domestic_cat/Poland/H270-W/2023	National Veterinary Research Institut Poland, PIWet-PIB	National Veterinary Research Institut Poland, PIWet-PIB	Swieton, E.;Domanska-Blicharz, K.;Tarasiuk, K.; Wyrostek, K.;Stys-Fijol, N.

EPI_ISL_17971994	Poland	2023-Jun-25	A/domestic_cat/Poland/H267-W/2023	National Veterinary Research Institut Poland, PIWet-PIB	National Veterinary Research Institut Poland, PIWet-PIB	Swieton, E.;Domanska-Blicharz, K.;Tarasiuk, K.; Wyrostek, K.;Stys-Fijol, N.
EPI_ISL_17971993	Poland	2023-Jun-19	A/domestic_cat/Poland/H266-W/2023	National Veterinary Research Institut Poland, PIWet-PIB	National Veterinary Research Institut Poland, PIWet-PIB	Swieton, E.;Domanska-Blicharz, K.;Tarasiuk, K.; Wyrostek, K.;Stys-Fijol, N.
EPI_ISL_17971992	Poland	2023-Jun-26	A/domestic_cat/Poland/H263-G/2023	National Veterinary Research Institut Poland, PIWet-PIB	National Veterinary Research Institut Poland, PIWet-PIB	Swieton, E.;Domanska-Blicharz, K.;Tarasiuk, K.; Wyrostek, K.;Stys-Fijol, N.
EPI_ISL_17971991	Poland	2023-Jun-24	A/domestic_cat/Poland/H257-G/2023	National Veterinary Research Institut Poland, PIWet-PIB	National Veterinary Research Institut Poland, PIWet-PIB	Swieton, E.;Domanska-Blicharz, K.;Tarasiuk, K.; Wyrostek, K.;Stys-Fijol, N.
EPI_ISL_17971990	Poland	2023-Jun-24	A/domestic_cat/Poland/H256-G/2023	National Veterinary Research Institut Poland, PIWet-PIB	National Veterinary Research Institut Poland, PIWet-PIB	Swieton, E.;Domanska-Blicharz, K.;Tarasiuk, K.; Wyrostek, K.;Stys-Fijol, N.
EPI_ISL_17971989	Poland	2023-Jun-21	A/domestic_cat/Poland/H255-M/2023	National Veterinary Research Institut Poland, PIWet-PIB	National Veterinary Research Institut Poland, PIWet-PIB	Swieton, E.;Domanska-Blicharz, K.;Tarasiuk, K.; Wyrostek, K.;Stys-Fijol, N.
EPI_ISL_17956379	Poland	2023-Jun-21	A/domestic_cat/Poland/H255-M/2023	National Veterinary Research Institut Poland, PIWet-PIB	National Veterinary Research Institut Poland, PIWet-PIB	Edyta,Świętoń; Katarzyna,Domańska-Blicharz; Aleksandra,Giza; Marta,Pietruk; Karolina,Tarasiuk; Krzysztof,Wyrostek; Natalia, Styś-Fijoł
EPI_ISL_17956378	Poland	2023-Jun-21	A/domestic_cat/Poland/H246-M/2023	National Veterinary Research Institut Poland, PIWet-PIB	National Veterinary Research Institut Poland, PIWet-PIB	Edyta,Świętoń; Katarzyna,Domańska-Blicharz; Aleksandra,Giza; Marta,Pietruk; Karolina,Tarasiuk; Krzysztof,Wyrostek; Natalia, Styś-Fijoł
EPI_ISL_17165512	Romania	2023-Jan-26	A/turkey/Romania/10271_23VIR1145-13/2023	Institute for Diagnosis & Animal Health (IDAH)	Istituto Zooprofilattico Sperimentale Delle Venezie	Barbuceanu, F.; Onita, I.; Neicut, A.; Motiu, R.; Burlacu, R.; Pastori, A.; Zecchin, B.; Fusaro, A.; Schivo, A.; Salviato, A.; Palumbo, E.; Giussani, E.; Monne, I.; Terregino, C.
EPI_ISL_17165511	Romania	2023-Jan-28	A/turkey/Romania/10287_23VIR1145-12/2023	Institute for Diagnosis & Animal Health (IDAH)	Istituto Zooprofilattico Sperimentale Delle Venezie	Barbuceanu, F.; Onita, I.; Neicut, A.; Motiu, R.; Burlacu, R.; Pastori, A.; Zecchin, B.; Fusaro, A.; Schivo, A.; Salviato, A.; Palumbo, E.; Giussani, E.; Monne, I.; Terregino, C.
EPI_ISL_17165510	Romania	2023-Jan-27	A/turkey/Romania/10281_23VIR1145-11/2023	Institute for Diagnosis & Animal Health (IDAH)	Istituto Zooprofilattico Sperimentale Delle Venezie	Barbuceanu, F.; Onita, I.; Neicut, A.; Motiu, R.; Burlacu, R.; Pastori, A.; Zecchin, B.; Fusaro, A.; Schivo, A.; Salviato, A.; Palumbo, E.; Giussani, E.; Monne, I.; Terregino, C.
EPI_ISL_17165509	Romania	2023-Jan-27	A/turkey/Romania/10281_23VIR1145-10/2023	Institute for Diagnosis & Animal Health (IDAH)	Istituto Zooprofilattico Sperimentale Delle Venezie	Barbuceanu, F.; Onita, I.; Neicut, A.; Motiu, R.; Burlacu, R.; Pastori, A.; Zecchin, B.; Fusaro, A.; Schivo, A.; Salviato, A.; Palumbo, E.; Giussani, E.; Monne, I.; Terregino, C.
EPI_ISL_17165507	Romania	2023-Jan-17	A/cygnus_cygnus/Romania/10164_23VIR1145-8/2023	Institute for Diagnosis & Animal Health (IDAH)	Istituto Zooprofilattico Sperimentale Delle Venezie	Barbuceanu, F.; Onita, I.; Neicut, A.; Motiu, R.; Burlacu, R.; Pastori, A.; Zecchin, B.; Fusaro, A.; Schivo, A.; Salviato, A.; Palumbo, E.; Giussani, E.; Monne, I.; Terregino, C.
EPI_ISL_17165502	Romania	2023-Jan-03	A/cygnus_cygnus/Romania/10008_23VIR1145-3/2023	Institute for Diagnosis & Animal Health (IDAH)	Istituto Zooprofilattico Sperimentale Delle Venezie	Barbuceanu, F.; Onita, I.; Neicut, A.; Motiu, R.; Burlacu, R.; Pastori, A.; Zecchin, B.; Fusaro, A.; Schivo, A.; Salviato, A.; Palumbo, E.; Giussani, E.; Monne, I.; Terregino, C.

EPI_ISL_17165498	Romania	2023-Feb-01	A/corvus_corvus/Romania/10368_23VIR1368-1/2023	Institute for Diagnosis & Animal Health (IDAH)	Istituto Zooprofilattico Sperimentale Delle Venezie	Barbuceanu, F.; Onita, I.; Neicut, A.; Motiu, R.; Burlacu, R.; Pastori, A.; Zecchin, B.; Fusaro, A.; Schivo, A.; Salviato, A.; Palumbo, E.; Giussani, E.; Monne, I.; Terregino, C.
EPI_ISL_17165499	Romania	2023-Feb-02	A/anas_platyrhynchos/Romania/10368_23VIR1368-2/2023	Institute for Diagnosis & Animal Health (IDAH)	Istituto Zooprofilattico Sperimentale Delle Venezie	Barbuceanu, F.; Onita, I.; Neicut, A.; Motiu, R.; Burlacu, R.; Pastori, A.; Zecchin, B.; Fusaro, A.; Schivo, A.; Salviato, A.; Palumbo, E.; Giussani, E.; Monne, I.; Terregino, C.
EPI_ISL_17679820	Slovakia	2023-Jan-21	A/domestic_goose/Slovakia/140_23VIR1686-6/2023	State Veterinary Institute	Istituto Zooprofilattico Sperimentale Delle Venezie	Dirbakova, Z.; Tinak, M.; Pastori, A.; Zecchin, B.; Fusaro, A.; Schivo, A.; Salviato, A.; Palumbo, E.; Giussani, E.; Monne, I.; Terregino, C.
EPI_ISL_17679819	Slovakia	2023-Jan-20	A/mute_swan/Slovakia/139_23VIR1686-5/2023	State Veterinary Institute	Istituto Zooprofilattico Sperimentale Delle Venezie	Dirbakova, Z.; Tinak, M.; Pastori, A.; Zecchin, B.; Fusaro, A.; Schivo, A.; Salviato, A.; Palumbo, E.; Giussani, E.; Monne, I.; Terregino, C.
EPI_ISL_17679817	Slovakia	2023-Jan-12	A/chicken/Slovakia/25-brain_23VIR1686-3/2023	State Veterinary Institute	Istituto Zooprofilattico Sperimentale Delle Venezie	Dirbakova, Z.; Tinak, M.; Pastori, A.; Zecchin, B.; Fusaro, A.; Schivo, A.; Salviato, A.; Palumbo, E.; Giussani, E.; Monne, I.; Terregino, C.
EPI_ISL_17679816	Slovakia	2023-Jan-12	A/chicken/Slovakia/25-organs_23VIR1686-2/2023	State Veterinary Institute	Istituto Zooprofilattico Sperimentale Delle Venezie	Dirbakova, Z.; Tinak, M.; Pastori, A.; Zecchin, B.; Fusaro, A.; Schivo, A.; Salviato, A.; Palumbo, E.; Giussani, E.; Monne, I.; Terregino, C.
EPI_ISL_17679815	Slovakia	2023-Jan-12	A/chicken/Slovakia/25-swab_23VIR1686-1/2023	State Veterinary Institute	Istituto Zooprofilattico Sperimentale Delle Venezie	Dirbakova, Z.; Tinak, M.; Pastori, A.; Zecchin, B.; Fusaro, A.; Schivo, A.; Salviato, A.; Palumbo, E.; Giussani, E.; Monne, I.; Terregino, C.
EPI_ISL_17679794	Slovenia	2023-Feb-02	A/black_swan/Slovenia/137_23VIR1977-5/2023	University of Ljubljana	Istituto Zooprofilattico Sperimentale Delle Venezie	Slavec, B.; Kvapil, P.; Paller, T.; Pastori, A.; Zecchin, B.; Fusaro, A.; Schivo, A.; Salviato, A.; Palumbo, E.; Giussani, E.; Monne, I.; Terregino, C.
EPI_ISL_17679793	Slovenia	2023-Jan-31	A/mute_swan/Slovenia/124_23VIR1977-4/2023	University of Ljubljana	Istituto Zooprofilattico Sperimentale Delle Venezie	Slavec, B.; Kvapil, P.; Paller, T.; Pastori, A.; Zecchin, B.; Fusaro, A.; Schivo, A.; Salviato, A.; Palumbo, E.; Giussani, E.; Monne, I.; Terregino, C.
EPI_ISL_17679795	Slovenia	2023-Feb-02	A/Eurasian_eagle-owl/Slovenia/142_23VIR1977-6/2023	University of Ljubljana	Istituto Zooprofilattico Sperimentale Delle Venezie	Slavec, B.; Kvapil, P.; Paller, T.; Pastori, A.; Zecchin, B.; Fusaro, A.; Schivo, A.; Salviato, A.; Palumbo, E.; Giussani, E.; Monne, I.; Terregino, C.
EPI_ISL_17679792	Slovenia	2023-Jan-04	A/mute_swan/Slovenia/6_23VIR1977-3/2023	University of Ljubljana	Istituto Zooprofilattico Sperimentale Delle Venezie	Slavec, B.; Kvapil, P.; Paller, T.; Pastori, A.; Zecchin, B.; Fusaro, A.; Schivo, A.; Salviato, A.; Palumbo, E.; Giussani, E.; Monne, I.; Terregino, C.
EPI_ISL_17679793	Slovenia	2023-Jan-31	A/mute_swan/Slovenia/124_23VIR1977-4/2023	University of Ljubljana	Istituto Zooprofilattico Sperimentale Delle Venezie	Slavec, B.; Kvapil, P.; Paller, T.; Pastori, A.; Zecchin, B.; Fusaro, A.; Schivo, A.; Salviato, A.; Palumbo, E.; Giussani, E.; Monne, I.; Terregino, C.
EPI_ISL_17679794	Slovenia	2023-Feb-02	A/black_swan/Slovenia/137_23VIR1977-5/2023	University of Ljubljana	Istituto Zooprofilattico Sperimentale Delle Venezie	Slavec, B.; Kvapil, P.; Paller, T.; Pastori, A.; Zecchin, B.; Fusaro, A.; Schivo, A.; Salviato, A.; Palumbo, E.; Giussani, E.; Monne, I.; Terregino, C.
EPI_ISL_17679795	Slovenia	2023-Feb-02	A/Eurasian_eagle-owl/Slovenia/142_23VIR1977-6/2023	University of Ljubljana	Istituto Zooprofilattico Sperimentale Delle Venezie	Slavec, B.; Kvapil, P.; Paller, T.; Pastori, A.; Zecchin, B.; Fusaro, A.; Schivo, A.; Salviato, A.; Palumbo, E.; Giussani, E.; Monne, I.; Terregino, C.
EPI_ISL_18042387	Slovenia	2023-Feb-06	A/grey_heron/Slovenia/163_23VIR1977-7/2023	University of Ljubljana	Istituto Zooprofilattico Sperimentale Delle Venezie	Slavec, B.; Kvapil, P.; Paller, T.; Pastori, A.; Zecchin, B.; Fusaro, A.; Schivo, A.; Salviato, A.; Palumbo, E.; Giussani, E.; Monne, I.; Terregino, C.

EPI_ISL_18042388	Slovenia	2023-Feb-17	A/mute_swan/Slovenia/236_23VIR1977-9/2023	University of Ljubljana	Istituto Zooprofilattico Sperimentale Delle Venezie	Slavec, B.; Kvapil, P.; Paller, T.; Pastori, A.; Zecchin, B.; Fusaro, A.; Schivo, A.; Salviato, A.; Palumbo, E.; Giussani, E.; Monne, I.; Terregino, C.
EPI_ISL_18042389	Slovenia	2023-Feb-20	A/mute_swan/Slovenia/237_23VIR1977-8/2023	University of Ljubljana	Istituto Zooprofilattico Sperimentale Delle Venezie	Slavec, B.; Kvapil, P.; Paller, T.; Pastori, A.; Zecchin, B.; Fusaro, A.; Schivo, A.; Salviato, A.; Palumbo, E.; Giussani, E.; Monne, I.; Terregino, C.
EPI_ISL_18042390	Slovenia	2023-Feb-21	A/mute_swan/Slovenia/241_23VIR1977-13/2023	University of Ljubljana	Istituto Zooprofilattico Sperimentale Delle Venezie	Slavec, B.; Kvapil, P.; Paller, T.; Pastori, A.; Zecchin, B.; Fusaro, A.; Schivo, A.; Salviato, A.; Palumbo, E.; Giussani, E.; Monne, I.; Terregino, C.
EPI_ISL_18042391	Slovenia	2023-Feb-21	A/laying_hen/Slovenia/251_23VIR1977-15/2023	University of Ljubljana	Istituto Zooprofilattico Sperimentale Delle Venezie	Slavec, B.; Kvapil, P.; Paller, T.; Pastori, A.; Zecchin, B.; Fusaro, A.; Schivo, A.; Salviato, A.; Palumbo, E.; Giussani, E.; Monne, I.; Terregino, C.
EPI_ISL_18042392	Slovenia	2023-Feb-23	A/mute_swan/Slovenia/242_23VIR1977-11/2023	University of Ljubljana	Istituto Zooprofilattico Sperimentale Delle Venezie	Slavec, B.; Kvapil, P.; Paller, T.; Pastori, A.; Zecchin, B.; Fusaro, A.; Schivo, A.; Salviato, A.; Palumbo, E.; Giussani, E.; Monne, I.; Terregino, C.
EPI_ISL_17181724	Switzerland	2023-Feb-03	A/black-swan/Switzerland-Zurich/230131/2023	Nationales Referenzlabor für Geflügel- und Kaninchenkrankheiten	Institute of Virology and Immunology (IVI)	n.a.

S5. Nucleotide differences among the sequences of the H5N1 viruses collected from the nineteen cats in Poland (in red)

Nt differences	PB2							PB1						PA										HA							NP	NA			MP				NS		
**Virus**	252	453	837	1328	1416	1707	1945	139	333	336	632	1144	1878	313	501	830	876	1173	1335	1423	1740	1953	2110	32	33	199	552	1023	1455	1704	1170	428	450	1348	98	387	620	851	42	84	106
**A/domestic cat/Poland/H246-M/2023 H5N1 2023-06-21 Gdynia brain**	T	T	G	A	G	G	G	C	**T**	**C**	G	A	T	T	T	C	C	A	T	G	G	A	G	T	T	A	T	G	C	G	C	A	G	A	C	T	G	C	T	T	C
**A/domestic cat/Poland/H255-M/2023 H5N1 2023-06-21 Pruszcz brain**	T	**C**	G	A	G	G	G	C	**T**	**C**	G	A	T	T	T	C	C	A	T	G	G	A	G	T	T	A	T	G	C	G	C	A	G	A	C	T	G	C	T	T	C
**A/domestic cat/Poland/H256-G/2023 H5N1 2023-06-24 Lublin**	**C**	T	**A**	A	G	G	G	C	**T**	A	G	A	T	T	**C**	C	C	A	T	G	G	A	G	T	T	**G**	T	G	C	G	C	A	G	A	**T**	T	G	**T**	T	T	C
**A/domestic cat/Poland/H257-G/2023 H5N1 2023-06-24 Lublin**	T	T	G	A	G	G	G	C	**T**	A	G	A	T	T	T	C	**T**	A	**C**	**A**	G	A	G	T	T	A	T	G	C	G	C	A	G	A	C	T	G	C	T	T	C
**A/domestic cat/Poland/H263-G/2023 H5N1 2023-06-26 Komarow-Osada**	T	T	G	A	G	G	G	C	G	A	G	A	T	T	T	C	C	**G**	T	G	G	A	**A**	T	T	A	T	G	C	G	C	A	G	**G**	C	T	G	C	T	T	C
**A/domestic cat/Poland/H264-G/2023 H5N1 2023-06-26 Poznan**	T	T	G	A	G	G	G	C	G	A	G	A	T	T	T	C	C	A	T	G	G	A	G	T	T	A	T	G	C	G	C	A	G	A	C	T	G	C	T	T	C
**A/domestic cat/Poland/H266-W/2023 H5N1 2023-06-19 Bydgoszcz**	T	T	G	A	G	G	G	C	**T**	A	G	A	T	T	T	C	C	A	T	G	G	A	G	T	T	A	T	G	C	G	C	A	G	A	C	T	G	C	T	T	C
**A/domestic cat/Poland/H267-W/2023 H5N1 Strzelin**	T	T	G	A	G	G	G	C	G	A	G	A	T	T	T	C	C	A	T	G	G	A	G	T	T	A	T	G	C	G	**T**	A	G	A	C	T	G	C	T	T	C
**A/domestic cat/Poland/H270-W/2023 H5N1 lubelskie**	T	T	G	A	G	G	G	C	**T**	**C**	G	**G**	**C**	T	T	**A**	C	A	T	G	G	**C**	G	T	T	A	T	G	C	G	C	**G**	G	A	C	T	G	C	T	T	C
**A/domestic cat/Poland/H271-W/2023 H5N1 lubelskie**	T	T	G	A	G	G	G	C	G	A	G	A	T	T	T	C	C	A	T	G	G	A	G	T	T	A	T	G	**T**	G	C	A	G	A	C	**C**	G	C	T	T	C
**A/domestic cat/Poland/H277-W1/2023 H5N1 2023-06-26 Namyslow**	T	T	G	A	G	**A**	G	C	G	A	G	A	T	T	T	C	C	A	T	G	G	A	G	T	T	A	T	G	C	G	C	A	G	A	C	T	G	C	T	T	C
**A/domestic cat/Poland/H249/2023 H5N1 2023-06-22 Gdansk**	T	T	G	**G**	**T**	G	G	C	**T**	**C**	G	A	T	T	T	C	C	A	T	G	G	A	G	T	T	A	**C**	G	C	G	C	A	G	A	C	T	G	C	T	T	C
**A/domestic cat/Poland/H248/2023 H5N1 2023-06-15 Pruszcz Gdanski**	T	T	G	A	G	G	G	C	K	A	G	A	T	T	T	C	C	A	T	G	G	A	G	T	T	A	T	G	C	G	C	A	G	A	C	T	G	C	T	T	C
**A/domestic cat/Poland/Kot2/2023 H5N1 2023-06-19 Poznan**	T	T	G	A	G	G	**A**	C	G	A	G	A	T	/	/	/	/	/	/	/	/	A	G	T	T	A	T	G	C	G	C	A	G	A	C	T	**A**	C	T	T	C
**A/domestic cat/Poland/Kot1/2023 H5N1 2023-06-19 Poznan**	T	T	G	A	G	G	G	**T**	G	A	**A**	A	T	T	T	C	C	A	T	G	/	A	G	T	T	A	T	G	C	G	C	A	G	A	C	T	G	C	T	T	**A**
**A/domestic cat/Poland/H254/2023 H5N1 2023-06-22 Lublin**	T	**C**	G	A	G	G	G	C	**T**	**C**	G	A	T	**C**	T	C	C	A	T	G	G	A	G	T	T	A	T	G	C	G	C	A	G	A	C	T	G	C	T	**C**	C
**A/domestic cat/Poland/H252/2023 H5N1 2023-06-22 Lublin**	T	T	G	A	G	G	G	C	G	A	G	A	T	T	T	C	C	A	T	G	G	A	G	T	T	A	T	G	C	G	C	A	**A**	A	C	T	G	C	**C**	T	C
**A/domestic cat/Poland/H247/2023 H5N1 2023-06-20 Gdansk**	T	T	G	A	G	G	G	C	**T**	**C**	G	A	T	T	T	C	C	A	T	G	G	A	G	T	T	A	T	G	C	G	C	A	G	A	C	T	G	C	T	T	C
**A/domestic cat/Poland/H253/2023 H5N1 2023-06-22 Lublin**	T	T	G	A	G	G	G	C	**T**	**C**	G	A	T	T	T	C	C	A	T	G	**A**	A	G	**C**	**C**	A	T	G	C	G	C	A	G	A	C	T	G	C	T	T	C
